# Longitudinal monitoring of honey bee colonies reveals dynamic nature of virus abundance and indicates a negative impact of Lake Sinai virus 2 on colony health

**DOI:** 10.1371/journal.pone.0237544

**Published:** 2020-09-08

**Authors:** Cayley Faurot-Daniels, William Glenny, Katie F. Daughenbaugh, Alexander J. McMenamin, Laura A. Burkle, Michelle L. Flenniken

**Affiliations:** 1 Department of Plant Sciences and Plant Pathology, Montana State University, Bozeman, MT, United States of America; 2 Pollinator Health Center, Montana State University, Bozeman, MT, United States of America; 3 Department of Ecology, Montana State University, Bozeman, MT, United States of America; 4 Department of Microbiology and Immunology, Montana State University, Bozeman, MT, United States of America; University of North Carolina at Greensboro, UNITED STATES

## Abstract

Honey bees (*Apis mellifera*) are important pollinators of plants, including those that produce nut, fruit, and vegetable crops. Therefore, high annual losses of managed honey bee colonies in the United States and many other countries threaten global agriculture. Honey bee colony deaths have been associated with multiple abiotic and biotic factors, including pathogens, but the impact of virus infections on honey bee colony population size and survival are not well understood. To further investigate seasonal patterns of pathogen presence and abundance and the impact of viruses on honey bee colony health, commercially managed colonies involved in the 2016 California almond pollination event were monitored for one year. At each sample date, colony health and pathogen burden were assessed. Data from this 50-colony cohort study illustrate the dynamic nature of honey bee colony health and the temporal patterns of virus infection. Black queen cell virus, deformed wing virus, sacbrood virus, and the Lake Sinai viruses were the most readily detected viruses in honey bee samples obtained throughout the year. Analyses of virus prevalence and abundance revealed pathogen-specific trends including the overall increase in deformed wing virus abundance from summer to fall, while the levels of Lake Sinai virus 2 (LSV2) decreased over the same time period. Though virus prevalence and abundance varied in individual colonies, analyses of the overall trends reveal correlation with sample date. Total virus abundance increased from November 2015 (post-honey harvest) to the end of the almond pollination event in March 2016, which coincides with spring increase in colony population size. Peak total virus abundance occurred in late fall (August and October 2016), which correlated with the time period when the majority of colonies died. Honey bee colonies with larger populations harbored less LSV2 than weaker colonies with smaller populations, suggesting an inverse relationship between colony health and LSV2 abundance. Together, data from this and other longitudinal studies at the colony level are forming a better understanding of the impact of viruses on honey bee colony losses.

## Introduction

Honey bees (*Apis mellifera*) are eusocial insects that live in colonies composed of sterile female worker bees (~ 35,000), hundreds of male bees (drones), and a single reproductive female, the queen bee [1]. Honey bees are important pollinators of plants that produce fruit, nut, and vegetable crops, as well as numerous native and wild plant species [2–4]. It is estimated that insect pollination, primarily carried out by honey bees, is integral for the production of agricultural crops valued at $14.6 billion dollars annually in the US [5], and $175 billion dollars worldwide [2]. The majority of commercially managed honey bee colonies in the US (80% or over 1.8 million colonies) are transported to California to pollinate almond trees that produce over 80% of the global almond crop [6–9]. Annual losses of honey bee colonies in the United States averaged 37% from 2010–2018 [10–20]. Though it is appreciated that multiple abiotic and biotic factors contribute to colony deaths, the impact of viruses on colony health is not well understood. Longitudinal, colony-level monitoring studies that encompass almond pollination are required to better understand the impact of pathogens on high annual honey bee colony deaths in the US.

The majority of honey bee colony losses occur during the winter (i.e., November–February) [10, 14–16, 21–23], though summer losses are also impactful [11–13, 16]. The factors that contribute to high annual honey bee colony deaths include queen failure, agrochemical exposure (both acute and sublethal), management practices (i.e., transportation and treatments), lack of quality forage or poor nutrition, the parasitic *Varroa destructor* mite, and pathogens [14, 23–37]. To maintain an adequate number of colonies for agricultural pollination services, commercial beekeepers employ numerous management strategies including: splitting strong colonies to make up for colony deaths, replacing queen bees, limiting levels of *Varroa destructor* mite infestation with periodic miticide treatment, supplemental feeding, and frame replacement to reduce chemical build up in wax foundation.

Honey bee pathogens include the trypanosomatid *Lotmaria passim* (formerly *Crithidia mellificae* strain sf) [38–40]; fungi (*Ascosphaera apis*, *Aspergillus spp*.); microsporidia *Nosema ceranae* and *Nosema apis* [41], bacterial pathogens (i.e., *Paenibacillus larvae* and *Melissococcus plutonius*) [42, 43] and viruses (reviewed in [44]). Beekeepers are accustomed to detecting and dealing with parasites that are easy to see and/or cause overt symptoms, including *Varroa destructor* mites, which negatively impact colony health by feeding on developing bees (brood) and facilitating virus transmission (reviewed in [45]) [46]. Mite parasitization of developing honey bees can result in physical deformities, reduced body weight, and/or greater deformed wing virus (DWV) levels [30] (reviewed in [45]). Most large-scale commercial beekeepers regularly monitor mite levels in colonies and treat colonies when mite infestation (i.e., the number of mites per 100 bees) is ≥ 3% [14, 22, 47–54]. Bacterial foulbrood disease detection and diagnosis are also facilitated by overt symptoms including diseased larvae with characteristic morphologies and odors [42, 43]. In contrast, viral infections do not always cause overt symptoms and molecular tests are required for diagnoses (reviewed in [44, 55]).

Viruses, specifically positive-sense single-stranded RNA viruses (+ssRNA) in the order *Picornovirales*, are the largest class of honey bee-infecting pathogens (reviewed in [44, 56]. Honey bee infecting viruses include black queen cell virus (BQCV), deformed wing virus (DWV), sacbrood virus (SBV), Israeli acute paralysis virus (IAPV), acute bee paralysis virus (ABPV), Kashmir bee virus (KBV), chronic bee paralysis virus (CBPV) [57], Lake Sinai viruses (LSVs) [26, 58, 59], and a growing list of recently discovered viruses and virus families (reviewed in [44, 56, 60]). Viruses are transmitted vertically from parents to offspring and horizontally between bees within the crowded environment of the colony via contact and trophallaxis (mouth to mouth food transfer), as well as via contact with contaminated floral resources while foraging [61–63] (reviewed in [44, 64]). Transmission of several honey bee viruses including IAPV, DWV-A, and DWV-B (formerly known as Varroa destructor virus-1 (VDV-1)) is mediated by *Varroa destructor* mites, and mite infestation and DWV levels are correlated [65, 66]. Mites may also vector KBV [67, 68] and IAPV [69] (reviewed in [45, 56]). Though mites likely passively transmit numerous viruses, mite infestation levels do not seem to drive the prevalence and transmission of other viruses including BQCV and LSVs [26, 53]. In addition to intra- and inter-honey bee colony virus transmission, many “honey bee viruses” infect a broad range of insects including bumble bees, wasps, and ants (reviewed in [32, 44, 56, 60, 70]) and thus transmission between taxa also impacts virus prevalence and abundances in sympatric bee and insect species.

Detection of viruses in honey bee samples is common, and infection levels can reach over 100 billion copies per bee [26, 71–74]. At these infection levels, viruses are likely energetically taxing and negatively impact honey bee health. Though virus infections are common, their presence and abundance vary and indicates that colonies, and likely individual bees, can reduce infections to below detection levels. Honey bee antiviral defense mechanisms include immune signal transduction cascades (i.e., Toll, Jak/STAT, and Imd pathways) [75, 76] and dsRNA-mediated responses (i.e., RNA interference (RNAi) [77–80] and a non-sequence specific response [81–83]) that limit infections (reviewed in [76, 84]). Effective antiviral responses in individual honey bees are required to ensure survival of the colony.

Several studies indicate that virus prevalence and abundance correlates with poor colony health and colony deaths, though results vary with sample cohort [13, 14, 23, 26, 54, 71, 85, 86]. Deformed wing virus coupled with mite infestation is particularly devastating to honey bee colonies [23, 30, 33]. However, the impact of other viruses, including the Lake Sinai virus family, is less clear; as LSV abundance correlates with weak or CCD-affected colonies in some studies, infection is not statistically associated with colony losses in other sample cohorts [14, 26, 87, 88]. The majority of honey bee monitoring efforts in North America have been carried out at the apiary level rather than the individual colony level [11, 13, 14, 89]. In the US, the Bee Informed Partnership and the USDA-ARS (Beltsville, MD) have tested and reported on numerous honey bee samples [10–20]. The Canadian National Honey Bee Health Survey data includes virus analysis of over 2,500 apiary-level samples (i.e., samples from 10 colonies per apiary) collected between July–September 2017, and indicates a high incidence of BQCV, DWV, LSV2, and SBV [90]. There have been relatively few longitudinal monitoring studies performed at the colony or superorganism level [53, 89, 91, 92]. Previous studies indicate that virus prevalence and abundance varies with sample date (or season) and that peak abundance typically coincides with increased brood rearing and foraging activities during the summer months in temperate climates [53, 54, 85] (reviewed in [44, 55]). However, the degree of variability between studies at both the apiary and colony levels, which are in part due to small sample sizes, methodological differences, timing of sampling, and variabilities in annual virus exposure that differ with geographic location, indicate that additional longitudinal monitoring studies are required to better understand the impact of viruses on honey bee colony health.

Herein we report findings from longitudinal monitoring of 50 commercially managed honey bee colonies involved in the 2016 California almond pollination season. At each of six sampling dates, colony health (using colony population size as a proxy) was assessed and bee samples were obtained for molecular diagnostics of pathogen prevalence and virus abundance. The most prevalent pathogens in this Montana and California-based study were black queen cell virus, deformed wing virus, sacbrood virus, and the trypanosomatid *Lotmaria passim* (formerly known as *Crithidia mellificae*, strain sf). Pathogen prevalence and abundance in honey bee colonies varied throughout the study and were strongly associated with sample date. The total number of pathogens per colony was highest in honey bee samples obtained in April 2016, though overall virus abundance was greatest later in the summer and early fall. In this sample cohort, Lake Sinai virus 2 (LSV2) abundance was lower in strong colonies with larger populations, suggesting a correlation between these viruses and honey bee colony health. Together, data from this and other temporal monitoring studies at the colony level will lead to a better understanding of the impact of pathogens on honey bee colony losses.

## Results and discussion

### Honey bee colony monitoring and pathogen diagnostics

**Fig 1:**
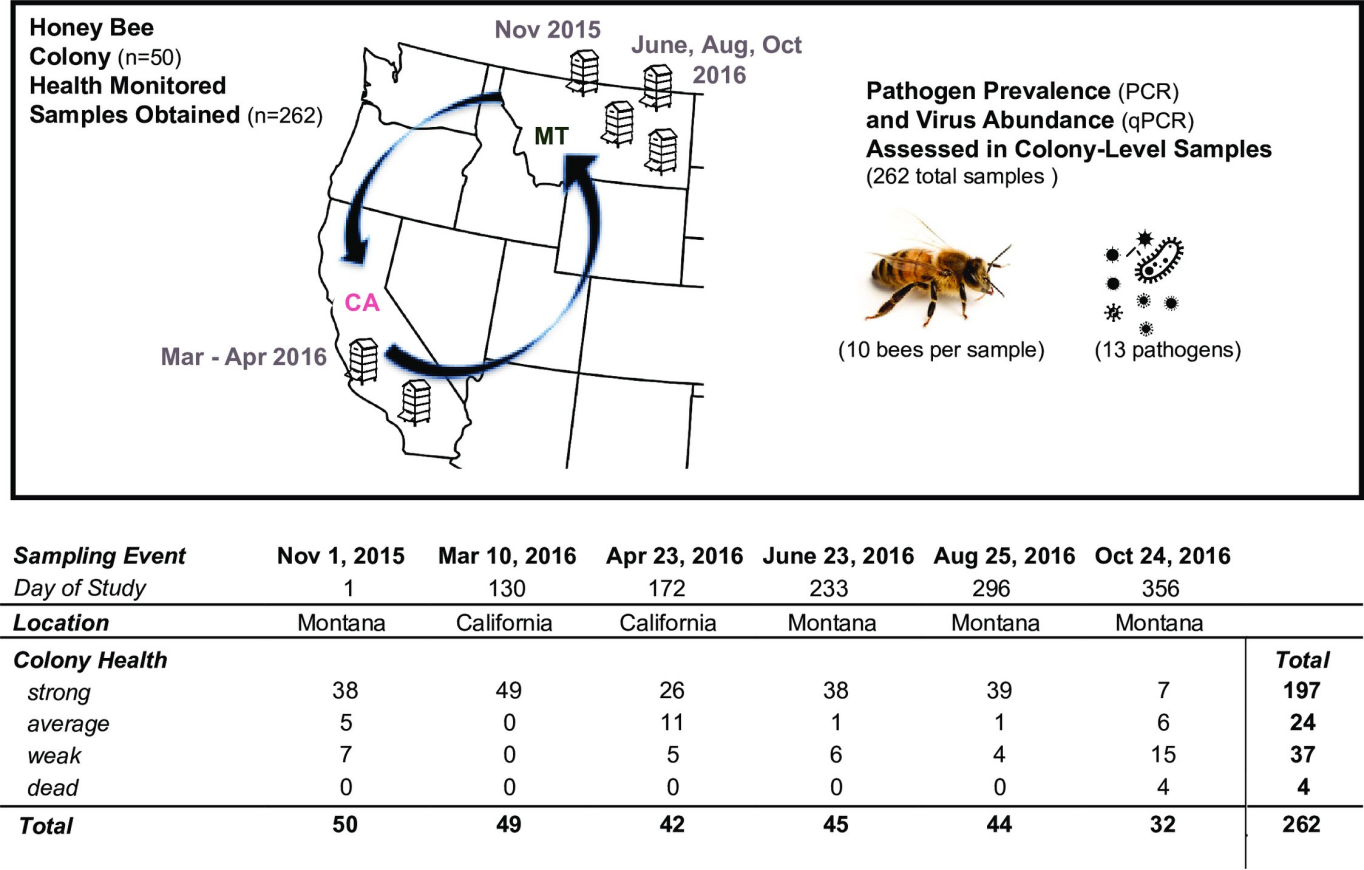
Commercially managed honey bee colonies were longitudinally monitored before, during, and after the 2016 almond pollination season. Honey bee colonies (n = 50) from a single Montana-based commercial beekeeping operation that transports colonies to California for almond pollination were monitored from November 2015 to October 2016. At each sampling event (n = 6), colony health, using colony population size as a proxy for health, was monitored and samples of live honey bees were obtained. Pathogen specific PCR was carried out to assess the prevalence of 13 honey bee pathogens including 11 viruses, the microsporidia *Nosema ceranae*, and the trypanosomatid *Lotmaria passim*, and qPCR was used to assess the abundance of 9 viruses (i.e., BQCV, CBPV, DWV, IAPV, LSV1, LSV2, LSV3, LSV4, and SBV). In total, 262 samples were analyzed, of which 37 were categorized as weak, 24 as average, 197 as strong, and 4 were dead at the time of sampling. Over the course of the study 22 colonies died. During the last sampling event in October 2016, 28 colonies were alive and four dead colonies were sampled (n = 32).

To further investigate seasonal patterns of pathogen presence and abundance and the impact of viruses on honey bee colony health, we collaborated with a Montana-based commercial beekeeping operation that transports approximately 1500 honey bee colonies to California for almond pollination. Colony health and pathogen prevalence and abundance were monitored in a small subset of colonies (n = 50) from November 2015 to October 2016. Colonies were originally located in a single apiary in Montana, then transported to California for the almond bloom, which occurred in late-February / early-March, and subsequently transported back to Montana (Fig 1). At each sampling event, colony health was assessed using colony population size as a proxy for health. Colony health was determined by frame count, or the number of frames that were more than 2/3 covered with bees, and further categorized into health ratings (i.e. weak ≤ 5, average = 6–8, strong ≥ 9) for some analyses [93]. By the end of the study in October 2016, each colony was sampled between three and six times. At each sampling event, live honey bees were obtained for pathogen-specific PCR analysis of the prevalence of 13 pathogens: 11 commonly occurring viruses (i.e., acute bee paralysis virus (ABPV), black queen cell virus (BQCV), chronic bee paralysis virus (CBPV), deformed wing virus (DWV), Israeli acute paralysis virus (IAPV), Kashmir bee virus (KBV), Lake Sinai virus 1 (LSV1), Lake Sinai virus 2 (LSV2), Lake Sinai virus 3 (LSV3), Lake Sinai virus 4 (LSV4), and sacbrood virus (SBV)), and two eukaryotic pathogens (the trypanosomatid *Lotmaria passim*, formerly known as *Crithidia mellificae sf* (*C*.*m*.*/L*.*p*.), and the microsporidial pathogen *Nosema ceranae*) (*Nos*.) [14, 26, 53, 54, 85] (S1 Table). The abundances of the nine most prevalent viruses were assessed by qPCR (S1 Table). In total, 262 samples were analyzed, of which 37 were categorized as weak, 24 as average, 197 as strong, and 4 were dead at the time of sampling (Fig 1 and S1 Table). Over the course of the study 44% (n = 22) of the monitored colonies died, slightly more than the 37% average annual colony losses reported between 2010–2018 [10–20]. In this work, we use “pathogen incidence” to refer to the frequency of detection of a particular pathogen in the sample cohort, “pathogen prevalence” to indicate pathogen detection in the sample cohort, “total pathogen prevalence” for the sum of the different pathogens detected in a sample, and “pathogen abundance” to describe viral RNA abundance. “Pathogen composition” is used to refer to the abundance of all co-infecting pathogens in a sample. Therefore, both pathogen prevalence and abundance contribute to the pathogen composition of each colony throughout the course of the study.

### Seasonal variation in pathogen distribution and incidence is independent of colony health

**Fig 2:**
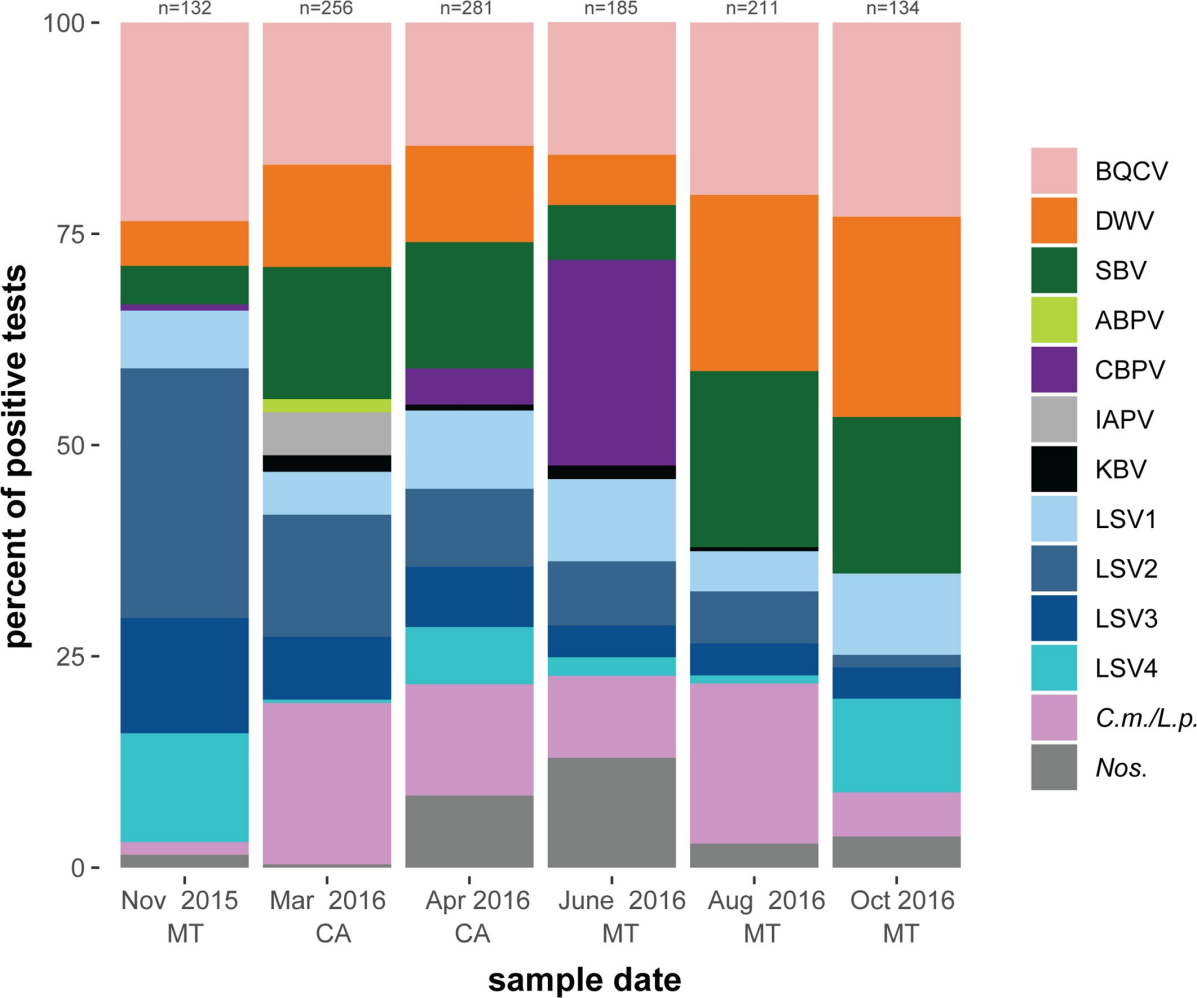
Distribution of honey bee pathogens detected in monitored colonies by sampling event. Pathogen specific PCR was used test honey bee samples (n = 262) for 13 commonly occurring pathogens (i.e., ABPV, BQCV, CBPV, DWV, IAPV, KBV, LSV1, LSV2, LSV3, LSV4, SBV, *Nos*., and *C*.*m*.*/L*.*p*.) (S1 Table). The distribution of positive tests for each pathogen is shown as a percentage of all positive tests from all colonies (n≤50) at each sample date (i.e., November 2015 = 132 positive tests, March 2016 = 256 positive tests, April 2016 = 281 positive tests, June 2016 = 185 positive tests, August 2016 = 211 positive tests, and October 2016 = 134 positive tests).

To examine seasonal trends in honey bee pathogen burden and investigate potential correlations with colony health, pathogen prevalence in monitored honey bee colonies (n = 262 samples) was determined by pathogen-specific PCR for 13 commonly occurring pathogens (i.e., ABPV, BQCV, CBPV, DWV, IAPV, KBV, LSV1, LSV2, LSV3, LSV4, SBV, *Nos*., and *C*.*m*.*/L*.*p*.; S1 Table). Graphical representation of that data represented as a percentage of all positive tests (n = 1199), and as a percentage of positive tests for each colony health rating (i.e., strong = 889, average = 127, and weak = 172 positive tests) illustrates that the most readily detected pathogens were BQCV, DWV, SBV, and *C*.*m*.*/L*.*p* and that pathogen distribution was similar across all colony health ratings (S1 Fig). Pathogen distribution was also graphed by sampling date with corresponding location (Fig 2). The greatest number of positive tests (n = 281) were obtained from honey bee samples collected in April while colonies were located in California. This sampling date corresponded with the spring buildup in honey bee colony population size due to increased brood rearing activities. In June 2016, CBPV contributed to 24% of the pathogen distribution while it was relatively absent in all other time points. This relatively short detection of CBPV in the spring is consistent with data from a colony level assessment of virus abundance in southwest Germany [74]. At the beginning of the study in November 2016, the Lake Sinai viruses (LSV1-LSV4) together accounted for 64% of the total pathogen distribution, while the three next highest distributed pathogens (BQCV, DWV, and SBV) accounted for 33% of the pathogen distribution. By the end of the study in October 2016, the opposite was observed, with BQCV, DWV, and SBV together contributing to 65% of the pathogen distribution and the Lake Sinai viruses together accounting for 26% of the pathogen distribution (Fig 2).

**Fig 3:**
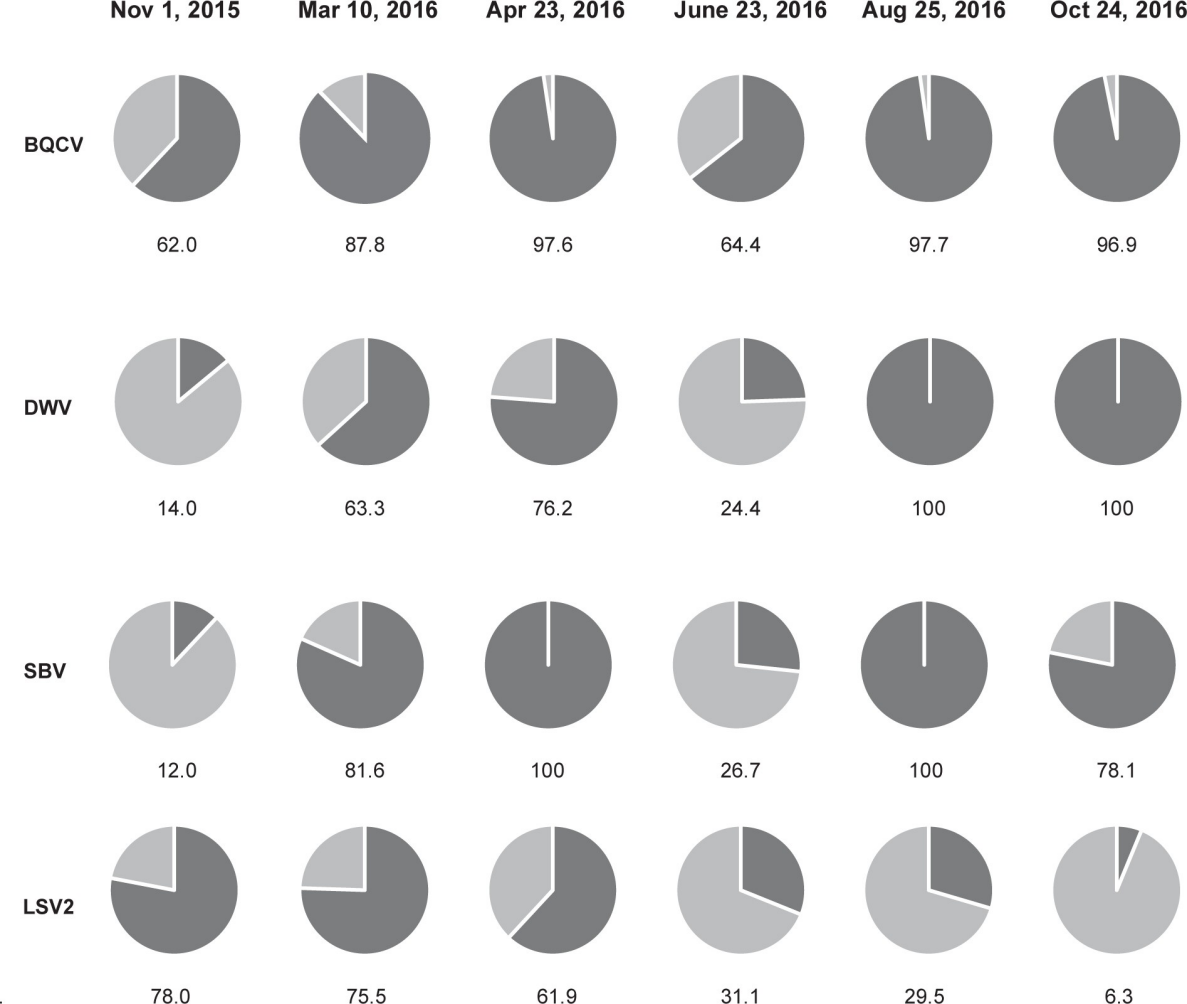
Pathogen incidence in honey bee colonies throughout the study. The observed incidence of the four most prevalent pathogens in this study (i.e., BQCV, DWV, SBV, and LSV2) are represented as a percentage of all samples at each time point (dark gray = positive).

Pathogen incidence, defined as the proportion of colonies that tested positive for a pathogen at each sampling event, varied over the course of this study (Fig 3 and S2 Fig). The incidence of the most commonly detected pathogens in this sample cohort (i.e., BQCV, DWV, and SBV) increased from November 2015 to April 2016, was lower in June and increased again for the remainder of the study. In contrast, LSV2 incidence decreased steadily from a height of 78% incidence in November 2015 to 6.3% incidence at in October 2016 (Fig 3 and S2 Fig).

### Total pathogen prevalence highest in the spring

To examine if total pathogen prevalence (i.e., the total number of pathogens detected in each sample) correlated with colony health and/or varied throughout the course of the year PCR results for 13 pathogens (i.e., ABPV, BQCV, CBPV, DWV, IAPV, KBV, LSV 1–4, SBV, *L*. *passim*, and *N*. *ceranae*) were evaluated using a generalized linear mixed effect model (GLMM) with a Poisson family distribution and main effects of sampling time point and colony health rating (i.e., weak, average, strong, or dead) (S1 Table). The observed mean pathogen prevalence in strong colonies was 4.9 pathogens, in average colonies 5.5 pathogens, in weak colonies 5.1 pathogens, and in dead colonies 3.5 pathogens (S3 Fig). The unequal distribution of colony health ratings in this sample cohort was not ideal for statistical analyses but indicated that mean total pathogen prevalence did not differ by colony health rating.

**Fig 4:**
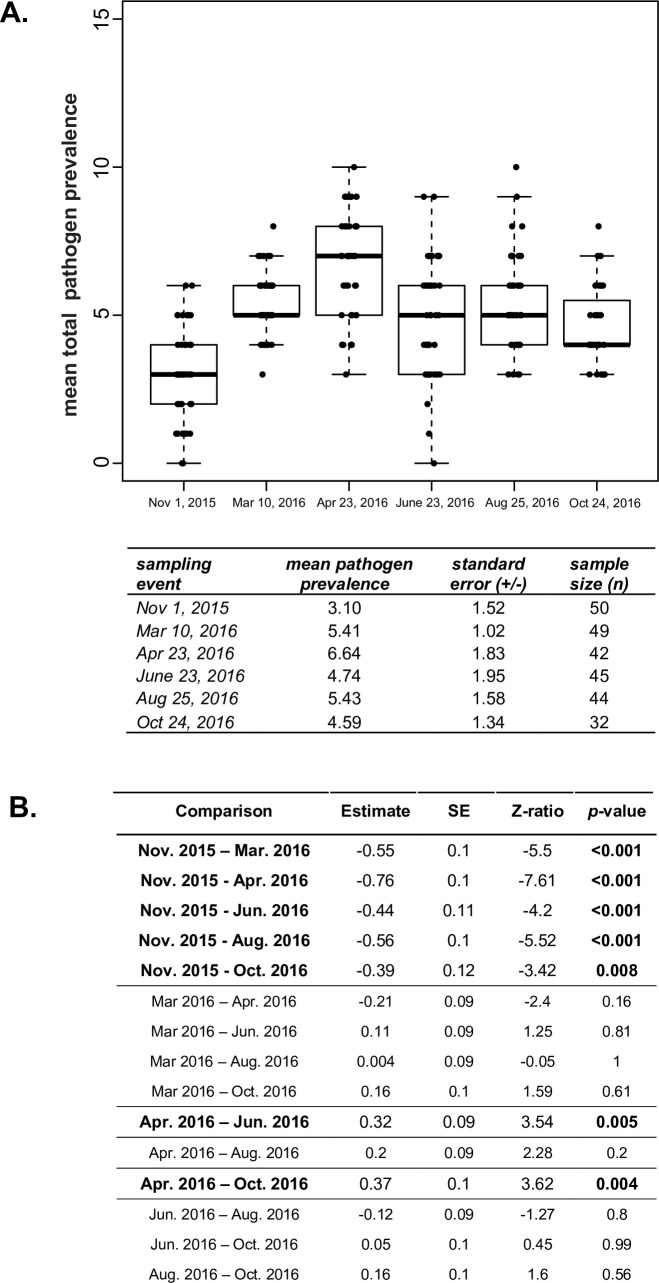
Mean total pathogen prevalence is highest in the spring. A. The observed mean pathogen prevalence was lowest at the onset of the study (November 2015) and highest just after almond pollination (April 2016). Honey bee samples were obtained from monitored colonies (n≤50) on six sampling dates over the course of one year (November 2015 to October 2016) and were tested for the presence of 13 pathogens. The total observed pathogen prevalence was determined by summing the number of different pathogens detected in each sample and the mean number of pathogens per sampling event and associated standard error were calculated. B. The estimated change in mean total pathogen prevalence in honey bee colonies between sampling dates was compared using a generalized linear mixed effect model (GLMM) with Tukey’s adjusted, pairwise, comparison. The estimated log fold-change of the total mean pathogen prevalence between sampling events (estimate) was greater in all five time points following the onset of the study in November 2015 and peaked in April 2016 after the late February, early March almond bloom. Significant comparisons (*p*-value < 0.05) are bolded.

Sample date, and in turn season, was associated with differences in the mean total pathogen prevalence in individual colonies (Fig 4, S1 Table), a result in-line with previous studies indicating that the incidence and prevalence of honey bee viruses vary over time and is typically greater in the spring and summer [14, 47–49, 53]. Colonies had the lowest observed mean total pathogen prevalence with an average of 3.1 pathogens at the onset of the study in November 2015 (Fig 4A). In March 2016, just after the late February almond bloom, the observed mean total pathogen prevalence was 5.4 pathogens and in April 2016, while the colonies were still in California, the mean total pathogen prevalence reached a height of 6.6 pathogens (Fig 4A). Pairwise differences in the observed mean pathogen prevalence for colonies at different sampling events was statistically evaluated using a GLMM with a Poisson family distribution, followed by a Tukey’s adjusted post-hoc test. The estimated mean pathogen prevalence between sampling dates was greater in all five time points following the onset of the study in November 2015. Pathogen prevalence in April 2016 was estimated to be 0.33 (SE +/- 0.09) times greater than in March 2016 (Fig 4B). This height in pathogen prevalence may be partially attributed to potential increased exposure to pathogens due to spring foraging, including almond pollination, and also correlates with buildup of honey bee colony populations at this time of year. Previous studies indicate that honey bee colonies may be better able to withstand greater pathogen burdens in the spring, since strong colonies had a high pathogen prevalence at that time of year. Whereas in winter, weak colonies had a greater pathogen burden than strong colonies [54].

### Virus abundance varies with season

**Fig 5:**
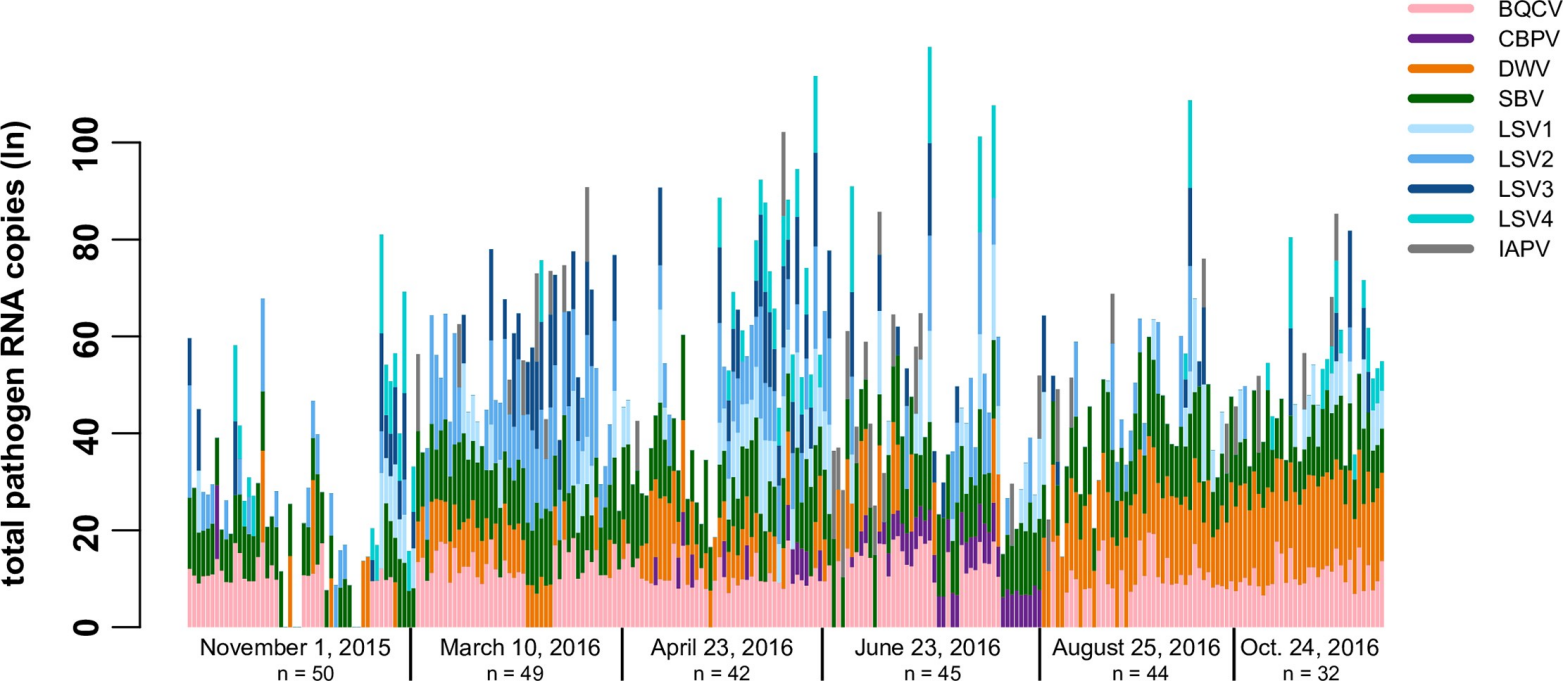
Longitudinal assessment of honey bee pathogen abundance at the colony level. Total viral RNA abundance (ln) per colony at each sample date is represented by a vertical bar. Quantitative PCR was used to determine the RNA copies of each virus in colony level samples (S1 Table). Natural log transformed qPCR values for each virus are represented as colored segments of each vertical bar representing a sample from a single colony: BQCV (pink), CBPV (purple), DWV (orange), SBV (green), LSV1 (light blue), LSV2 (medium blue), LSV3 (dark blue), LSV4 (teal), and IAPV (gray). In this sample cohort, the most prominent observations are increased detection and abundance of LSV2 earlier in the study (March 2016 and April 2016) and DWV later in the study (August 2016 and October 2016). The greatest total pathogen RNA abundance values were observed in the middle of the study (April 2016 and June 2016).

To examine the relationships between virus abundance, colony health, and sampling date, virus-specific qPCR was used to quantify the abundance of the nine most prevalent viruses (i.e. BQCV, DWV, CBPV, IAPV, LSV1, LSV2, LSV3, LSV4, and SBV). Quantitative PCR values ranged from 0 to over 1.2 x10^10^ viral RNA copies, including both genomic and transcript RNA (S1 Table). To visualize overall trends in virus abundance, each of the nine viruses is presented as part of the sum total for each colony at each time point (Fig 5). The largest total virus abundance occurred between April 2016 and June 2016 (Fig 5). Overall, the incidence and abundance of deformed wing virus (DWV) was greatest in the last two sampling events (i.e., August and October 2016). Lake Sinai virus 2 (LSV2) abundance decreased over the course of the study, whereas levels of black queen cell virus (BQCV) and sacbrood virus (SBV) were constant throughout the study (Fig 5 and S1 Table). The high prevalence of LSVs, particularly LSV2, detected in the winter and/or early spring in this study, corresponds with data from other Montana and California based sample cohorts [53, 54, 71], whereas other studies in Germany [74] and the US [14] detected the majority of LSVs in the summer. Consistent detection of moderate levels of BQCV throughout the year and greater abundance of DWV in the late summer and early fall corresponds with several previous reports from the US, Canada, and Germany [23, 53, 54, 71, 74, 90].

To normalize the distribution of qPCR values for statistical analyses, the data were natural log+1 transformed resulting in a distribution of values that were zero if the pathogen was below the detection threshold or a normal distribution of continuous values if the pathogen was above a detection threshold (S1 Table). Therefore, qPCR data were analyzed using a two-step approach, first using binary response values of pathogen presence and absence, and second using only the continuous response values (i.e., virus RNA copy numbers) from virus-positive samples. The binary response data were used to estimate the log-odds that a virus would be detected in a colony in response to day of the study and strength rating using a generalized linear mixed effects model (GLMM) with a binomial family distribution and random effect for individual colony to account for resampling colonies [94]. The odds of detecting each of the following nine viruses: BQCV, CBPV, DWV, IAPV, LSV1, LSV2, LSV3, LSV4, and SBV were generally not influenced by colony health rating (i.e. weak, average, strong, or dead) with the exception of LSV4. The odds of detecting LSV4 in strong colonies decreased by a factor of 0.34 (SE +/- 0.58) compared to weak colonies (Z-test, z-value = -2.343, p-value < 0.05). This result indicates that LSV4 is less frequently detected in strong colonies and is worth exploring further, since there is very little data on LSV4 [26].

**Fig 6:**
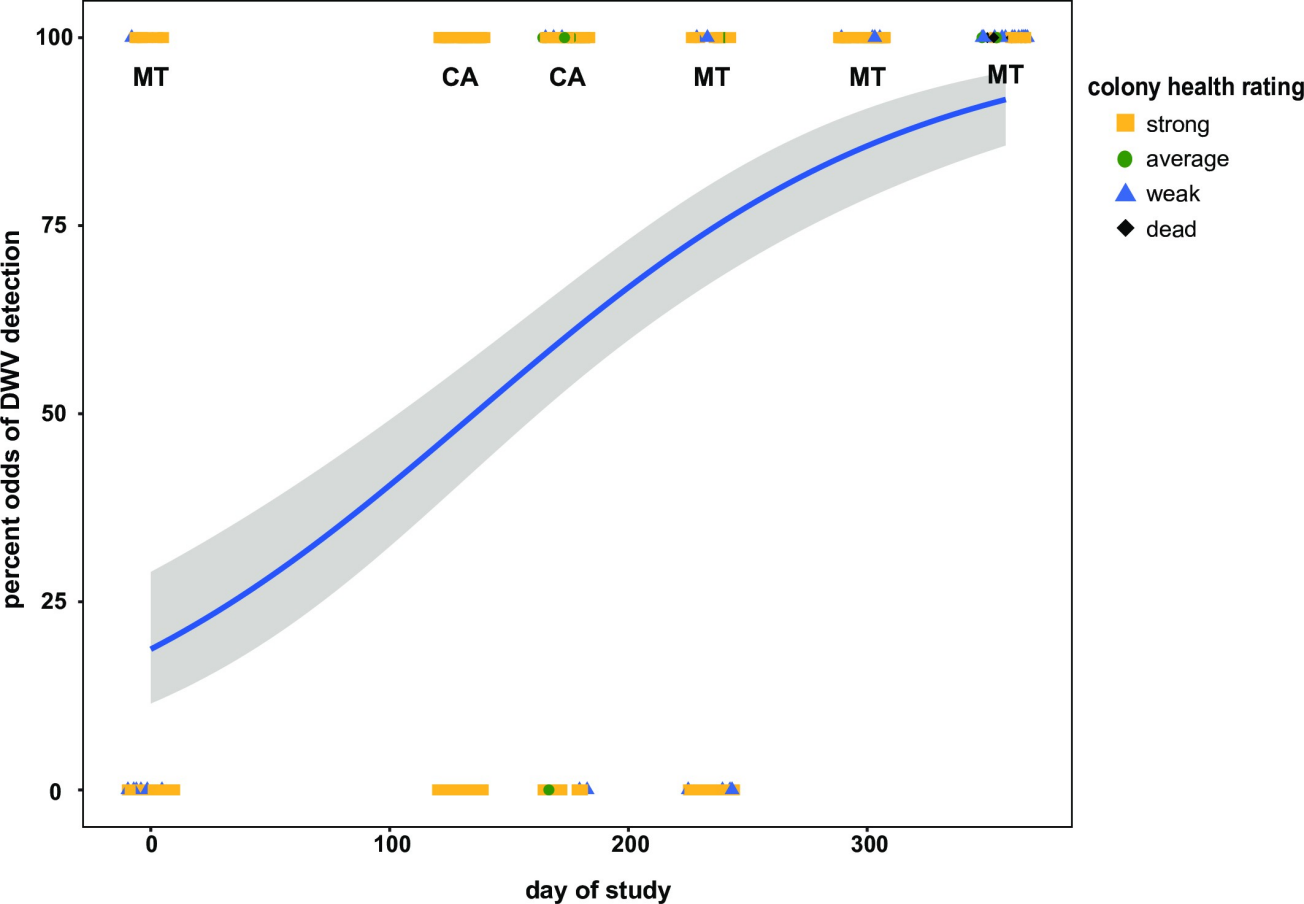
Odds of DWV detection over the course of the study. DWV is more likely to be detected in honey bee colonies later in the monitoring period. The odds of detecting DWV increased by 0.010 (SE +/- 0.001) with each day of the study (Z-test, z-value = 7.092, p-value = 0.0001). The estimated best fit line (blue) represents the probability of detecting DWV in response to the day of study increases across the duration of the study as represented by the positive slope. The best fit line was determined using a generalized linear mixed effect model (GLMM) and is surrounded by upper and lower standard error estimates (gray). Colony level DWV data for each sample date is represented as colony strength indicating icons (i.e., strong = yellow square, average = green circle, weak = blue triangle, and dead = black diamond).

Sample date, or season, influenced the odds of detecting 7 of the 9 honey bee infecting viruses monitored in this study (Fig 6 and S4–S11 Figs). For example, the odds of detecting DWV increased by a factor of 0.010 (SE +/- 0.001) with each day of the study (Z-test, z-value = 7.092, p-value = 0.0001) (Fig 6). This is likely in part due to the time frame of this study and several aspects of honey bee and *Varroa destructor* mite biology. Sampling was initiated after honey harvest and miticide treatment (i.e., thymol) in November 2015 in Montana. At that time of year, there is little to no honey bee brood present in the colony, and thus limited to no *Varroa destructor* mite reproduction (reviewed in [45, 95–97]). Though DWV infections are not solely reliant on mite-mediated transmission, they are often positively correlated [23, 33, 53, 66, 98–100]. Typically, mite infestation levels increase over the course of the spring and summer months, during the time of maximum honey bee brood production, such that mite levels are at their greatest in early fall, prior to honey harvest [45, 74, 97, 101–103]. The last sampling date in this study was at the time of honey harvest, and thus mite levels were likely highest for this sample cohort. However, this is speculative since mite data were not obtained for these samples. In general, mite infestation levels in this beekeeping operation were maintained below the treatment threshold of 3% infestation with periodic miticide treatments. Greater DWV detection in the fall was also observed in apiary level studies including The German Bee Monitoring Project [23], the Canadian National Honey Bee Health Survey [90], and the USA Bee Informed Partnership [14], as well as in colony level studies [53, 54]. Similarly, albeit less dramatically, the odds of detecting several other viruses including: SBV, CBPV, IAPV, and BQCV increased with each day of the study (S4–S7 Figs). In contrast, the probability of detecting LSV2 decreased by a factor of 0.004 (SE +/- 0.001) per day (Z-test, z-value = -3.098, p-value = 0.007) (S8 Fig). The odds of detecting LSV1, LSV3, and LSV4 in a colony remained similar throughout the study (S9–S11 Figs).

**Fig 7:**
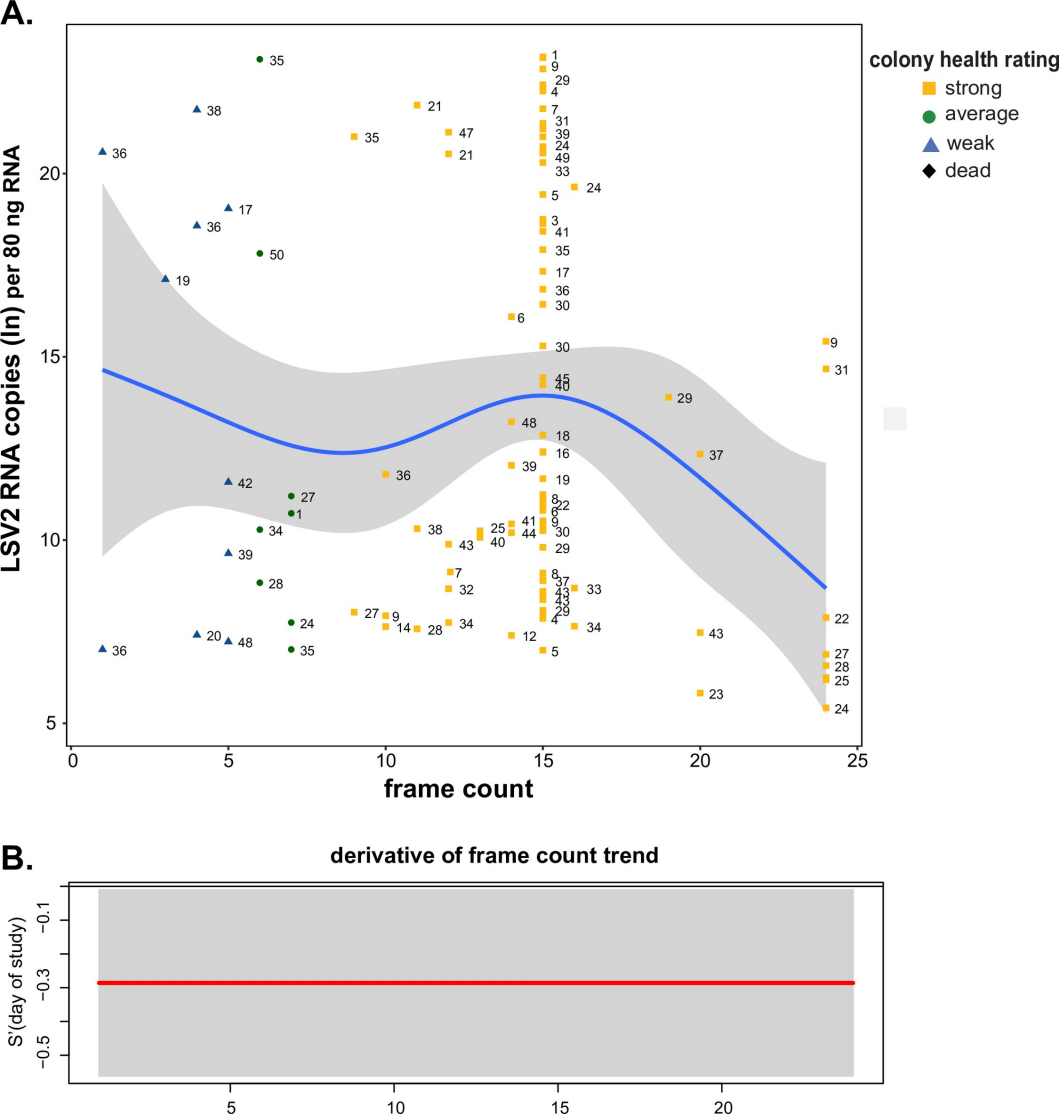
LSV2 abundance is greater in weak colonies. A. LSV2 abundance decreased with increased frame count (health) of honey bee colonies (frame count_edf_ = 1.00, p-value = 0.008). The natural log transformed values of the relative LSV2 abundance as determined by qPCR (y-axis) in honey bee samples were plotted by frame count (x-axis). The best fit line (blue) was determined by a generalized additive mixed model (GAMM) and is surrounded by upper and lower standard error estimates (gray). Colony level LSV2 data for each sample date is represented as colony strength indicating icons (i.e., strong = yellow square, average = green circle, weak = blue triangle, and dead = black diamond) with unique colony identifier numbers, which illustrate the changes in virus abundance of individual colonies throughout the study. B. The first derivative of the fitted spline in panel A was calculated to identify the rate of change of LSV2 abundance with respect to frame count and 95% confidence intervals (gray) were built around the first derivative to distinguish periods of time when the change in virus abundance is significantly different from zero. LSV2 abundance decreased (red) continually with increasing frame count.

**Fig 8:**
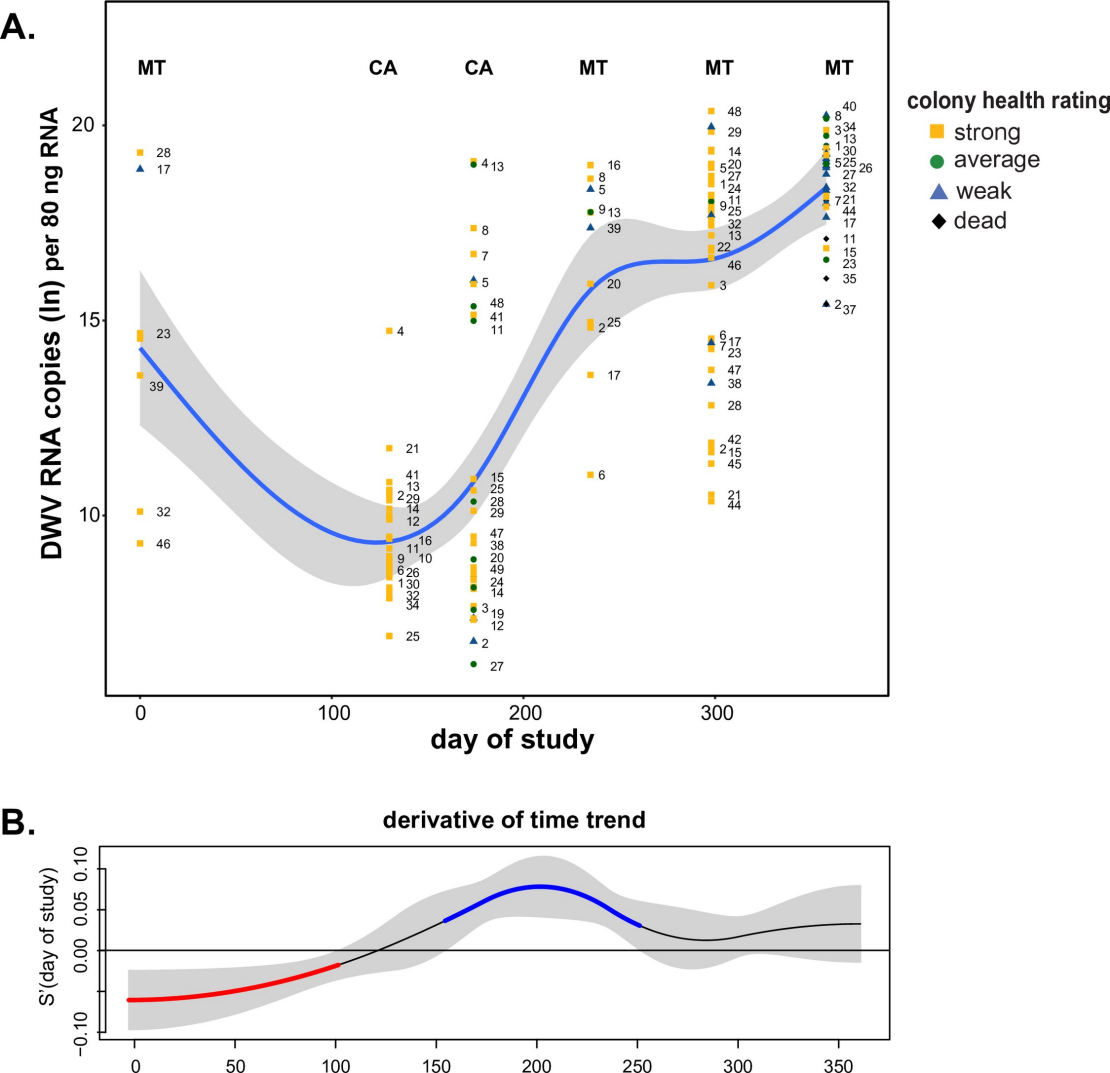
DWV abundance variable over time and greatest in October. A. DWV abundance varied over time and was greatest at the end of the study (day of study_edf_ = 4.253, p-value = 0.0001). The natural log transformed DWV abundance data as determined by qPCR in honey bee samples were plotted by day of study. The best fit line (blue) for DWV abundance over the course of the study was determined by a generalized additive mixed model (GAMM) and is surrounded by upper and lower standard error estimates (gray). Colony level DWV data for each sample date is represented as colony strength indicating icons (i.e., strong = yellow square, average = green circle, weak = blue triangle, and dead = black diamond) with unique colony identifier numbers, which illustrate the changes in virus abundance of individual colonies throughout the study. B. The first derivative of the fitted spline in panel A was calculated to identify the rate of change of DWV abundance throughout time and 95% confidence intervals (gray) were built around the first derivative to distinguish time periods when the change in virus abundance is significantly different from zero. DWV abundance significantly decreased (red) from 0 to 100 days of the study and significantly increased (blue) from 150 to 250 days of the study.

Virus abundance in monitored honey bee colonies was also evaluated using a second approach that examined the dynamic and non-linear trends in abundance with colony health and sample date. For this approach, samples with no detectable virus (i.e., the zero values) were removed on a per-virus basis and the normally distributed data were evaluated using generalized additive mixed models (GAMM) (Figs 7 and 8, and S12–S19 Figs). For this model, the continuous variable of frame count was used to estimate colony health rather than categorical health rating. Lake Sinai virus 2 was the only virus that exhibited a relationship with colony heath. Specifically, LSV2 abundance decreased as frame count, or colony population size, increased (frame count_edf_ = 1.00, p-value = 0.008), (Fig 7). This result indicates that healthier colonies had less LSV2 than weaker colonies, which is supported in previous results demonstrating that weak and/or CCD-affected honey bee colonies had greater LSV2 levels compared to heathier colonies with larger populations [26, 53].

Virus abundance varied with sample date for the majority of viruses monitored in this study including DWV, which was the most abundant virus in this sample cohort (day of study_edf_ = 4.253, p-value < 0.001) (Fig 8). DWV abundance exhibited two periods of significant change, as indicated by the derivative of the estimated GAMM (Fig 8B). Specifically, the rate of change in DWV abundance decreased from 0–100 days of the study and then increased from 150–250 days of the study (Fig 8). The time period between 0 and 100 days (i.e., November 2015 and March 2016) of the study encompasses the transport of colonies from Montana to California for almond pollination, which is noteworthy since reports on pathogen abundance before and after transportation events have indicated that pathogen load may increase [92, 104]. In addition, prior to this transportation event, all colonies in this study were treated with miticides to reduce *Varroa destructor* mite infestation levels. After March 2016, DWV abundance increased and continued to do so until the end of monitoring in October 2016. It is notable that while DWV abundance increased by nearly 100 million copies per 80 ng total RNA from April to June 2016 (i.e. 4.53 x 10^8^ to 5.51 x 10^8^ RNA copies) (S1 Table and Fig 8), DWV incidence decreased from 76.2% in April to 24.4% in June 2016 (Fig 3).

Lake Sinai virus 2 (LSV2) abundance was also influenced by sample date (day of study_edf =_ 4.281, p-value < 0.001) and had more than one significant trend in abundance over the course of the study. However, LSV2 abundance trends were nearly opposite to those of DWV. Specifically, LSV2 abundance increased between 0–100 days of the study (Nov. 2015 –March 2016), and decreased between 100 and 175 days (March -May 2016) and from 300–350 days of the study (mid-August–mid-October 2016) (S12 Fig).

Sample date also influenced LSV4 abundance (day of study_edf =_ 4.071, p-value < 0.001), SBV abundance (day of study_edf_ = 2.252, p-value = 0.003), and IAPV abundance (day of study_edf_ = 1, p-value = 0.027). Abundance values for LSV4 fluctuated throughout the study, including increased abundance between 175 to 225 days and decreased abundance from 275 to 325 days of the study (S13 Fig). The abundance of SBV increased in the beginning of the study from 0 to 100 days but did not significantly change in abundance for the remainder of the study (S14 Fig). IAPV abundance decreased linearly over the course of the study (S15 Fig).

Sample date did not influence trends in abundance of BQCV, LSV1, or LSV3.

Black queen cell virus was highly abundant throughout the study, and though values fluctuated slightly, the changes were not significant (day of study_edf_ = 1.024, p-value = 0.911). Though LSV3 and LSV1 abundance in individual colonies varied widely, the overall abundance trend varied little throughout the study (S16–S18 Figs).

**Fig 9:**
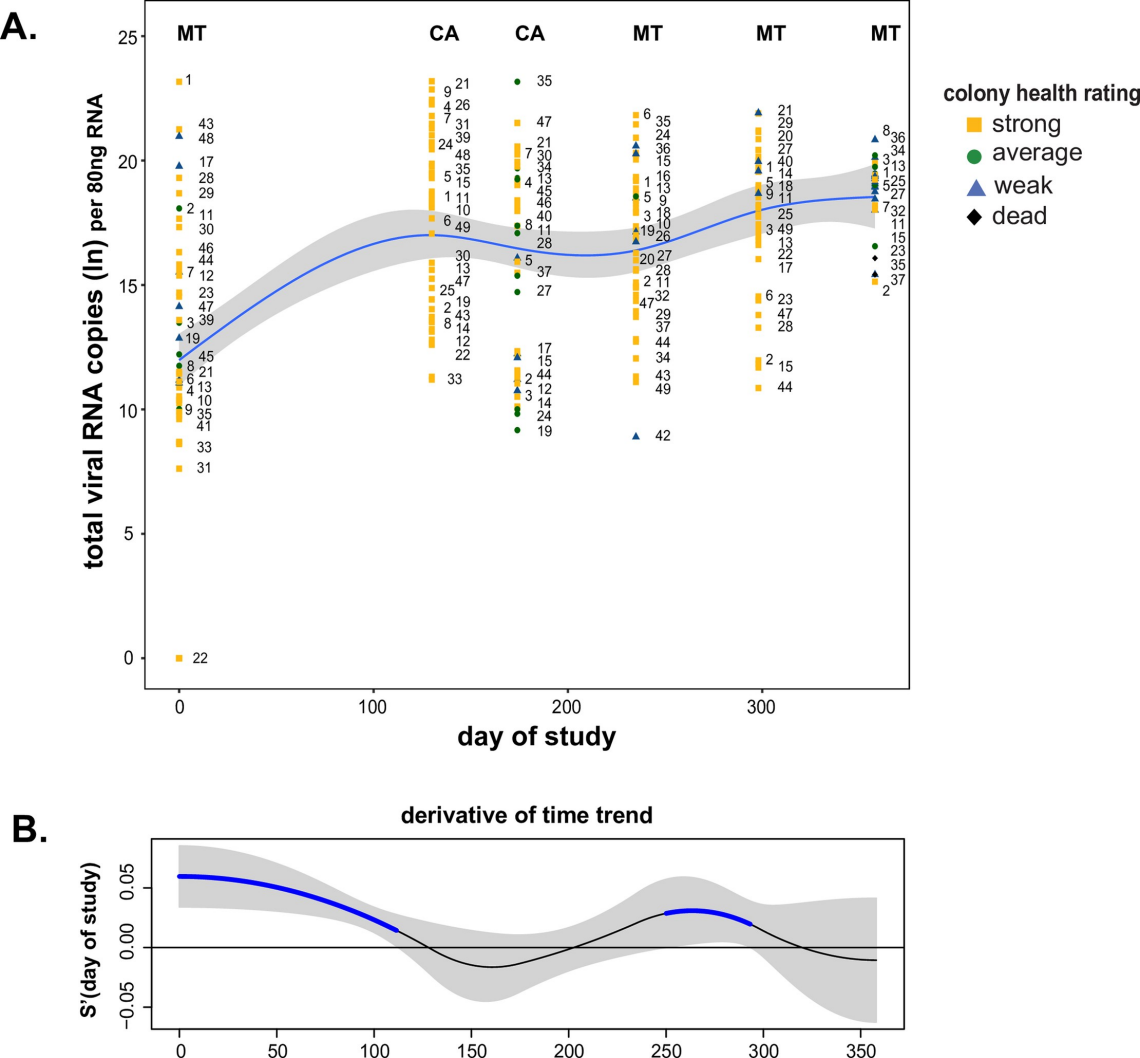
Changes in the total virus RNA abundance in honey bee colonies throughout the study. A. The total pathogen RNA abundance increased over the course of the study (day of study_edf_ = 3.474, p-value < 0.001). The natural log transformed values of the total RNA abundance as determined by qPCR in honey bee samples were plotted by date of sample collection. The best fit line (blue) was determined by a generalized additive mixed model (GAMM) and is surrounded by upper and lower standard error estimates (gray). Colony level data for each sample date is represented as colony strength indicating icons (i.e., strong = yellow square, average = green circle, weak = blue triangle, and dead = black diamond) with unique colony identifier numbers, which illustrate the changes in virus abundance of individual colonies throughout the study. B. The first derivative of the fitted spline in panel A was calculated to identify the rate of change of total pathogen RNA abundance throughout the timeframe and 95% confidence intervals (gray) were built around the first derivative to distinguish periods of time when the change in virus abundance is significantly different from zero. Total virus RNA abundance significantly increased (blue) from 0 to 100 days of the study and from 250 to 300 days of the study.

While the abundances of some viruses (e.g., BQCV) did not dramatically change over time, their infections contribute to the total viral load and may affect the overall fitness of the colony. Colonies examined in this study tested positive for as many as 10 pathogens at one time and as many as eight of the nine viruses quantified by qPCR (S1 Table). Thus, to further understand the impact of total viral load at the colony level, the abundances of all nine viruses were summed, natural log (ln) transformed, and modeled (Fig 9). Similar to the results obtained for specific viruses, total virus abundance depends on sample date (day of study_edf_ = 3.474, p-value < 0.001) (Fig 9) (S1 Table). There were two periods of significant increase in viral load, from 0 to 100 days of the study, which encompassed the time frame of transportation from Montana to California, and again from 250 to 300 days of the study (Fig 9).

Though examination of the overall trend is useful, it is notable that although total abundance increased over time, total abundance in individual colonies varied widely within and between sample dates (Fig 9). For example, from 130 to 250 days of the study the trend in total abundance did not differ (Fig 9B). However, the total abundance of colony #24 had a natural log transformed value of 20 on day 130 (March 2016), a value of 9 on day 172 (April 2016), and back to a value of 20 on day 233 (June 2016), fluctuating well above and below the trendline in consecutive sampling events (Fig 9). Furthermore, this analysis also illustrates that individual colony health rating changes over time. For example, colony #18 started as weak at the onset of the study in November 2015, then was rated as strong from March to June 2016, was average in August 2016 and died by the October 2016 sampling date (S1 Table). Furthermore, colony health (i.e., frame count) did not correlate with sample date. Together these data underscore the importance of longitudinal monitoring, as pathogen prevalence and abundance and colony health vary over time.

### Honey bee pathogen community composition varies with sample date

**Fig 10:**
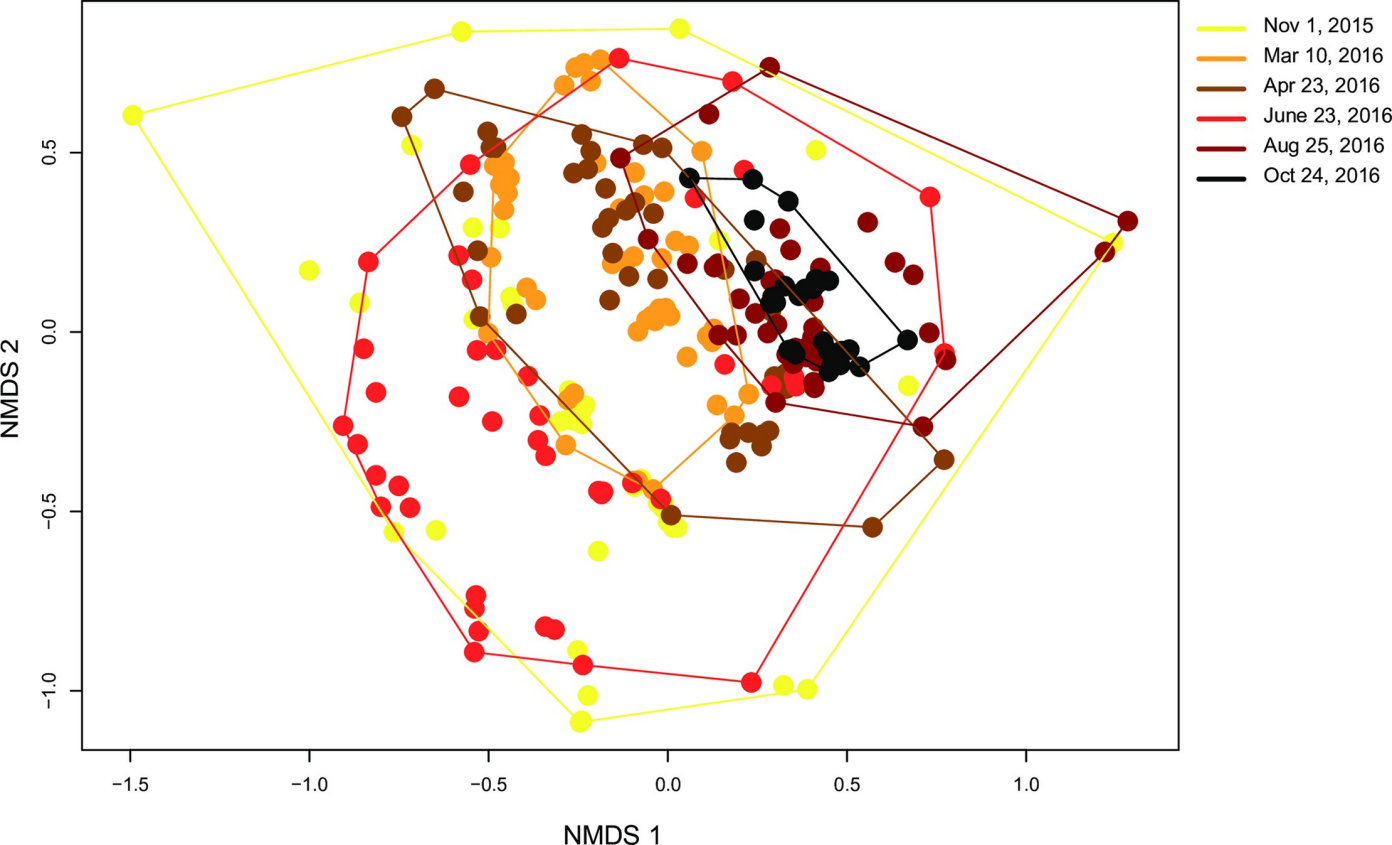
Relative virus composition of honey bee colonies varies by sample date. The virus community composition varied by sampling date (PERMANOVA, F_1,255_ = 26.90, p-value = 0.001). The community composition for each sample (i.e., the log natural transformed abundance of BQCV, CBPV, DWV, SBV, LSV1, LSV2, LSV3, LSV4, and IAPV, as assessed by qPCR) was compared in relation to sample date (i.e., November 2015 (yellow), March 2016 (orange), April 2016 (brown), June 2016 (red), August 2016 (maroon), October 2016 (black)). The position of each point indicates the virus composition of each sample relative to all other samples (i.e., samples with more similar virus composition are closer), calculated using a Bray-Curtis dissimilarity index and plotted on a non-metric multidimensional scaling (NMDS) plot. A homogeneity of community variance test (BETADISPER) was conducted and the mean dispersion (distance to centroid) is significant with respect to the day of the study (F_5,250_ = 8.154, p-value < 0.0001). Pathogen communities were more variable in March 2016 (mean = 4.90, CI = 1.06 to 8.76), April 2016 (mean = 5.33, CI = 1.36 to 9.31), and June 2016 (mean = 7.43, CI = 3.51 to 11.35) compared to the final sampling time point in October 2016.

To assess how virus community composition changed with respect to colony health and sampling date, non-metric multi-dimensional scaling (NMDS) plots were used to examine differences in BQCV, CBPV, DWV, IAPV, LSV1, LSV2, LSV3, LSV4, and SBV abundance in colonies relative to all other colonies. Consistent with the pathogen prevalence analyses, virus community composition in honey bee colonies was not associated with colony health rating (PERMANOVA, F_3,255_ = 1.35, p-value = 0.191, S19 Fig). Whereas, honey bee virus community composition differed by sample date (PERMANOVA, F_1,255_ = 26.90, p-value = 0.001, Fig 10). To better understand the variance in pathogen communities across sampling events, results from a homogeneity of community variance test (BETADISPER) suggests the mean dispersion (distance to centroid) is significant with respect to the day of the study (F_5,250_ = 8.154, p-value < 0.001). Variation in pathogen communities was higher in March 2016 (mean = 4.90, CI = 1.06 to 8.76), April 2016 (mean = 5.33, CI = 1.36 to 9.31), and June 2016 (mean = 7.43, CI = 3.51 to 11.35) compared to the final sampling time point in October 2016, when pathogen communities across all colonies in the study were more similar.

A similarity percentage (SIMPER) analysis was conducted to assess which viruses contributed most to changes in virus community composition between each sampling date. In all, there are 15 comparisons between time points, and the cumulative dissimilarity for the top three viruses in each comparison are reported as a percentage (S4 Table). Deformed wing virus, LSV2, and BQCV most frequently accounted for the highest percentage of dissimilarity of virus communities between time points (S4 Table). Specifically, BQCV, DWV, and SBV primarily accounted for the dissimilarity between virus communities at the onset of the study in November 2015 and the final two sampling periods at the end of the study (i.e., 63% in August and 62% in October 2016) (S4 Table). When comparing the virus communities between the two time points while colonies were in California, the top three viruses contributing to dissimilarity were all Lake Sinai viruses, with LSV2, LSV1, and LSV3 accounting for 49% of the dissimilarity between March and April 2016.

### Pairwise pathogen interactions reveal positive correlations between Lake Sinai viruses

**Fig 11:**
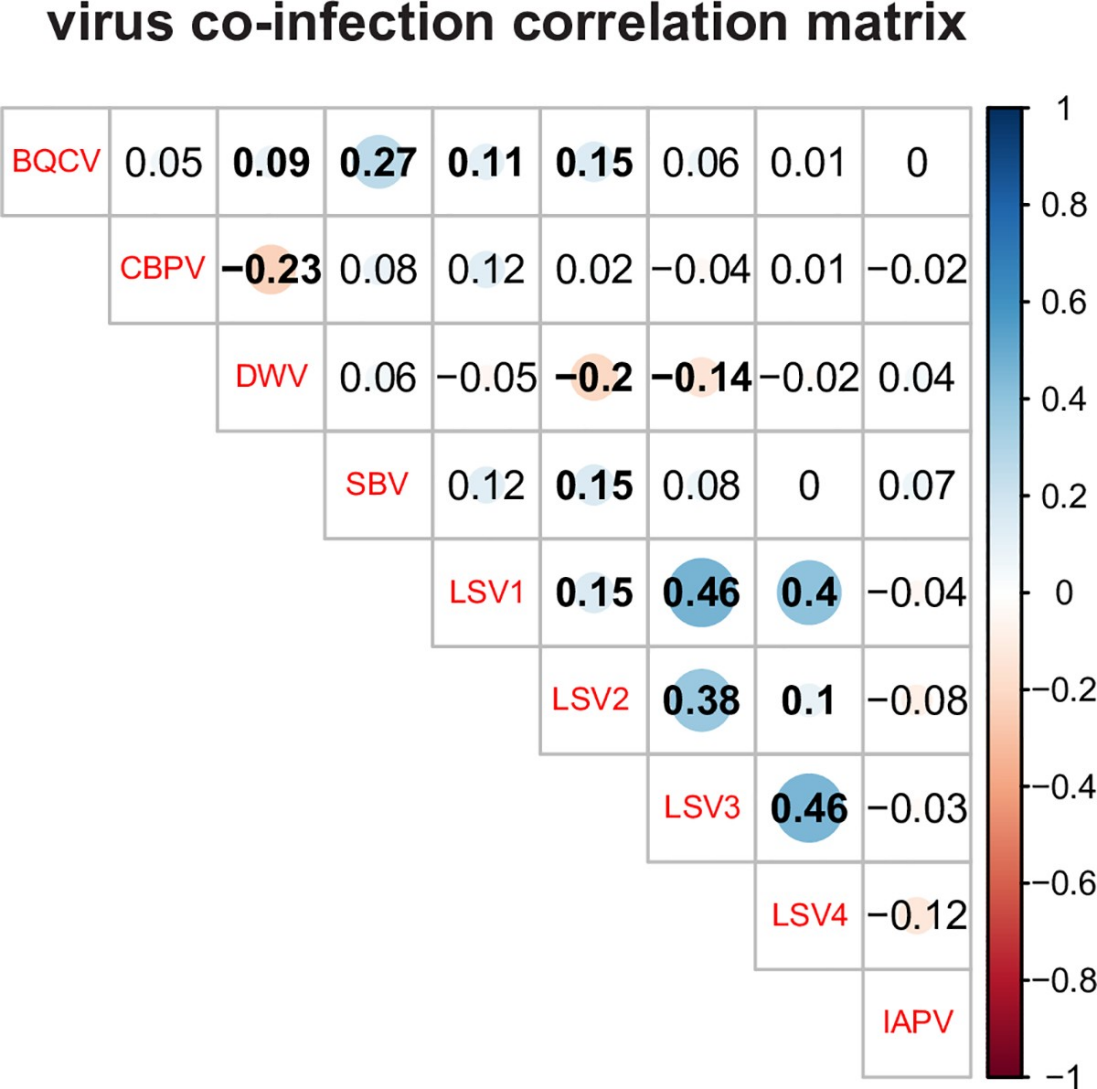
Virus co-infection correlation matrix. The abundance of viruses in co-infected colonies was analyzed by calculating the correlation coefficients for each pair-wise comparison, which are listed in each cell. Correlation coefficients (reported as *r* values) quantify the strength and direction of the changes in pathogen abundance between co-occurring pathogen pairs. The shaded red circles represent negative correlations and blue circles represent positive correlations, darker hues and larger circles indicate stronger correlations, and bold numbers indicate significant correlations (*p*-value < 0.05). In this sample cohort, the Lake Sinai viruses had the strongest positive correlations.

Honey bee colonies are often infected by multiple pathogens. Colonies in this sample cohort had up to 10 different pathogens detected at one time. To examine the pairwise interactions between the abundances of different viruses in this sample cohort, abundance values of the nine viruses assessed by qPCR (i.e., BQCV, CBPV, DWV, IAPV, LSV1, LSV2, LSV3, LSV3, and SBV) were used in a correlation analysis. The results are illustrated in a correlation matrix with correlation coefficients (R-values) that indicate the strength and direction of associations between pathogens within each sample over the course of the entire study (Fig 11 and S20 Fig). The strongest positive correlations in this sample cohort were between the Lake Sinai viruses (i.e., LSV3 strongly correlated with LSV1 and LSV4, R = 0.46). BQCV and SBV also had a significant positive correlation (R = 0.27). The strongest negative correlations were between DWV and CBPV (R = -0.23) and DWV and LSV2 (R = -0.2).

### Comparison of colonies that were dead by October

In total, 22 of the 50 monitored honey bee colonies died throughout the study. However, only four samples were obtained from dead colonies, since the other colonies died between sampling events. The majority of the colonies (i.e., 16 of 22) died between the August and October 2016 sampling dates during peak total virus and peak DWV abundance (Figs 8 and 9). Therefore, we evaluated the differences in virus abundance between colonies that lived or died by the end of the study for the August sampling date (i.e., the sample date prior to colony death by October 2016) using two-sided t-tests (S2 Table and S21 Fig). Surprisingly, neither total virus abundance nor DWV abundance in August differed in colonies that lived or died by the October sampling event. Since the greatest percentage of colonies that died in this study died between August and October, additional fall sampling events closer to the date of death may have been required to adequately assess the potential correlation between virus abundance and colony deaths.

### Results from additional sampling event–November 29, 2016

After sampling for this study was completed in October 2016, the collaborating commercial beekeeper noticed increased deaths (i.e., over 60%) in the apiary where monitored colonies were located. Therefore, additional samples were obtained (i.e., 10 samples from 9 colonies; both adult bees and brood were obtained from one colony) from weak or dead colonies on November 29, 2016. Unfortunately, samples were obtained from only two of the colonies enrolled in the initial study (i.e., colony #5 and colony #24) and therefore the data from this sample date could not be analyzed as part of the sample cohort. In addition to the panel of 13 pathogens monitored in the original study, molecular tests for two bacterial pathogens *Melissococcus plutonis* and *Paenibacillus larvae*, the causative agents of European Foulbrood Disease and American Foulbrood Disease, respectively; and a fungus that causes Chalkbrood disease *Ascosphaera apis* were carried out on these samples (S3 Table), as well as pesticide exposure analysis (USDA Gastonia Lab). All of these additional tests were negative.

Similar to the results from the monitored colonies, the most prevalent pathogens associated with this sample set were BQCV, DWV, and SBV (S3 Table). In contrast, IAPV was detected in 4 out of 10 of these samples, and it was not readily detected in other samples in this cohort or in our other studies [26, 53, 54]. Based on these results, we cannot determine if poor colony health or colony death correlated with the presence or absence of specific pathogens. However, IAPV accounting for a greater distribution of positive tests in these 10 samples compared to the monitored colonies is notable.

## Conclusion

The pathogen composition in honey bee colonies varies by date, and specific viruses vary in seasonal abundance. Together, this and previous studies underscore the importance of temporal monitoring of colonies to evaluate the impact of pathogens on colony health [39, 53, 54]. Future studies with a more uniform distribution of colony health ratings (i.e., weak, average, strong, and/or frame count data) are required to better evaluate the impact of viruses on colony health. Although, numerous studies have examined bee pathogen loads at the apiary level, few have examined the dynamics and potential correlations at the colony level [14, 23, 90]. Previous studies investigating commercially managed honey bee colonies from multiple beekeeping operations demonstrated that variance in pathogen prevalence, incidence, and abundance was partially attributed to different beekeeping operations [53, 54] Therefore, to reduce the confounding variables associated with obtaining samples from different beekeeping operations, this study followed honey bee colonies from one apiary uniformly managed by a Montana-based commercial beekeeping operation involved in almond pollination. Interestingly, analyses of this sample cohort revealed similar trends in pathogen prevalence, abundance, pathogen composition, and pairwise relationships to our previous study and others that involved multiple beekeeping operations, including the increase of DWV abundance from late summer to fall and peak LSV2 abundance in winter and/or spring [14, 53, 54, 71]. LSV2 was the only virus, of the 13 monitored in this study, for which abundance decreased with increasing frame count, confirming observations from previous studies that LSV2 abundance is associated with weaker or dead colonies [26, 53]. While LSV2 abundance decreased over time, frame count was not correlated with sample date. Therefore, the relationship between LSV2 and colony health deserves further investigation. While there are no overt symptoms associated with LSV infections, these and other virus infections are likely energetically taxing and may negatively impact honey bee health. The mean total pathogen prevalence (i.e., the average number of pathogens detected per sample out of 13 studied) in this sample cohort was highest in the sampling event just after almond pollination (April 2016), which is consistent with previous studies [53, 54]. Greater pathogen burden at this time of year also coincides with increases in honey bee brood production and foraging activities in the spring. Pathogen burden is also likely impacted by the availability and quality of nutritional resources, including both natural and supplemental food sources, though the impact of these and other factors will require further studies to evaluate. Therefore, although pathogen exposure (i.e., both pathogen incidence and abundance) varies by geographic location and the impact of viruses on honey bee colony health likely is impacted by management strategies beyond maintaining mite infestation levels below 3%, the collective results from this and other studies that involved multiple beekeeping operations suggest that seasonality (day of study) is a major driver of the pathogen abundance trends in commercially managed colonies in the US. Several studies indicate that DWV abundance coupled with high mite infestation in the fall correlate with poor colony health and overwinter losses [23, 30, 33, 66, 99]. Together, these results illustrate that longitudinal monitoring is required to obtain a complete picture of the impact of pathogens on honey bee colony population size and survival.

## Methods

### Longitudinal monitoring and sampling of honey bee colonies from one commercially managed apiary

Longitudinal monitoring of 50 honey bee (*Apis mellifera*) colonies from one Montana-based commercial beekeeping operation was conducted over the course of one year from November 2015 to October 2016. The colonies were located in the same apiary or almond grove for the duration of the study and provided the same supplemental feeding (i.e. pollen patties and sucrose syrup). Samples were collected at six discrete time points, including two while colonies were located in California during (i.e., March 10, 2016) and just after (i.e., April 23, 2016) almond pollination (S1 Table). Colony population size, which served as a proxy for colony health, was estimated by counting the number of frames that were greater than 2/3 covered with honey bees [93]. For some analyses, frame count data were categorized into the following three colony health ratings: weak ≤ 5, average = 6–8, strong ≥ 9 frames. For the second sampling event on March 10, 2016 while colonies were involved in almond population, all colonies were described as ‘strong’ by the bee biologist at the almond company, but we did not receive frame count data. Therefore, all colonies were assigned an estimated average frame count value of 15 for analyses. By the end of the study in October 2016, each colony was sampled between three and six times. In total, 262 samples were analyzed, of which 37 were categorized as weak, 24 as average, 197 as strong, and 4 were dead at the time of sampling. Over the course of the study 22 colonies died, but samples were not always obtained at the time of death. During the last sampling event in October 2016, 28 colonies were alive and four dead colonies were sampled (n = 32). *Varroa destructor* mite infestation levels were managed with the goal of maintaining levels below the treatment threshold of 3% infestation. Specific treatments included thymol (Apiguard®) in October 2015, amitraz (Apivar®) in December 2015, formic acid in May 2016, and thymol (Apiguard®) in October 2016.

### Honey bee samples

At each sampling event, live sterile female worker honey bees (~ 150 per sample) of mixed age were obtained from a frame containing developing bees (brood) located within the center of each colony. Collected honey bee samples were placed on ice or dry ice in the field, stored at -20°C, transported on dry ice, and transferred to -80°C for storage prior to analysis. Ten female bees from each sample were used for RNA extraction, cDNA synthesis, pathogen-specific PCR, and qPCR. Based on empirical data, literature values, and practical sample handling considerations, we assayed ten bees per colony per sampling event [26, 39, 53, 54]. The following equation from Pirk et al. 2013, N = ln(1-D) / ln(1-P) (N = sample size, ln = natural logarithm, D = probability of detection, P = proportion of infected bees) predicts that with a sample size of ten bees, pathogenic infections affecting 26% or more of the individuals within a colony would be detected with 95% probability [105]; this sample size has proven sufficient for the pathogen-specific PCR detection of highly prevalent pathogens [26, 53, 105]. We reasoned that if 10 bees are sufficient to estimate pathogen prevalence then we could also use this same sample size to examine trends in virus abundance. Assessment of the number of bees that best represents quantitative virus load in a colony may be a parameter worth further exploring in future studies, although 10 bees per colony is in-line with other studies including apiary level studies of virus prevalence including The German Bee Monitoring Project (i.e., average of 1 bee per colony, since 10 bees were analyzed from composite samples obtained from 10 colonies per apiary) [23], the Canadian National Honey Bee Health Survey (i.e., 6 bees per colony, since a composite of 60 bees obtained from 10 colonies per apiary) [90], and virus abundance from USA Bee Informed Partnership (i.e., average 6.25 bees per colony, as virus abundance was assessed at the apiary level based composite sample of 50 bees from 8 colonies per apiary) [14]. Likewise, field evaluation of virus abundance in foraging bees was done by pooling 10 honey bees per field sampling site [106]. Several other colony level studies have also used 5 or 10 bees per colony per sample date to examine trends in virus abundance at the colony level [26, 53, 54, 71]. Assessment of virus abundance individual bees (i.e., 9 bees per colony from three colonies in three apiaries) made observations similar to those described herein (i.e., greater abundance of DWV in the fall/winter months, detection of BQCV throughout the study with pretty consistent levels with higher abundance in summer (June and July), a short window of CBPV in May and June) [74].

### RNA isolation

Bee samples, five bees each in two 2 mL Eppendorf tubes (10 bees total per sample), were homogenized in 800 μl water using metal beads (3 mm) and a TissueLyzer (Qiagen) at 30 Hz for 2 minutes. Samples were centrifuged for 12 minutes at 12,000 x g at 4°C to pellet debris, supernatants from two tubes per each sample were combined (~ 700 ul total), and the RNA was extracted using Trizol reagent (Life Technologies) according to the manufacturer’s instructions and quantified using a NanoDrop™ spectrophotometer (ThermoFisher).

### Reverse transcription/cDNA synthesis

Complimentary DNA (cDNA) synthesis reactions were performed by incubating 2 μg of total RNA extracted from each honey bee sample, 200 units of Moloney murine leukemia virus (M-MLV) reverse transcriptase (Promega), and 500 ng random hexamer primers (Integrated DNA Technologies) in a 25 μl reaction for 1 hour at 37°C, according to the manufacturer’s instructions. cDNA was diluted 1:2 in dH20 (50 μl total), resulting in a concentration of 40 ng/μl of relative RNA copies.

### Polymerase Chain Reaction (PCR)

Pathogen specific PCR was performed according to standard methods using the primers listed in S5 Table. Specifically, PCR was used to determine the prevalence of 13 different pathogens: ABPV, BQCV, CBPV, DWV IAPV, KBV, LSV1, LSV2, LSV3, LSV4, SBV, *N*. *ceranae*, and *L*. *passim* (S1 Table). In brief, each PCR included 2 μl cDNA template (80 ng total RNA per reaction), combined with 10 pmol each of forward and reverse pathogen specific primers, and amplified with 0.5 μl (5 U/μl) ChoiceTaq polymerase (Thomas Scientific) according to the manufacturer’s instructions using the following cycling conditions: 95°C for 5 minutes; 35 cycles of 95°C for 30 seconds, 55°C for 30 seconds, and 72°C for 30 seconds; followed by final elongation at 72°C for 4 minutes. The PCR products were visualized using 1.5% agarose gel electrophoresis followed by fluorescence imaging; a subset of the PCR products were confirmed by Sanger sequencing. Positive and negative control reactions were included for all pathogen-specific PCR analyses and exhibited the expected results. In addition, PCR of a host encoded gene, *Apis m*. *rpl8*, was performed using 2 μL cDNA template on each sample to ensure cDNA quality.

### Quantitative Polymerase Chain Reaction (qPCR)

Quantitative PCR (qPCR) was used to determine the relative pathogen abundance of the most prevalent pathogens. Specifically, qPCR was used to determine the abundance of BQCV, CBPV, DWV, IAPV, LSV1, LSV2, LSV3, LSV4, and SBV (S5 Table). All qPCR reactions were performed in triplicate using a CFX Connect Real Time instrument (BioRad) and the following reaction conditions: 2 μL of cDNA (80 ng total RNA per reaction) template in 20 μL reactions containing 1X ChoiceTaq Mastermix (Thomas Scientific), 0.4 μM each forward and reverse primer, 1X SYBR Green (Life Technologies), and 3 mM MgCl_2_. The qPCR thermo-profile consisted of a single pre-incubation 95°C (1 minute), 40 cycles of 95°C (10 seconds), 60°C (20 seconds), and 72°C (15 seconds). Plasmid standards, containing from 10^9^ to 10^3^ copies per reaction, were used as qPCR templates to assess primer efficiency and quantify the relative abundance of each pathogen. The linear standard equation for primer efficiency of each primer set, which was generated by plotting the crossing point (Cp) versus the log_10_ of the initial plasmid copy number, is as follows: BQCV: y = -3.4245x + 39.803, R^2^ = 0.997; CBPV: y = -3.8511x + 44.88, R^2^ = 0.9988; DWV: y = -3.9236x + 44.475, R^2^ = 0.9823; LSV1: y = -3.7177x + 41.631, R^2^ = 0.9908; LSV2: y = -3.3648x + 41.736, R^2^ = 0.9886; LSV3: y = -3.6719x + 42.23, R^2^ = 0.9955; LSV4: y = -3.7461x + 43.694, R^2^ = 0.998; SBV: y = -3.7378x + 44.037, R^2^ = 0.9921. The minimum qPCR detection levels were ≤ 1,000 copies per sample.

### Statistical analysis of pathogen prevalence

Pathogen prevalence for each sample was defined as the sum of 13 honey bee pathogens (i.e., ABPV, BQCV, SBV, LSV 1–4, KBV, IAPV, ABPV, CBPV, *L*. *passim*, and *N*. *ceranae*) detected using conventional Polymerase Chain Reaction (PCR).The mean pathogen prevalence of samples was compared at different time points during the study and in samples from different colony health ratings using a Generalized linear mixed effects model (GLMM) with a Poisson family distribution, main effects for sampling time point and colony health rating, and a random effect for colony to account for repeated samples on the same colony. A model with a random slope for each colony was considered but performed worse than a model fitting a random intercept according to Akaike’s Information Criteria (AIC). Additionally, sampling time point and colony health rating were included as separate fixed effects in each model because there was no statistical evidence of a correlation between mean frame count and sampling time point and were therefore assumed to be independent. Differences in pathogen prevalence between colony strength ratings and sampling periods were described as a multiplicative change on the log-scale. Pairwise contrasts between strength ratings and sampling time points were conducted after applying a Tukey’s family-wise confidence interval correction to p-values to account for multiple tests and a potential inflated Type 1 error-rate.

### Statistical analysis of qPCR

Quantitative Polymerase Chain Reaction (qPCR) assays of nine viruses (BQCV, CBPV, DWV, IAPV, LSV1, LSV2, LSV3, LSV4, and SBV) resulted in a long right tailed distribution ranging from 0 to 700,763,417 viral copies from honey bee samples. To normalize the response distribution, a log+1 transformation was applied to virus abundance resulting in a distribution of response values that were zero if the pathogen was below detection threshold or a normal distribution of continuous values if the pathogen was above a detection threshold. Consequently, the distribution of response values resembled a zero-inflated normal distribution. Therefore, we employed a two-step process to model the virus prevalence and abundance to account for the non-normal distribution of the response variable.

First to model virus prevalence data, virus abundance was collapsed into a binary response to account for frequent zeros in the response distribution. Virus abundance for each sample was assigned a value of “1” if a virus was present or a value of “0” if a virus was absent based on qPCR assays. A GLMM with a binomial family distribution and random effect for individual colony was used to estimate the odds that a virus would be detected in a colony in response to the day of the study and colony strength rating while accounting for resampling colonies [94]. The estimated log-odds of coefficients were exponentiated in order to back-transform these estimates into the odds of detecting a virus. The estimated odds from these models represent the chances that a pathogen would be present in a colony relative to the chances that a pathogen is not present. Odds were estimated for both colony health rating and day of study. A back-transformed coefficient greater than one indicates increased odds of colony-level pathogen detection. The odds that a pathogen would be present was plotted in response to the day of the study as a percentage.

Second, on a per virus basis, samples without detectable virus were discarded from analysis to reduce the frequent presence of zeros in the response distribution, resulting in a normal, continuous distribution of virus abundance response values. For samples with pathogens detected using qPCR, generalized additive mixed models (GAMM) were used to visualize the non-linear rate of change of pathogen abundance in response to the day of study and colony health rating, while also accounting for resampling colonies. Smoothing terms were estimated for day of study and colony health rating and a random effect for colony ID was included to account for the effects of resampling pathogen abundance on the same colonies through time. Smoothing parameters for GAMMs were determined by setting *k* to the highest value allowed by the data to minimize residual deviance but also to avoid undersmoothing and missing relevant trends. Estimated degrees of freedom (edf) are reported to reflect the complexity of curves estimated by the GAMM. As a result, GAMMs were able to reflect large changes in pathogen abundance at different points throughout the year without obscuring small changes. For example, when the edf = 1 for a smoother, it is estimating a linear trend. Generalized additive mixed models (GAMM) were built using the r-package gamm4 [107].

Derivative functions with 95% confidence intervals were determined for each GAMM to identify periods during the sampling timeframe when the rate of change for pathogen abundance was significantly different from zero [108]. Specifically, the first derivative of the fitted spline was calculated to identify the rate of change of virus abundance throughout the timeframe and 95% confidence intervals were built around the first derivative to distinguish periods of time when the change in virus abundance is significantly different from zero. Derivatives were constructed using the Deriv() function and confint.Deriv() function [109].

### Power analysis

The unequal distribution of colony health ratings in this sample cohort was not ideal for statistical analyses, therefore, to benefit future studies aimed at investigating the relationship between colony health and pathogen abundance we performed retrospective power analysis using a one-way ANOVA. The number of samples per categorical health rating was estimated after including the number of groups, between group variation, within group variation, the power to detect false negatives, and the level of significance, within the power analysis. The number of groups corresponded to the number of colony health ratings (n = 4; dead, weak, average, and strong). The between group variance was estimated as the variance in mean pathogen abundance between the four groups of colony health ratings. Within group variance is assumed equal for each group and estimated as the mean of the variance in pathogen abundance from each colony health rating. The power of the test was set to 80% and the significance level was set at 0.05. The power analysis was conducted in the ‘stats’ package in R [110]. Although a one-way ANOVA analysis cannot be carried out on this data set due to repeated sampling from monitored colonies, which violates the assumption of independent samples, this test was used to provide an estimate of the number of samples required from each categorical health rating to detect significant differences in total virus abundance. This analysis estimated that 210 samples for each categorical health rating would be required to detect a significant difference (0.05 significance level) in total pathogen abundance. Increasing and balancing the sample sizes of colonies in each colony strength rating at the onset of monitoring would be ideal for detecting broadscale patterns between colony health ratings and pathogen abundance, although in practice commercial beekeepers manage their operations to limit the number of weak colonies [53, 54, 93].

### Community analysis

Virus community composition was defined as the log+1 transformed abundance of all viruses assayed using qPCR (i.e., BQCV, CBPV, DWV, IAPV, LSV1, LSV2, LSV3, LSV4, and SBV) for each sample relative to all other samples in the study, including those with a zero value (i.e., virus level below detection). A Bray-Curtis dissimilarity index quantified the relative dissimilarity of the abundance of all viruses for each sample relative to all other samples. Virus community dissimilarity for each sample relative to all other samples was visualized using a non-metric multi-dimensional scaling (NMDS) plot. Samples were categorized based on discrete sampling events (November 2015, March 2016, April 2016, June 2016, August 2016, October 2016) to visualize the relative differences in virus community composition throughout the study.

A permutational analysis of variance (PERMANOVA) was used to test whether the community composition of viruses was influenced by the main and interactive effects of sampling time point and colony health rating [111]. Additionally, the average distance of the pathogen community to the group (sampling time point) centroid for each sample was calculated using a homogeneity of variance test (betadisper) to compare the variability of virus community composition at different sampling time points. The mean distance-to-centroid for samples within the same sampling time point was compared between sampling time points using an ANOVA. Significant differences between sampling time points (alpha <0.05) were followed by a Tukey’s HSD *post-hoc* test to identify pairwise differences in virus community composition between sampling time points. Pairwise differences in virus community variability were significantly different when 95% confidence intervals (CI) were not overlapping. Furthermore, a similarity-percentage (SIMPER) analysis was performed to identify individual viruses contributing to the most dissimilarity in virus community composition between sampling time periods.

### Pairwise pathogen interactions

The relationships between the abundances of co-infecting viruses were evaluated by pairwise analyses of qPCR data from all samples that had both viruses in each pair. The strength and direction of changes in abundance between co-infecting viruses (BQCV, CBPV, DWV, IAPV, LSV1, LSV2, LSV3, LSV4, and SBV) were estimated using correlation coefficients. Results were visualized on a correlation matrix using the ‘corrplot’ package in R [112]. Shaded red circles represent negative correlations, while shaded blue circles represent positive correlations among pairwise virus abundances. Larger and darker circles represent stronger correlations between viruses. Significant correlations (p-value < 0.05) are bolded.

## Supporting information

S1 Fig Distribution of honey bee pathogens detected in monitored colonies. Distribution of honey bee pathogens detected in monitored colonies over the entire study. Pathogen specific PCR was used to test honey bee samples (n = 262) for 13 commonly occurring pathogens (i.e., ABPV, BQCV, CBPV, DWV, IAPV, KBV, LSV1, LSV2, LSV3, LSV4, SBV, *Nos*., and *C*.*m*.*/L*.*p*.) (S1 Table). The distribution of each pathogen is shown as a percentage of all positive tests (n = 1199) and by the percentage of positive tests for each colony health rating (i.e., weak (n = 172), average (n = 127), and strong (n = 889). The results from dead colonies (n = 4, 11 positive tests) were not graphed as a separate column but are included in all positive tests. Pathogens with the greatest distribution were BQCV (18%), SBV (14%), DWV (13%) and *C*.*m*.*/L*.*p*. (13%), followed by LSV2 (11%), LSV1 (8%), LSV3 (6%), LSV4 (5%), CBPV (5%), *Nos*. (5%), IAPV (1%), KBV (1%), and ABPV (0.3%).
(PDF)

S2 Fig Pathogen incidence in honey bee colonies throughout the study for all monitored pathogens. The observed incidence of all the pathogens monitored in this study (i.e., BQCV, DWV, SBV, *C*.*m*./*L*.*p*., *Nos*., LSV1, LSV2, LSV3, LSV4, CBPV, IAPV, ABPV, and KBV) are represented as a percentage of all samples at each time point (dark gray = positive).
(PDF)

S3 Fig Analysis of mean total pathogen prevalence by colony health rating. Pathogen prevalence did not vary by colony health rating in this sample cohort. Honey bee samples obtained from dead, weak, average, and strong colonies were tested for the presence of 13 pathogens (i.e., ABPV, BQCV, CBPV, DWV, IAPV, KBV, LSV1, LSV2, LSV3, LSV4, SBV, *L*. *passim*, and *N*. *ceranae*) using pathogen specific PCR. Total pathogen prevalence refers to the sum of the different pathogens detected in each sample. The mean of the total number of pathogens per colony strength rating, the standard error estimate of the mean, and the number of colonies per colony health rating within this cohort are presented in the table.
(PDF)

S4 Fig Percent odds of detecting SBV over the course of the study. The odds of detecting SBV increased by 0.005886 (SE +/- 0.0024) with each day of the study (Z-test, z-value = 2.491, p-value = 0.0127). Colony level SBV data for each sample date is represented as colony strength indicating icons (i.e., strong = yellow square, average = green circle, weak = blue triangle, and dead = black diamond). The best fit line (blue) of the odds of detecting SBV in response to day of study was determined with a generalized linear mixed effect model (GLMM) with a binomial family distribution and random effect for individual colony, and is surrounded by upper and lower standard error estimates (gray).
(PDF)

S5 Fig Percent odds of detecting CBPV over the course of the study. The odds of detecting CBPV increased by 0.00345 (SE +/- 0.001) with each day of the study (Z-test, z-value = 2.211, p-value = 0.027). Colony level CBPV data for each sample date is represented as colony strength indicating icons (i.e., strong = yellow square, average = green circle, weak = blue triangle, and dead = black diamond). The best fit line (blue) of the odds of detecting CBPV in response to day of study was determined with a generalized linear mixed effect model (GLMM) with a binomial family distribution and random effect for individual colony, and is surrounded by upper and lower standard error estimates (gray).
(PDF)

S6 Fig Percent odds of detecting IAPV over the course of the study. The odds of detecting IAPV increased by 0.005 (SE +/- 0.001918) with each of day of the study (z-value = 2.904, p-value = 0.004). Colony level IAPV data for each sample date is represented as colony strength indicating icons (i.e., strong = yellow square, average = green circle, weak = blue triangle, and dead = black diamond). The best fit line (blue) of the odds of detecting IAPV in response to day of study was determined with a generalized linear mixed effect model (GLMM) with a binomial family distribution and random effect for individual colony, and is surrounded by upper and lower standard error estimates (gray).
(PDF)

S7 Fig Percent odds of detecting BQCV over the course of the study. The odds of detecting BQCV increased by 0.003 (SE +/- 0.001) with each day of the study (Z-test, z-value = 2.536, p-value = 0.0112). Colony level BQCV data for each sample date is represented as colony strength indicating icons (i.e., strong = yellow square, average = green circle, weak = blue triangle, and dead = black diamond). The best fit line (blue) of the odds of detecting BQCV in response to day of study was determined with a generalized linear mixed effect model (GLMM) with a binomial family distribution and random effect for individual colony, and is surrounded by upper and lower standard error estimates (gray).
(PDF)

S8 Fig Percent odds of detecting LSV2 over the course of the study. The odds of detecting LSV2 decreased by 0.0037 (SE +/- 0.0012) per day (Z-test, z-value = -3.098, p-value = 0.00795). Colony level LSV2 data for each sample date is represented as colony strength indicating icons (i.e., strong = yellow square, average = green circle, weak = blue triangle, and dead = black diamond). The best fit line (blue) of the odds of detecting LSV2 in response to day of study was determined with a generalized linear mixed effect model (GLMM) with a binomial family distribution and random effect for individual colony and is surrounded by upper and lower standard error estimates (gray).
(PDF)

S9 Fig Percent odds of detecting LSV1 over the course of the study. The odds of detecting LSV1 did not vary over the course of the study. LSV1 abundance was collapsed into a binary response to account for frequent zeros in the response distribution. Colony level LSV1 data for each sample date is represented as colony strength indicating icons (i.e., strong = yellow square, average = green circle, weak = blue triangle, and dead = black diamond). The best fit line (blue) of the odds of detecting LSV1 in response to day of study was determined with a generalized linear mixed effect model (GLMM) with a binomial family distribution and random effect for individual colony and is surrounded by upper and lower standard error estimates (gray).
(PDF)

S10 Fig Percent odds of detecting LSV3 over the course of the study. The odds of detecting LSV3 did not vary over the course of the study. Colony level LSV3 data for each sample date is represented as colony strength indicating icons (i.e., strong = yellow square, average = green circle, weak = blue triangle, and dead = black diamond). The best fit line (blue) of the odds of detecting LSV3 in response to day of study was determined with a generalized linear mixed effect model (GLMM) with a binomial family distribution and random effect for individual colony, and is surrounded by upper and lower standard error estimates (gray).
(PDF)

S11 Fig Percent odds of detecting LSV4 over the course of the study. The odds of detecting LSV4 did not vary over the course of the study. Colony level LSV4 data for each sample date is represented as colony strength indicating icons (i.e., strong = yellow square, average = green circle, weak = blue triangle, and dead = black diamond). The best fit line (blue) of the odds of detecting LSV4 in response to day of study was determined with a generalized linear mixed effect model (GLMM) with a binomial family distribution and random effect for individual colony, and is surrounded by upper and lower standard error estimates (gray).
(PDF)

S12 Fig Changes in LSV2 abundance in honey bee colonies throughout the study. A. LSV2 abundance varied over time and was lowest at the end of the study (day of study_edf =_ 4.281, p-value = 0.0001). The natural log transformed LSV2 abundance data as determined by qPCR in honey bee samples were plotted by day of study. The best fit line (blue) for LSV2 mixed model (GAMM) and is surrounded by upper and lower standard error estimates (gray). Colony level LSV2 data for each sample date is represented as colony strength indicating icons (i.e., strong = yellow square, average = green circle, weak = blue triangle, and dead = black diamond) with unique colony identifier numbers, which illustrate the changes in virus abundance of individual colonies throughout the study. B. The first derivative of the fitted spline in A was calculated to identify the rate of change of LSV2 abundance throughout the timeframe and 95% confidence intervals (gray) were built around the first derivative to distinguish periods of time when the change in virus abundance is significantly different from zero. LSV2 abundance significantly increased (blue) from 0 to 100 days of the study and significantly decreased (red) from 100 to 175 days and from 300 to 350 days of the study.
(PDF)

S13 Fig Changes in LSV4 RNA abundance in honey bee colonies throughout the study. A. LSV4 abundance varied over the course of the study (day of study_edf =_ 4.071, p-value = 0.000122). The natural log transformed LSV4 abundance data as determined by qPCR (y-axis) in honey bee samples were plotted by day of study (x-axis). The best fit line (blue) for LSV4 mixed model (GAMM) and is surrounded by upper and lower standard error estimates (gray). Colony level LSV4 data for each sample date is represented as colony strength indicating icons (i.e., strong = yellow square, average = green circle, weak = blue triangle, and dead = black diamond) with unique colony identifier numbers, which illustrate the changes in virus abundance of individual colonies throughout the study. B. The first derivative of the fitted spline in panel A was calculated to identify the rate of change of LSV4 abundance throughout the timeframe and 95% confidence intervals (gray) were built around the first derivative to distinguish periods of time when the change in virus abundance is significantly different from zero. LSV4 abundance significantly increased (blue) from 175 to 225 days and decreased (red) from 275 to 325 days of the study.
(PDF)

S14 Fig Changes in SBV abundance in honey bee colonies throughout the study. A. SBV abundance varied over the course of the study (day of study_edf_ = 2.252, p-value = 0.00348). The natural log transformed SBV abundance data as determined by qPCR (y-axis) in honey bee samples were plotted by day of study (x-axis). The best fit line (blue) for SBV mixed model (GAMM) and is surrounded by upper and lower standard error estimates (gray). Colony level SBV data for each sample date is represented as colony strength indicating icons (i.e., strong = yellow square, average = green circle, weak = blue triangle, and dead = black diamond) with unique colony identifier numbers, which illustrate the changes in virus abundance of individual colonies throughout the study. B. The first derivative of the fitted spline in panel A was calculated to identify the rate of change of SBV abundance throughout the timeframe and 95% confidence intervals (gray) were built around the first derivative to distinguish periods of time when the change in virus abundance is significantly different from zero. SBV abundance significantly increased (blue) from 0 to 100 days of the study, then did not vary for the remainder of the study.
(PDF)

S15 Fig Changes in IAPV abundance in honey bee colonies throughout the study. A. IAPV abundance decreased linearly over the course of the study (day of study_edf_ = 1, p-value = 0.0274). The natural log transformed IAPV abundance data as determined by qPCR (y-axis) in honey bee samples were plotted by day of study (x-axis). The best fit line (blue) for IAPV mixed model (GAMM) and is surrounded by upper and lower standard error estimates (gray). Colony level IAPV data for each sample date is represented as colony strength indicating icons (i.e., strong = yellow square, average = green circle, weak = blue triangle, and dead = black diamond) with unique colony identifier numbers, which illustrate the changes in virus abundance of individual colonies throughout the study. B. The first derivative of the fitted spline in panel A was calculated to identify the rate of change of IAPV abundance throughout the timeframe and 95% confidence intervals (gray) were built around the first derivative to distinguish periods of time when the change in virus abundance is significantly different from zero. There were no periods of significant change in the derivative.
(PDF)

S16 Fig Changes in BQCV abundance in honey bee colonies throughout the study. A. BQCV did not vary over the course of the study (day of study_edf_ = 1.024, p-value = 0.9107). The natural log transformed BQCV abundance data as determined by qPCR (y-axis) in honey bee samples were plotted by day of study (x-axis). The best fit line (blue) for BQCV mixed model (GAMM) and is surrounded by upper and lower standard error estimates (gray). Colony level BQCV data for each sample date is represented as colony strength indicating icons (i.e., strong = yellow square, average = green circle, weak = blue triangle, and dead = black diamond) with unique colony identifier numbers, which illustrate the changes in virus abundance of individual colonies throughout the study. B. The first derivative of the fitted spline in panel A was calculated to identify the rate of change of BQCV abundance throughout the timeframe and 95% confidence intervals (gray) were built around the first derivative to distinguish periods of time when the change in virus abundance is significantly different from zero. There were no periods of significant change in the derivative.
(PDF)

S17 Fig Changes in LSV1 abundance in honey bee colonies throughout the study. A. LSV1 did not vary over the course of the study (day of study_edf_ = 1.428, p-value = 0.286). The natural log transformed LSV1 abundance data as determined by qPCR (y-axis) in honey bee samples were plotted by day of study (x-axis). The best fit line (blue) for LSV1 mixed model (GAMM) and is surrounded by upper and lower standard error estimates (gray). Colony level LSV1 data for each sample date is represented as colony strength indicating icons (i.e., strong = yellow square, average = green circle, weak = blue triangle, and dead = black diamond) with unique colony identifier numbers, which illustrate the changes in virus abundance of individual colonies throughout the study. B. The first derivative of the fitted spline in panel A was calculated to identify the rate of change of LSV1 abundance throughout the timeframe and 95% confidence intervals (gray) were built around the first derivative to distinguish periods of time when the change in virus abundance is significantly different from zero. There were no periods of significant change in the derivative.
(PDF)

S18 Fig Changes in LSV3 abundance in honey bee colonies throughout the study. A. LSV3 did not vary over the course of the study (day of study_edf_ = 1, p-value = 0.420). The natural log transformed LSV3 abundance data as determined by qPCR (y-axis) in honey bee samples were plotted by day of study (x-axis). The best fit line (blue) for LSV3 mixed model (GAMM) and is surrounded by upper and lower standard error estimates (gray). Colony level LSV3 data for each sample date is represented as colony strength indicating icons (i.e., strong = yellow square, average = green circle, weak = blue triangle, and dead = black diamond) with unique colony identifier numbers, which illustrate the changes in virus abundance of individual colonies throughout the study. B. The first derivative of the fitted spline in panel A was calculated to identify the rate of change of LSV1 abundance throughout the timeframe and 95% confidence intervals (gray) were built around the first derivative to distinguish periods of time when the change in virus abundance is significantly different from zero. There were no periods of significant change in the derivative.
(PDF)

S19 Fig Relative virus composition of honey bee colonies by colony health. Pathogen community composition did not vary by colony health (PERMANOVA, F_3,255_ = 1.35, p-value = 0.191). The virus community composition for each sample (i.e., the log natural transformed abundance of BQCV, CBPV, DWV, SBV, LSV1, LSV2, LSV3, LSV4, and IAPV, as assessed by qPCR) was compared in relation to colony health rating (i.e., weak, average, and strong). The position of each point indicates the virus composition of each sample relative to all other samples (i.e., samples with more similar virus composition are closer), calculated using a Bray-Curtis dissimilarity index and plotted on a non-metric multidimensional scaling (NMDS) plot.
(PDF)

S20 Fig Virus co-infection correlation matrix p-values. The abundance of viruses in co-infected colonies was analyzed by calculating the correlation coefficients for each pair-wise comparison, which were reported as *r* values in Fig 9, the corresponding p-values are listed in each cell, bold numbers indicate significant correlations (*p*-value < 0.05). The shaded red circles represent negative correlations and blue circles represent positive correlations; darker hues and larger circles indicate stronger correlations.
(PDF)

S21 Fig August comparison of virus burden in honey bee colonies that died versus honey bee colonies that survived. In total, 22 of the 50 monitored honey bee colonies died throughout the study, although samples were obtained for only 4 of those samples when they were dead. The majority of the colonies (i.e., 16 of 22) died between the August and October 2016 sampling dates. Therefore, individual and total viral abundance data from the August 2016 sample date were evaluated using a two-sided t-test to assess potential differences between colonies that were alive or dead in October 2016. These comparisons were carried out between colonies that tested positive for the viruses and therefore only comparisons with sufficient sample size (i.e., total abundance, DWV, SBV, and BQCV) are reported. This analysis indicated that there were no differences in virus abundance in the August sample date between colonies that were alive or dead by the October 2016 sampling date.
(PDF)

S1 Table Complete data set. Longitudinal monitoring of 50 honey bee (*Apis mellifera*) colonies from one Montana-based commercial beekeeping operation was conducted over the course of one year from November 2015 to October 2016. Samples were collected at six discrete time points, including two while colonies were located in California during (i.e. March 10, 2016) and just after almond pollination (i.e., April 23, 2016). By the end of the study in October 2016, colonies were sampled between three and six times. In total, we analyzed 262 samples, of which 37 were categorized as weak, 24 as average, 197 as strong, 4 were dead at the time of sampling. Honey bee colony population size was used as a proxy for colony health by counting the number of frames more than 2/3 covered with bees (i.e. weak (5 or fewer frames), average (6–8 frames), strong (9 or more frames) covered with bees)). Pathogen diagnostics was performed by PCR (1 = positive detection, 0 = not detected, NA—not assessed or no sample) for 13 pathogens and the most prevalent viral pathogens were assessed by qPCR. Relative RNA equivalents were determined by qPCR and were natural log transformed for statistical analyses (NA = not assessed by qPCR). Samples for which there was no virus detected by PCR would be below detection by qPCR and were thus considered as 0 during statistical analyses of qPCR. Over the course of the study, 22 colonies died, only four of which were collected as dead and analyzed (October 2016). The 18 other colonies that died throughout the study are included in the table at the time period they were found dead, then ommited from the table thereafter.
(XLSX)

S2 Table In total, 22 of the 50 monitored honey bee colonies died throughout the study, although samples were obtained for only 4 of those samples when they were dead. The majority of the colonies (i.e., 16 of 22) died between the August and October 2016 sampling dates. Therefore, we evaluated the differences in virus abundance and pathogen composition between colonies that lived or died by the end of the study for the August sampling date (i.e., the sample date prior to colony death by October 2016). Colonies that died previous to the August 2016 time point are not included in this table. The 'October status' column indicates whether the colony was alive or dead by the next sampling time point in October 2016.
(XLSX)

S3 Table After sampling for this study was completed in October 2016, the collaborating commercial beekeeper noticed increased deaths (over 60%) in the apiary where monitored colonies were located. Therefore, additional samples were obtained (i.e., 10 samples from 9 colonies; both adult bees and brood were obtained from one colony) from weak or dead colonies on November, 2016. Only two of the colonies sampled were established monitor colonies (i.e., c5 and c24), and therefore this data was not analyzed with the sample cohort. In addition to the panel of 13 pathogens monitored in the original study, additional molecular tests for two bacterial pathogens *Melissococcus plutonis* and *Paenibacillus larvae*, the causative agents of European Foulbrood Disease and American Foulbrood Disease, respectively; and a fungus that cause Chalkbrood disease Ascosphaera apis were carried out on these samples.
(XLSX)

S4 Table Similarity percentage analysis of the relative pathogen composition between sampling events. The percent difference attributed to each pathogen between consecutive sampling events was calculated using a similarity percentage (SIMPER) analysis of a Bray-Curtis dissimilarity matrix. The cumulative difference of the top three pathogens in comparisons between consecutive sampling events is reported as a percentage.
(DOCX)

S5 Table Primers used in this study. (DOCX)
