# Supplementary figures and images for: Longitudinal monitoring of honey bee colonies reveals dynamic nature of virus abundance and indicates a negative impact of Lake Sinai virus 2 on colony health
Source: PLoS One. 2020 Sep 8;15(9):e0237544. doi: 10.1371/journal.pone.0237544 (PMC7478651; doi:10.1371/journal.pone.0237544)

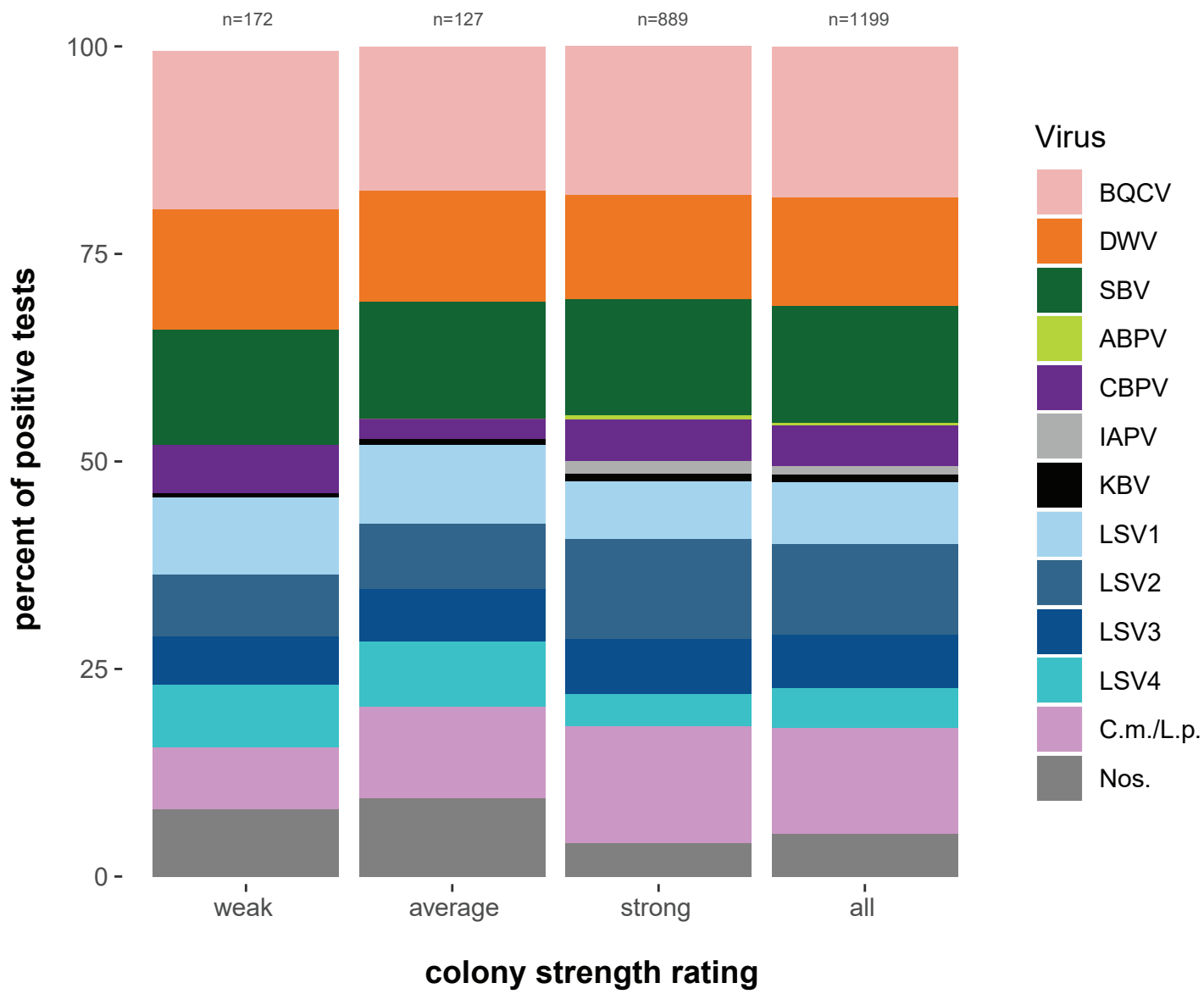

**Supporting Figure S1: Pathogen distribution by colony health rating.**

Supplement: S1 Fig — Distribution of honey bee pathogens detected in monitored colonies over the entire study. Pathogen specific PCR was used to test honey bee samples (n = 262) for 13 commonly occurring pathogens (i.e., ABPV, BQCV, CBPV, DWV, IAPV, KBV, LSV1, LSV2, LSV3, LSV4, SBV, Nos., and C.m./L.p.) (S1 Table). The distribution of each pathogen is shown as a percentage of all positive tests (n = 1199) and by the percentage of positive tests for each colony health rating (i.e., weak (n = 172), average (n = 127), and strong (n = 889). The results from dead colonies (n = 4, 11 positive tests) were not graphed as a separate column but are included in all positive tests. Pathogens with the greatest distribution were BQCV (18%), SBV (14%), DWV (13%) and C.m./L.p. (13%), followed by LSV2 (11%), LSV1 (8%), LSV3 (6%), LSV4 (5%), CBPV (5%), Nos. (5%), IAPV (1%), KBV (1%), and ABPV (0.3%). (PDF) [file pone.0237544.s001.pdf]

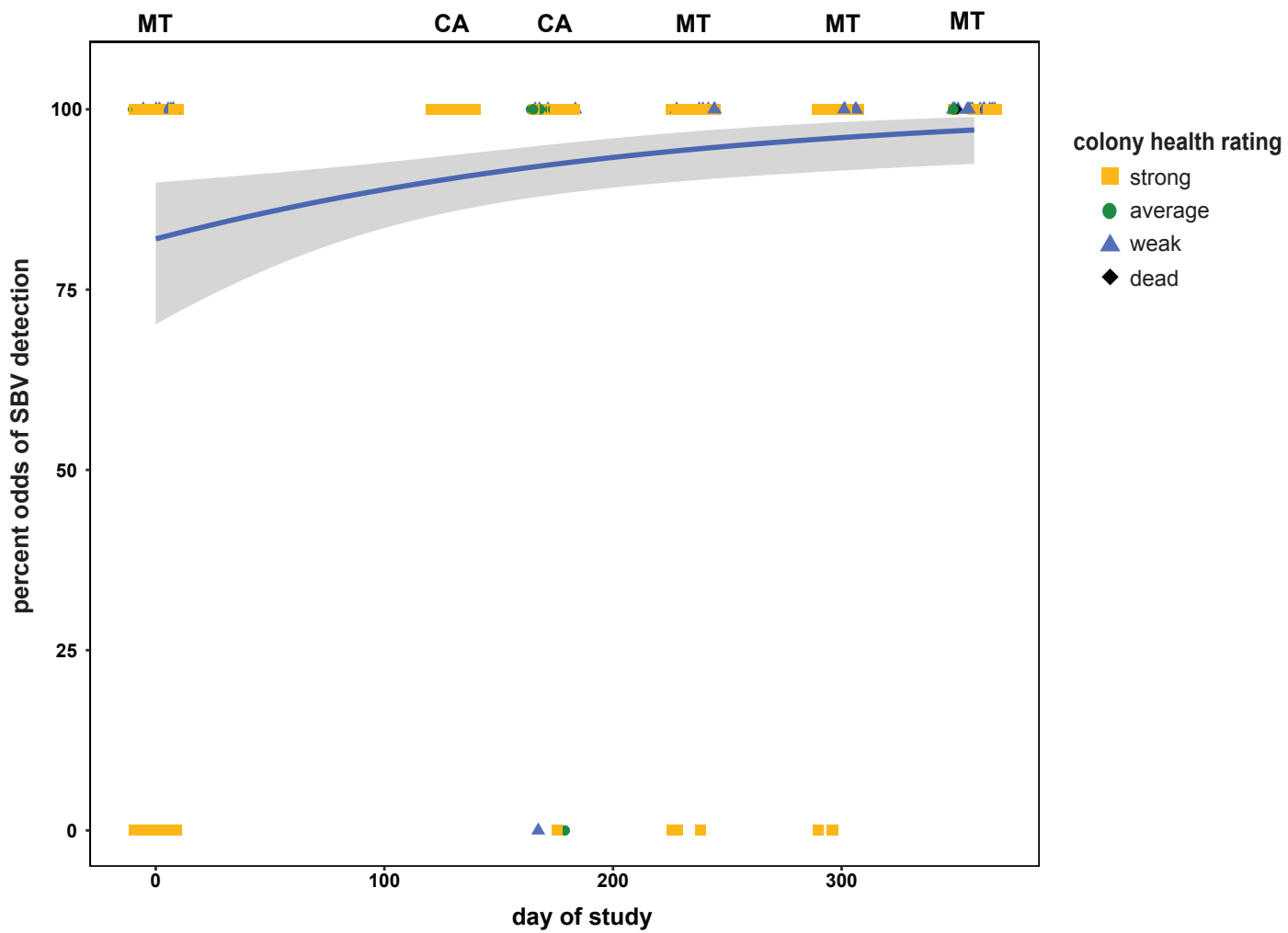

Supporting Figure S4. Odds of detecting SBV over the course of the study.

Supplement: S4 Fig — The odds of detecting SBV increased by 0.005886 (SE +/- 0.0024) with each day of the study (Z-test, z-value = 2.491, p-value = 0.0127). Colony level SBV data for each sample date is represented as colony strength indicating icons (i.e., strong = yellow square, average = green circle, weak = blue triangle, and dead = black diamond). The best fit line (blue) of the odds of detecting SBV in response to day of study was determined with a generalized linear mixed effect model (GLMM) with a binomial family distribution and random effect for individual colony, and is surrounded by upper and lower standard error estimates (gray). (PDF) [file pone.0237544.s004.pdf]

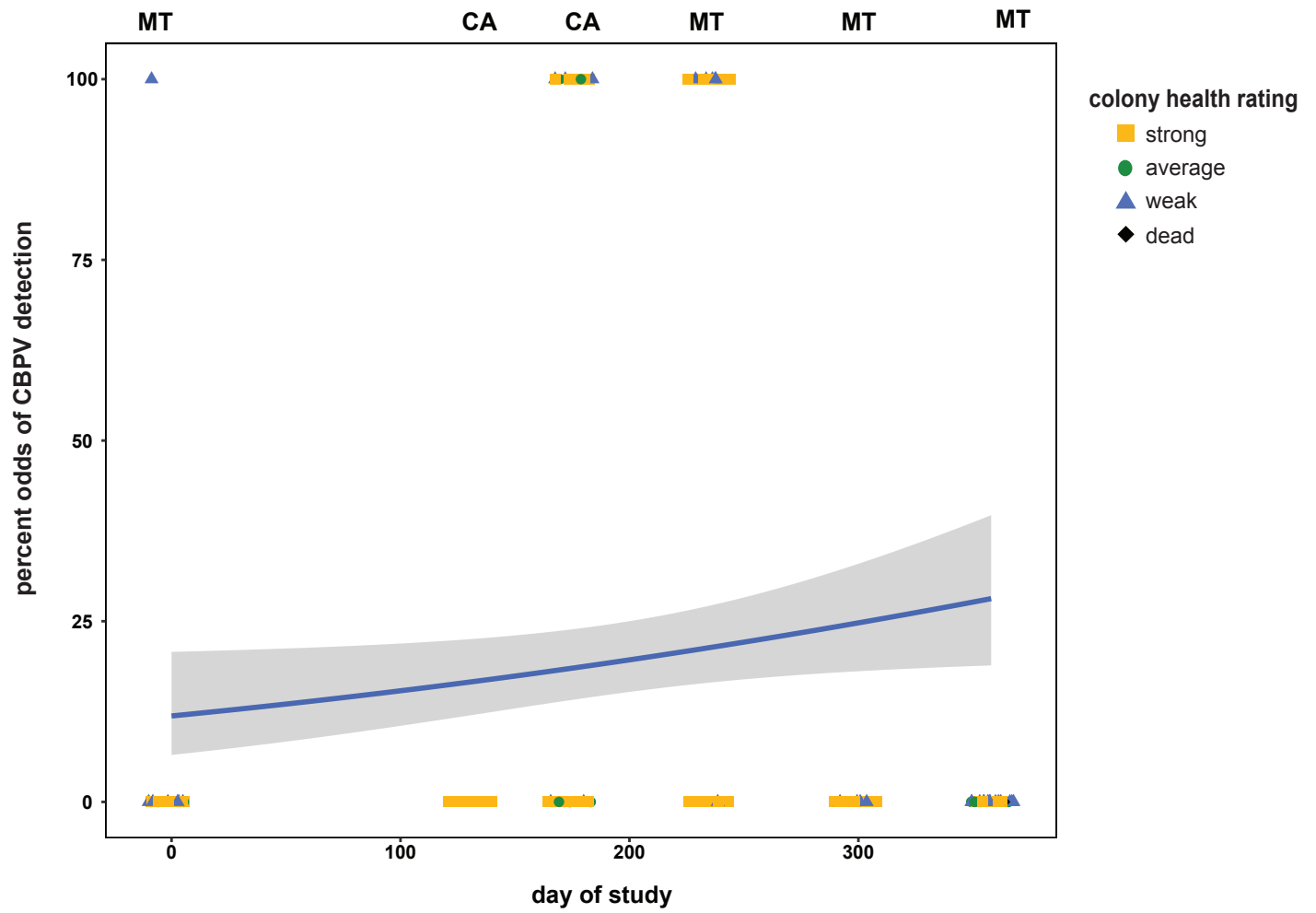

Supporting Figure S5. Odds of detecting CBPV over the course of the study.

Supplement: S5 Fig — The odds of detecting CBPV increased by 0.00345 (SE +/- 0.001) with each day of the study (Z-test, z-value = 2.211, p-value = 0.027). Colony level CBPV data for each sample date is represented as colony strength indicating icons (i.e., strong = yellow square, average = green circle, weak = blue triangle, and dead = black diamond). The best fit line (blue) of the odds of detecting CBPV in response to day of study was determined with a generalized linear mixed effect model (GLMM) with a binomial family distribution and random effect for individual colony, and is surrounded by upper and lower standard error estimates (gray). (PDF) [file pone.0237544.s005.pdf]

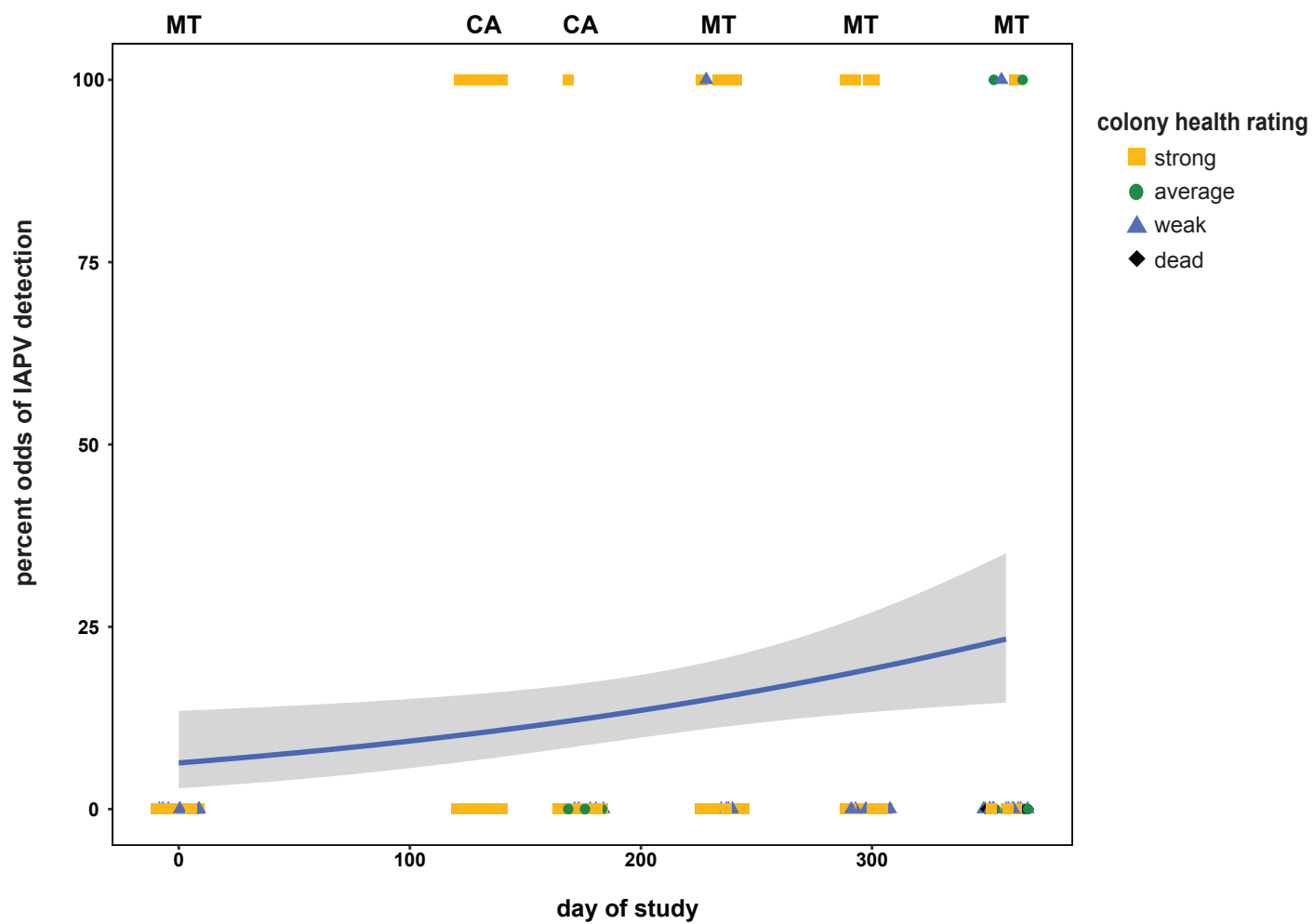

Supporting Figure S6. Odds of detecting IAPV over the course of the study.

Supplement: S6 Fig — The odds of detecting IAPV increased by 0.005 (SE +/- 0.001918) with each of day of the study (z-value = 2.904, p-value = 0.004). Colony level IAPV data for each sample date is represented as colony strength indicating icons (i.e., strong = yellow square, average = green circle, weak = blue triangle, and dead = black diamond). The best fit line (blue) of the odds of detecting IAPV in response to day of study was determined with a generalized linear mixed effect model (GLMM) with a binomial family distribution and random effect for individual colony, and is surrounded by upper and lower standard error estimates (gray). (PDF) [file pone.0237544.s006.pdf]

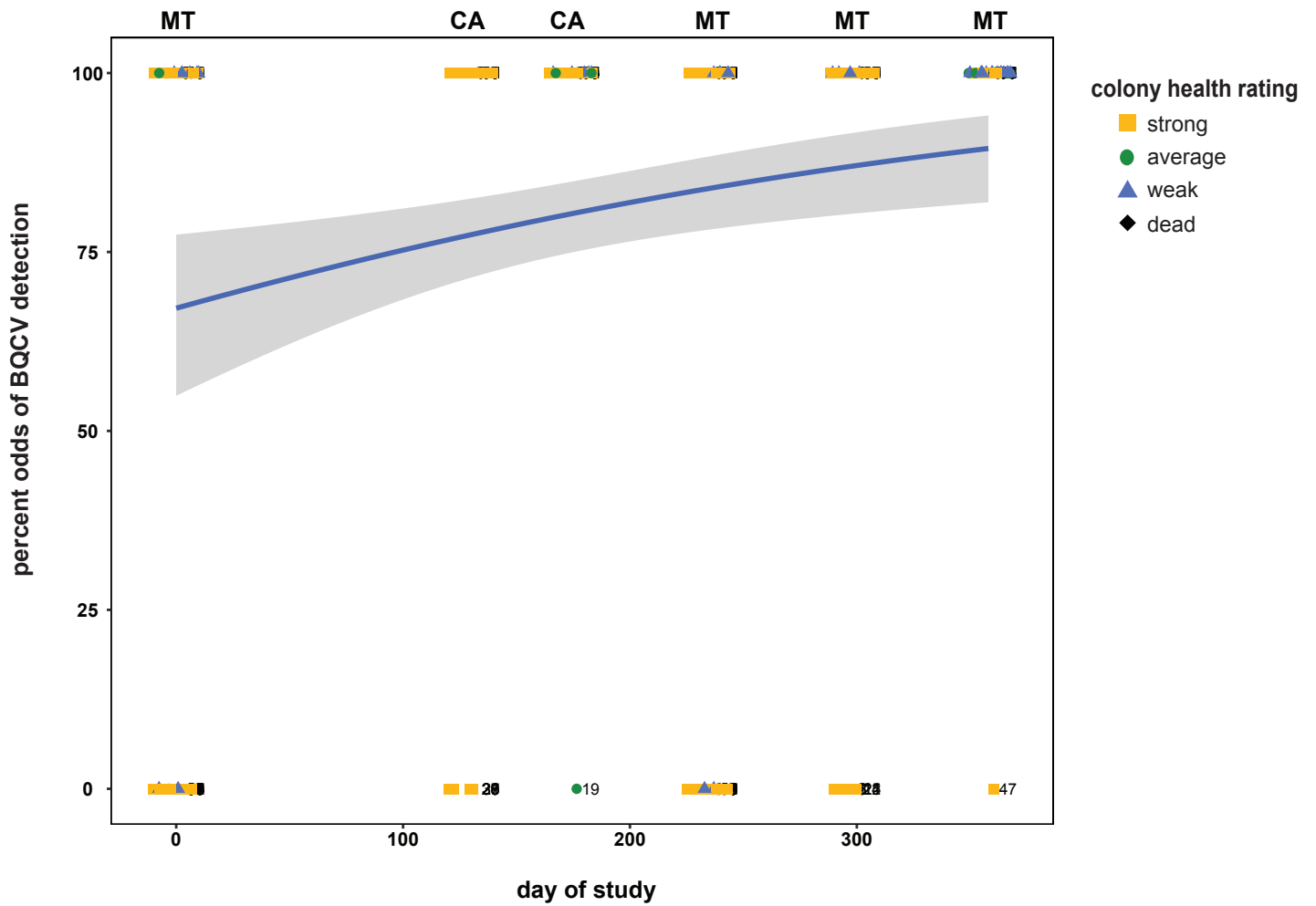

Supporting Figure S7. Odds of detecting BQCV over the course of the study.

Supplement: S7 Fig — The odds of detecting BQCV increased by 0.003 (SE +/- 0.001) with each day of the study (Z-test, z-value = 2.536, p-value = 0.0112). Colony level BQCV data for each sample date is represented as colony strength indicating icons (i.e., strong = yellow square, average = green circle, weak = blue triangle, and dead = black diamond). The best fit line (blue) of the odds of detecting BQCV in response to day of study was determined with a generalized linear mixed effect model (GLMM) with a binomial family distribution and random effect for individual colony, and is surrounded by upper and lower standard error estimates (gray). (PDF) [file pone.0237544.s007.pdf]

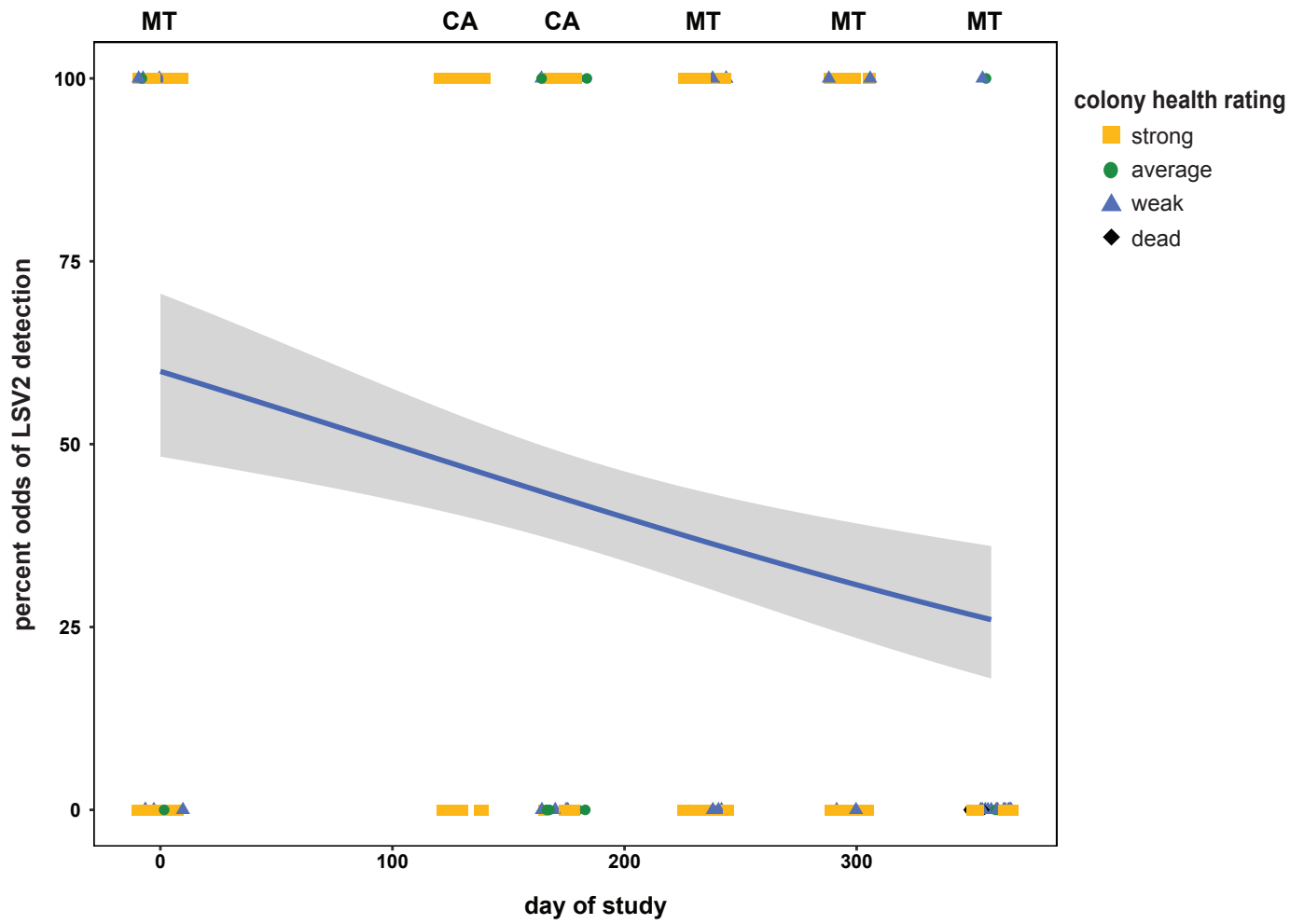

Supporting Figure S8. Percent odds of detecting LSV2 over the course of the study.

Supplement: S8 Fig — The odds of detecting LSV2 decreased by 0.0037 (SE +/- 0.0012) per day (Z-test, z-value = -3.098, p-value = 0.00795). Colony level LSV2 data for each sample date is represented as colony strength indicating icons (i.e., strong = yellow square, average = green circle, weak = blue triangle, and dead = black diamond). The best fit line (blue) of the odds of detecting LSV2 in response to day of study was determined with a generalized linear mixed effect model (GLMM) with a binomial family distribution and random effect for individual colony and is surrounded by upper and lower standard error estimates (gray). (PDF) [file pone.0237544.s008.pdf]

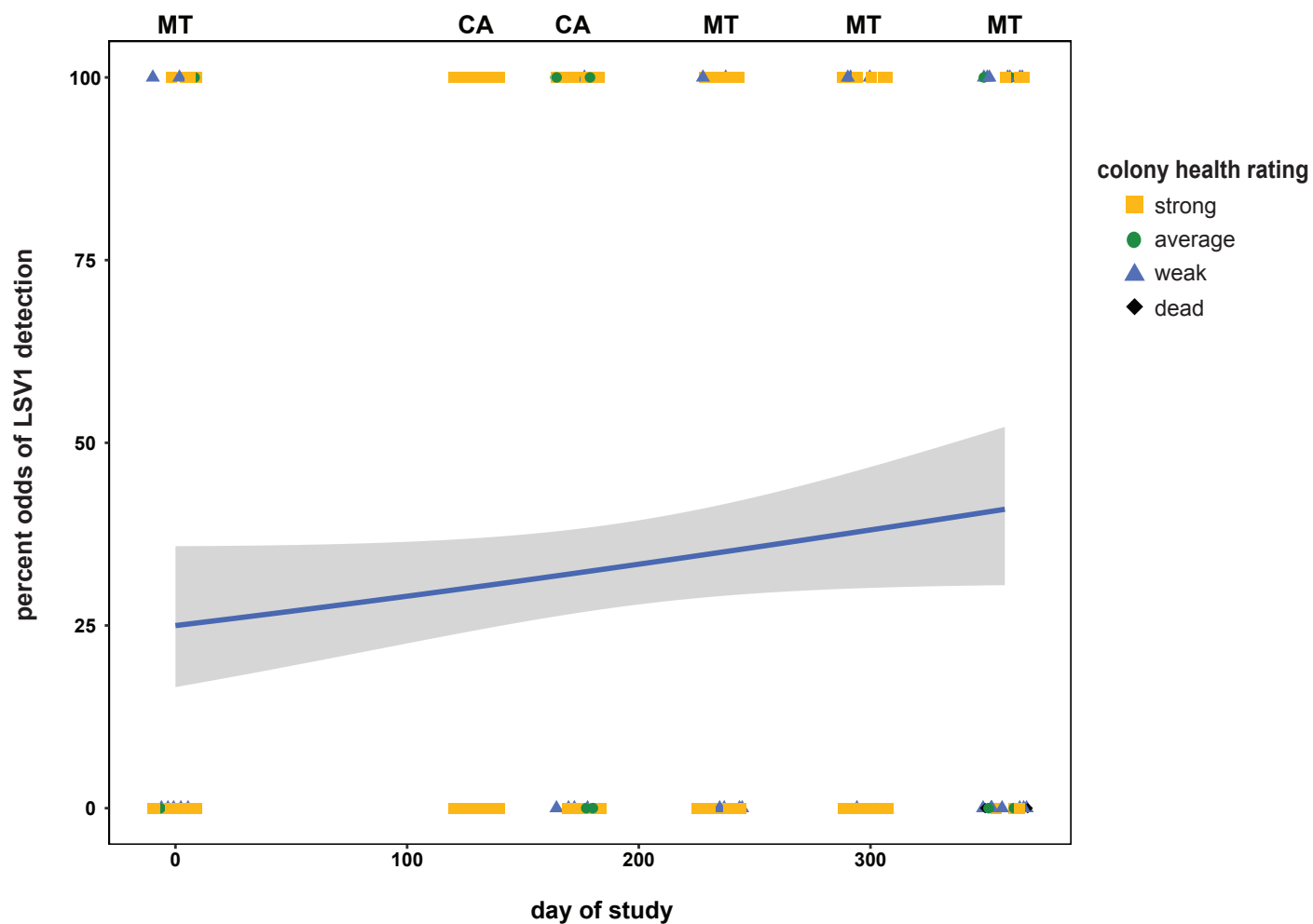

Supporting Figure S9. Percent odds of detecting LSV1 over the course of the study.

Supplement: S9 Fig — The odds of detecting LSV1 did not vary over the course of the study. LSV1 abundance was collapsed into a binary response to account for frequent zeros in the response distribution. Colony level LSV1 data for each sample date is represented as colony strength indicating icons (i.e., strong = yellow square, average = green circle, weak = blue triangle, and dead = black diamond). The best fit line (blue) of the odds of detecting LSV1 in response to day of study was determined with a generalized linear mixed effect model (GLMM) with a binomial family distribution and random effect for individual colony and is surrounded by upper and lower standard error estimates (gray). (PDF) [file pone.0237544.s009.pdf]

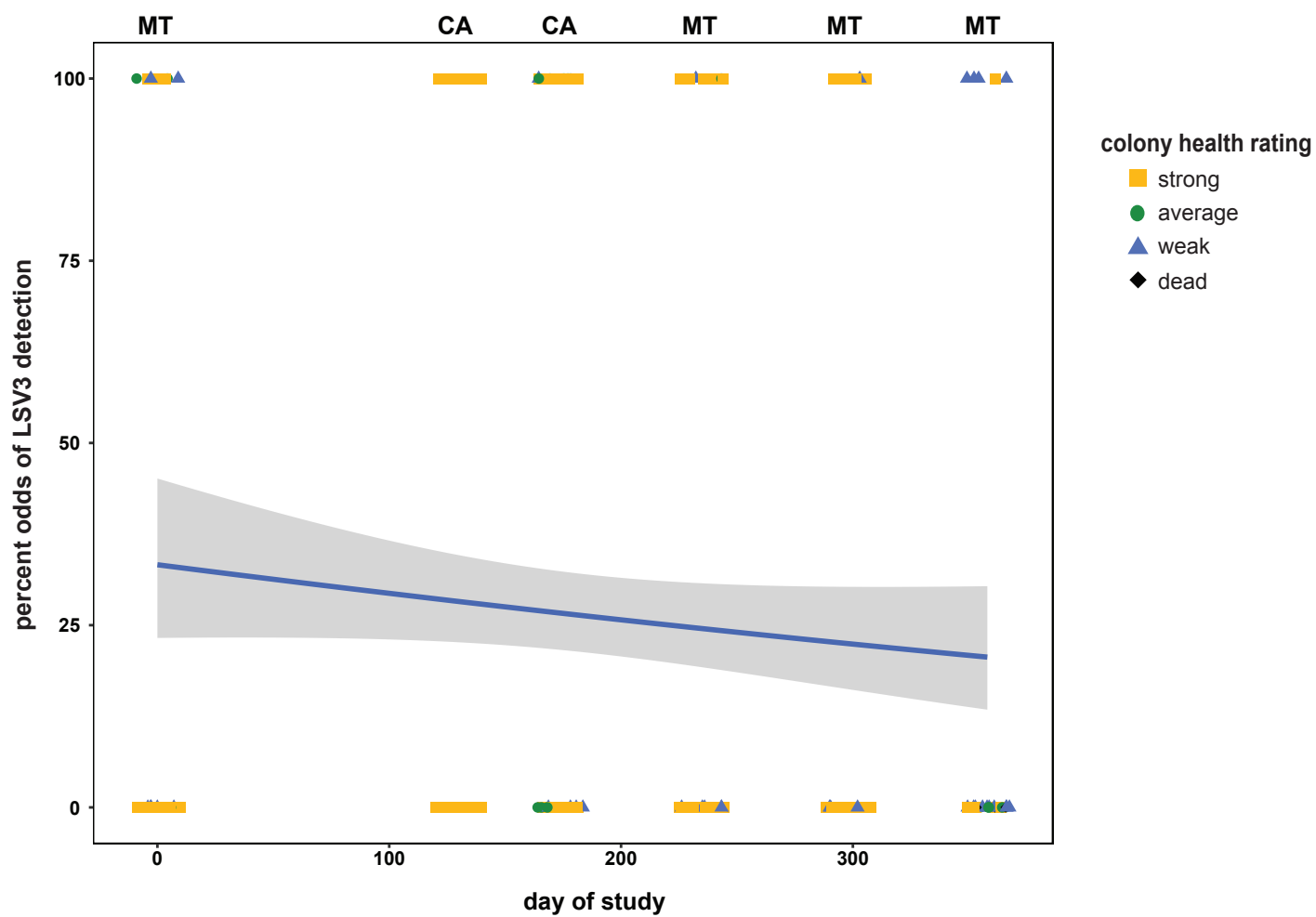

Supporting Figure S10. Percent odds of detecting LSV3 over the course of the study.

Supplement: S10 Fig — The odds of detecting LSV3 did not vary over the course of the study. Colony level LSV3 data for each sample date is represented as colony strength indicating icons (i.e., strong = yellow square, average = green circle, weak = blue triangle, and dead = black diamond). The best fit line (blue) of the odds of detecting LSV3 in response to day of study was determined with a generalized linear mixed effect model (GLMM) with a binomial family distribution and random effect for individual colony, and is surrounded by upper and lower standard error estimates (gray). (PDF) [file pone.0237544.s010.pdf]

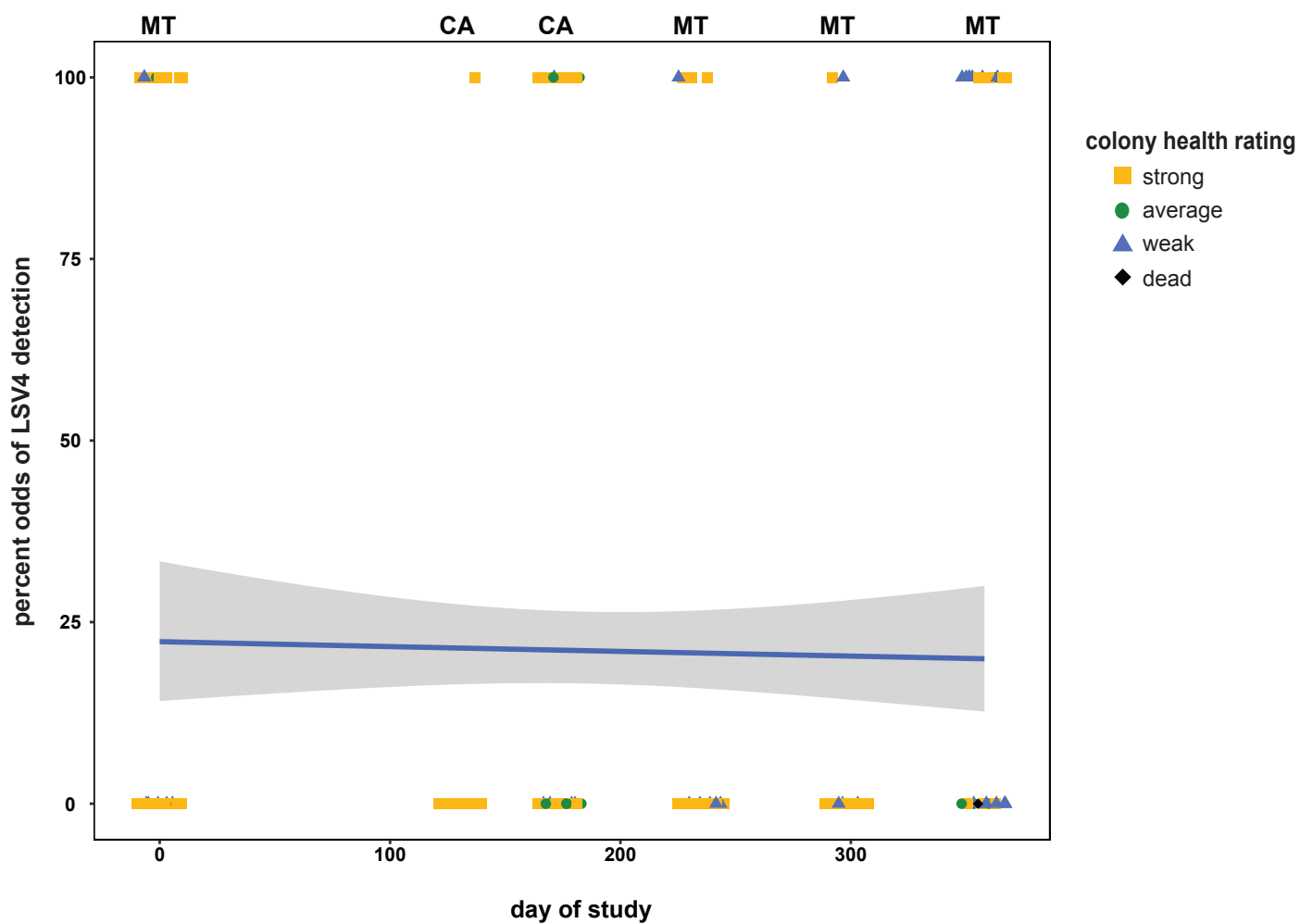

Supporting Figure S11. Odds of detecting LSV4 over the course of the study.

Supplement: S11 Fig — The odds of detecting LSV4 did not vary over the course of the study. Colony level LSV4 data for each sample date is represented as colony strength indicating icons (i.e., strong = yellow square, average = green circle, weak = blue triangle, and dead = black diamond). The best fit line (blue) of the odds of detecting LSV4 in response to day of study was determined with a generalized linear mixed effect model (GLMM) with a binomial family distribution and random effect for individual colony, and is surrounded by upper and lower standard error estimates (gray). (PDF) [file pone.0237544.s011.pdf]

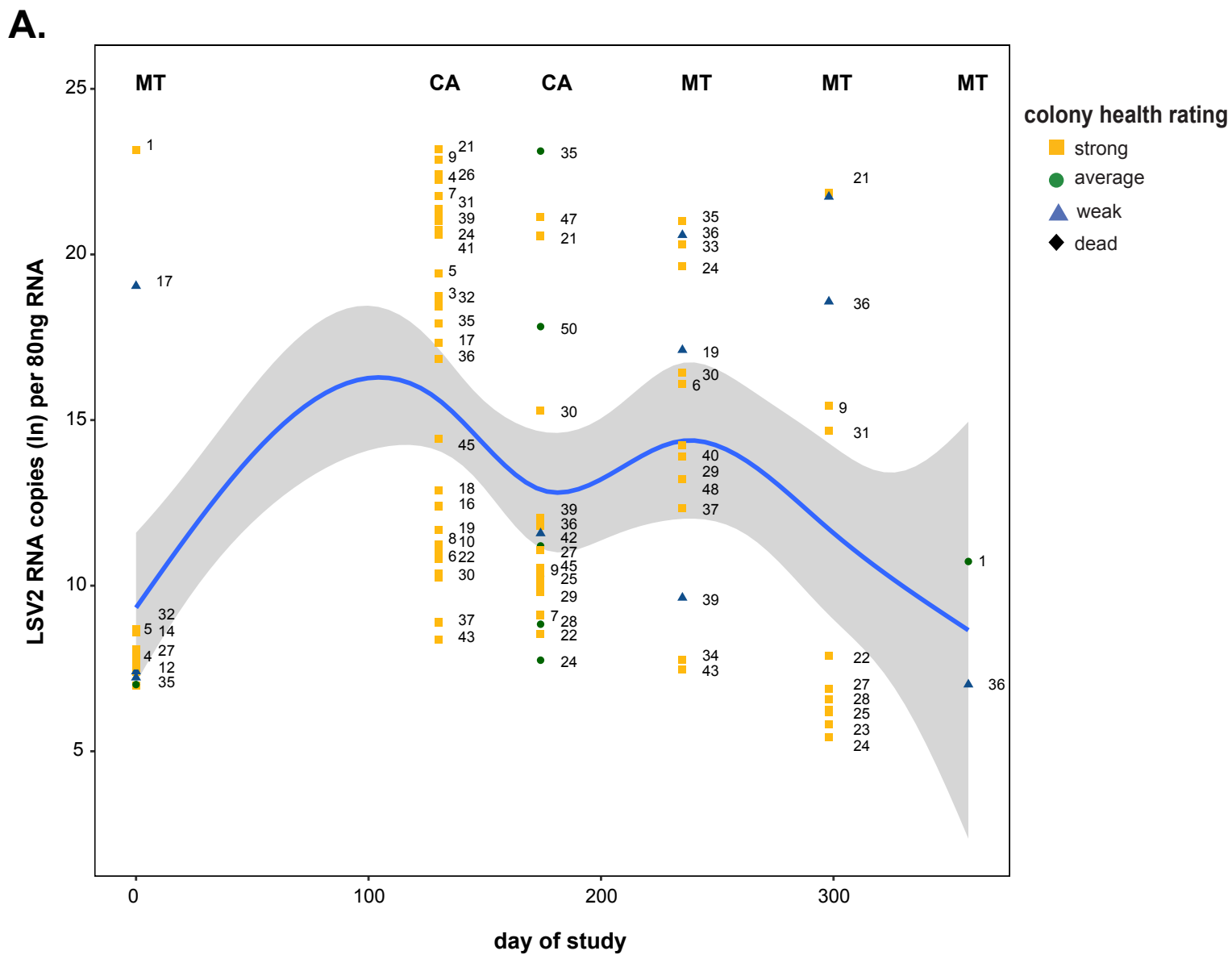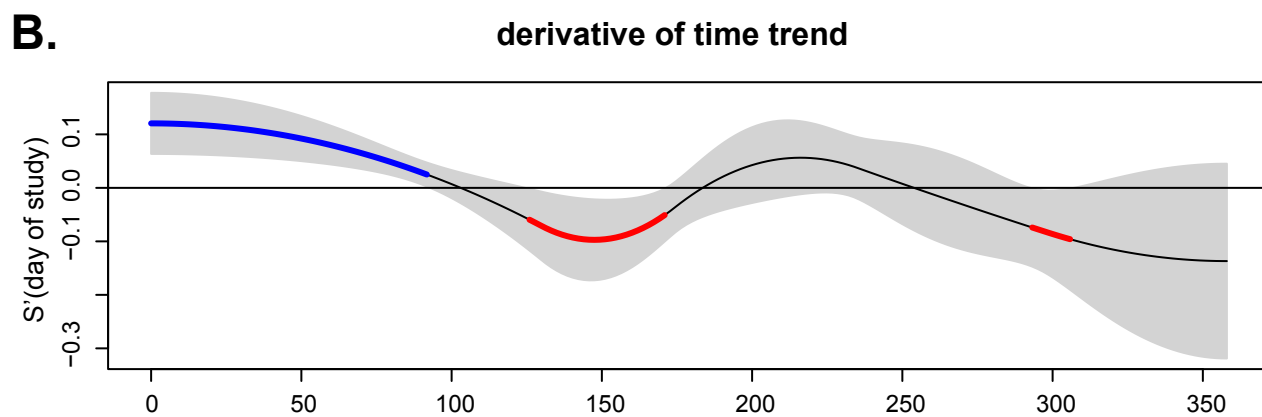

Supporting Figure S12. Changes in LSV2 abundance in honey bee colonies throughout the study.

Supplement: S12 Fig — A. LSV2 abundance varied over time and was lowest at the end of the study (day of studyedf = 4.281, p-value = 0.0001). The natural log transformed LSV2 abundance data as determined by qPCR in honey bee samples were plotted by day of study. The best fit line (blue) for LSV2 mixed model (GAMM) and is surrounded by upper and lower standard error estimates (gray). Colony level LSV2 data for each sample date is represented as colony strength indicating icons (i.e., strong = yellow square, average = green circle, weak = blue triangle, and dead = black diamond) with unique colony identifier numbers, which illustrate the changes in virus abundance of individual colonies throughout the study. B. The first derivative of the fitted spline in A was calculated to identify the rate of change of LSV2 abundance throughout the timeframe and 95% confidence intervals (gray) were built around the first derivative to distinguish periods of time when the change in virus abundance is significantly different from zero. LSV2 abundance significantly increased (blue) from 0 to 100 days of the study and significantly decreased (red) from 100 to 175 days and from 300 to 350 days of the study. (PDF) [file pone.0237544.s012.pdf]

**A.**

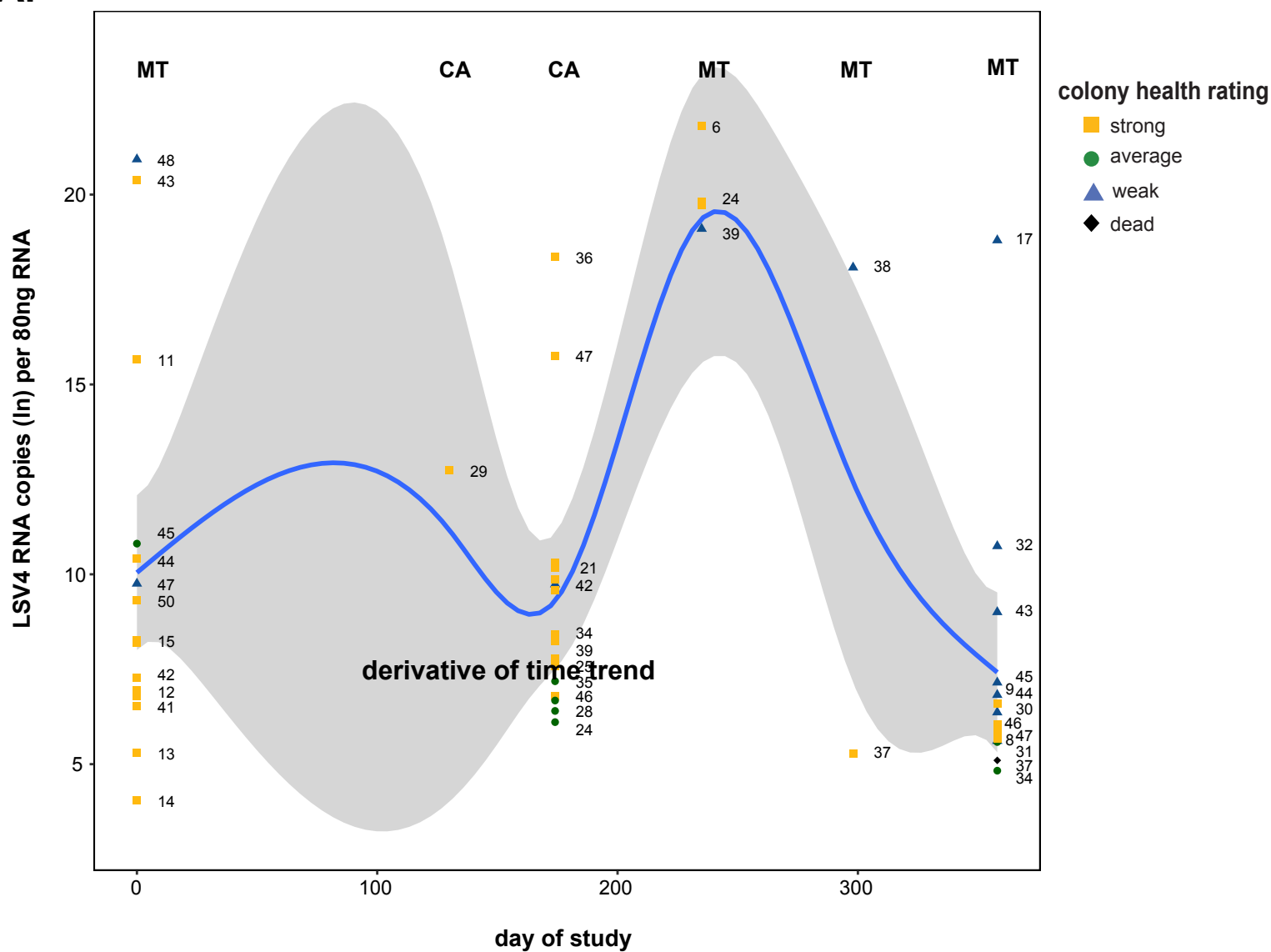

**B.**

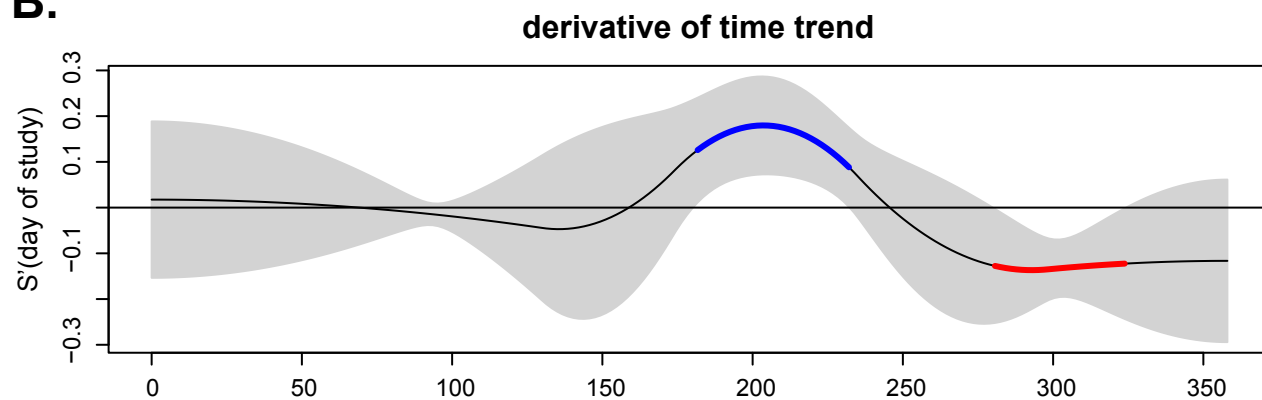

**Supporting Figure S13. Changes in LSV4 RNA abundance in honey bee colonies throughout the study.**

Supplement: S13 Fig — A. LSV4 abundance varied over the course of the study (day of studyedf = 4.071, p-value = 0.000122). The natural log transformed LSV4 abundance data as determined by qPCR (y-axis) in honey bee samples were plotted by day of study (x-axis). The best fit line (blue) for LSV4 mixed model (GAMM) and is surrounded by upper and lower standard error estimates (gray). Colony level LSV4 data for each sample date is represented as colony strength indicating icons (i.e., strong = yellow square, average = green circle, weak = blue triangle, and dead = black diamond) with unique colony identifier numbers, which illustrate the changes in virus abundance of individual colonies throughout the study. B. The first derivative of the fitted spline in panel A was calculated to identify the rate of change of LSV4 abundance throughout the timeframe and 95% confidence intervals (gray) were built around the first derivative to distinguish periods of time when the change in virus abundance is significantly different from zero. LSV4 abundance significantly increased (blue) from 175 to 225 days and decreased (red) from 275 to 325 days of the study. (PDF) [file pone.0237544.s013.pdf]

A.

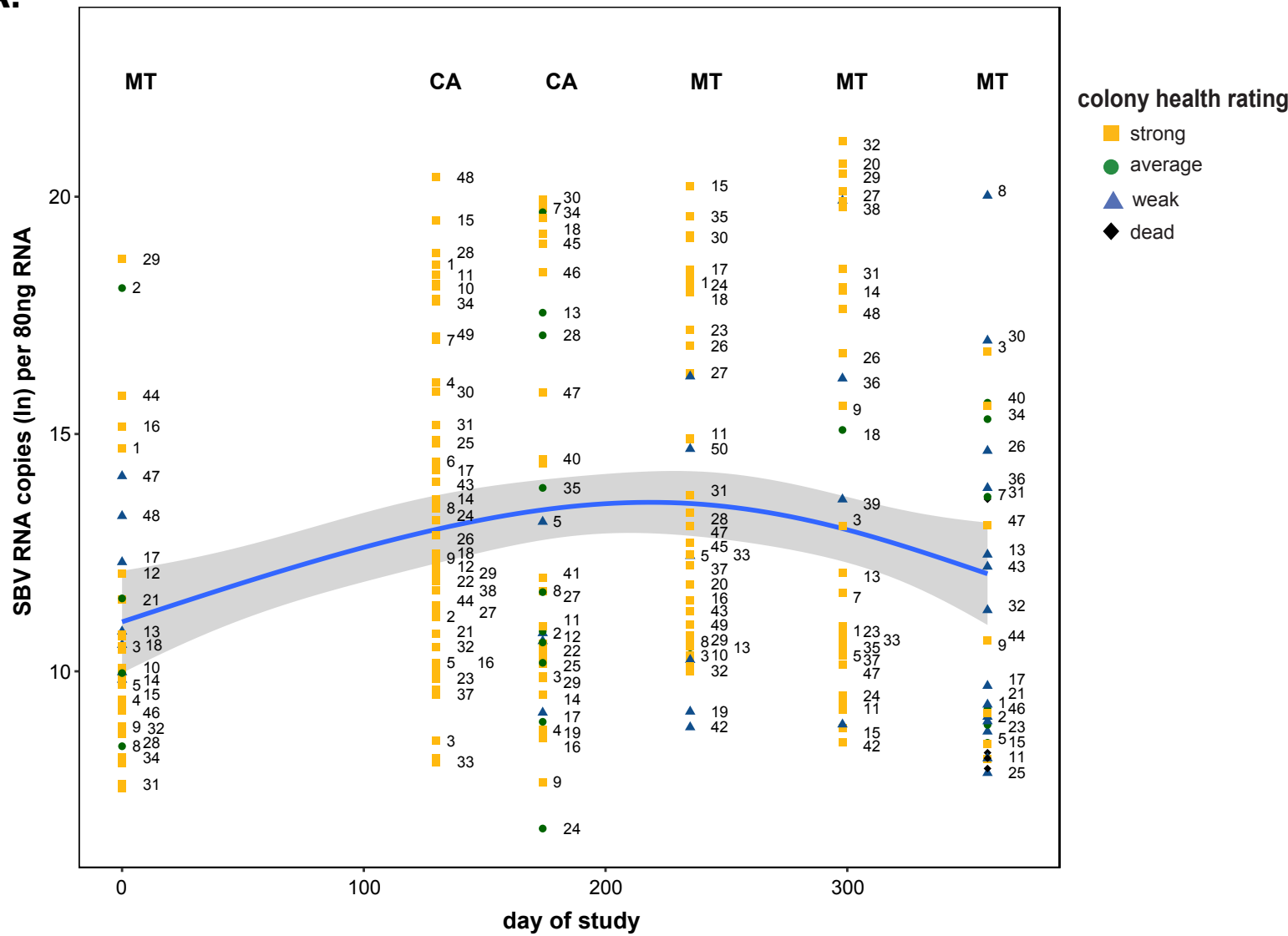

B.

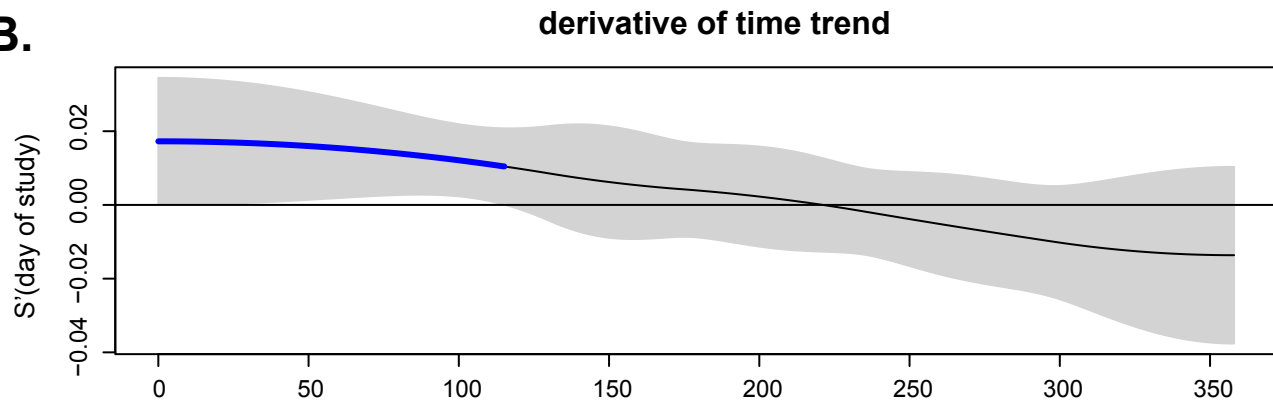

Supporting Figure S14. Changes in SBV abundance in honey bee colonies throughout the study.

Supplement: S14 Fig — A. SBV abundance varied over the course of the study (day of studyedf = 2.252, p-value = 0.00348). The natural log transformed SBV abundance data as determined by qPCR (y-axis) in honey bee samples were plotted by day of study (x-axis). The best fit line (blue) for SBV mixed model (GAMM) and is surrounded by upper and lower standard error estimates (gray). Colony level SBV data for each sample date is represented as colony strength indicating icons (i.e., strong = yellow square, average = green circle, weak = blue triangle, and dead = black diamond) with unique colony identifier numbers, which illustrate the changes in virus abundance of individual colonies throughout the study. B. The first derivative of the fitted spline in panel A was calculated to identify the rate of change of SBV abundance throughout the timeframe and 95% confidence intervals (gray) were built around the first derivative to distinguish periods of time when the change in virus abundance is significantly different from zero. SBV abundance significantly increased (blue) from 0 to 100 days of the study, then did not vary for the remainder of the study. (PDF) [file pone.0237544.s014.pdf]

**A.**

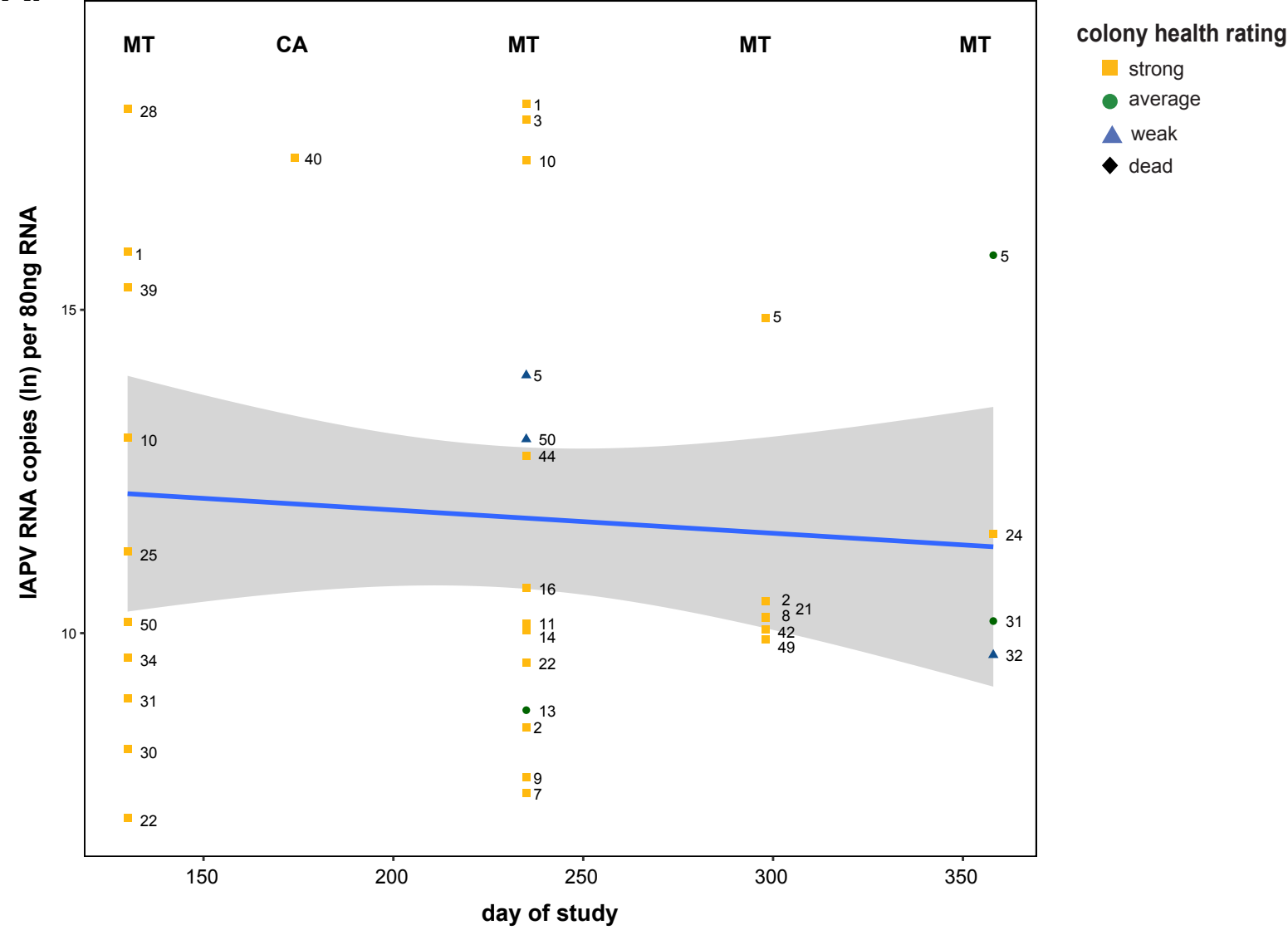

**B.**

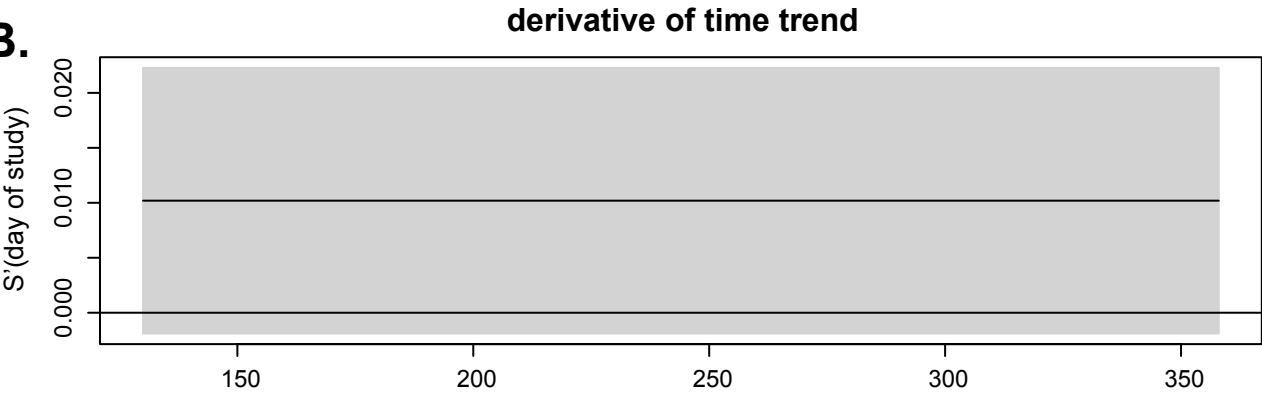

Supporting Figure S15. Changes in IAPV abundance in honey bee colonies throughout the study.

Supplement: S15 Fig — A. IAPV abundance decreased linearly over the course of the study (day of studyedf = 1, p-value = 0.0274). The natural log transformed IAPV abundance data as determined by qPCR (y-axis) in honey bee samples were plotted by day of study (x-axis). The best fit line (blue) for IAPV mixed model (GAMM) and is surrounded by upper and lower standard error estimates (gray). Colony level IAPV data for each sample date is represented as colony strength indicating icons (i.e., strong = yellow square, average = green circle, weak = blue triangle, and dead = black diamond) with unique colony identifier numbers, which illustrate the changes in virus abundance of individual colonies throughout the study. B. The first derivative of the fitted spline in panel A was calculated to identify the rate of change of IAPV abundance throughout the timeframe and 95% confidence intervals (gray) were built around the first derivative to distinguish periods of time when the change in virus abundance is significantly different from zero. There were no periods of significant change in the derivative. (PDF) [file pone.0237544.s015.pdf]

A.

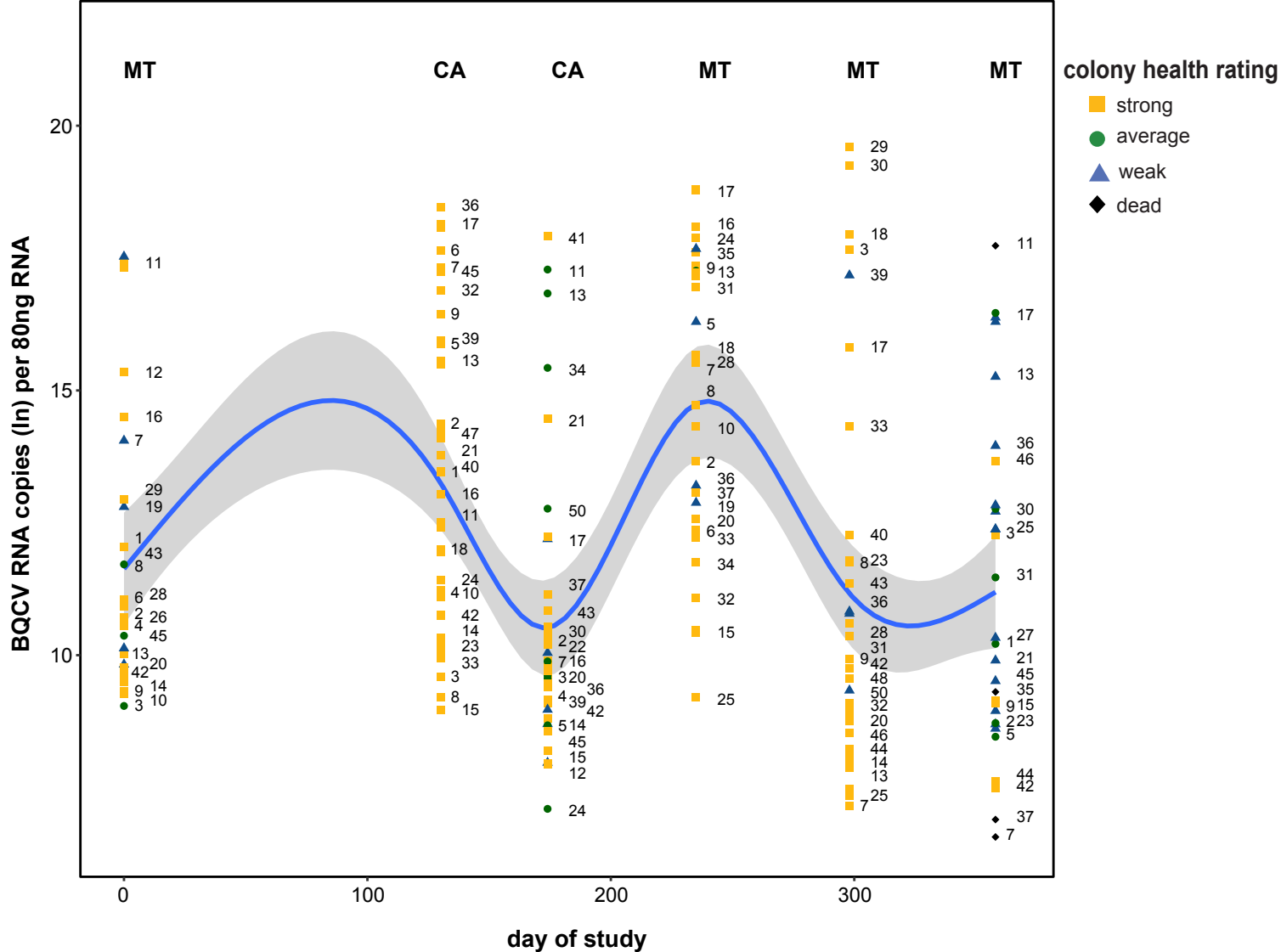

B.

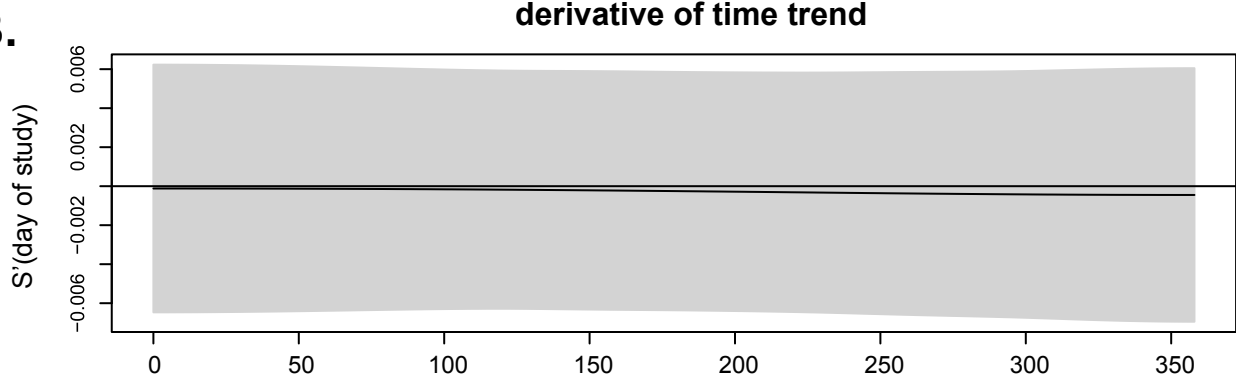

Supporting Figure S16. Changes in BQCV abundance in honey bee colonies throughout the study.

Supplement: S16 Fig — A. BQCV did not vary over the course of the study (day of studyedf = 1.024, p-value = 0.9107). The natural log transformed BQCV abundance data as determined by qPCR (y-axis) in honey bee samples were plotted by day of study (x-axis). The best fit line (blue) for BQCV mixed model (GAMM) and is surrounded by upper and lower standard error estimates (gray). Colony level BQCV data for each sample date is represented as colony strength indicating icons (i.e., strong = yellow square, average = green circle, weak = blue triangle, and dead = black diamond) with unique colony identifier numbers, which illustrate the changes in virus abundance of individual colonies throughout the study. B. The first derivative of the fitted spline in panel A was calculated to identify the rate of change of BQCV abundance throughout the timeframe and 95% confidence intervals (gray) were built around the first derivative to distinguish periods of time when the change in virus abundance is significantly different from zero. There were no periods of significant change in the derivative. (PDF) [file pone.0237544.s016.pdf]

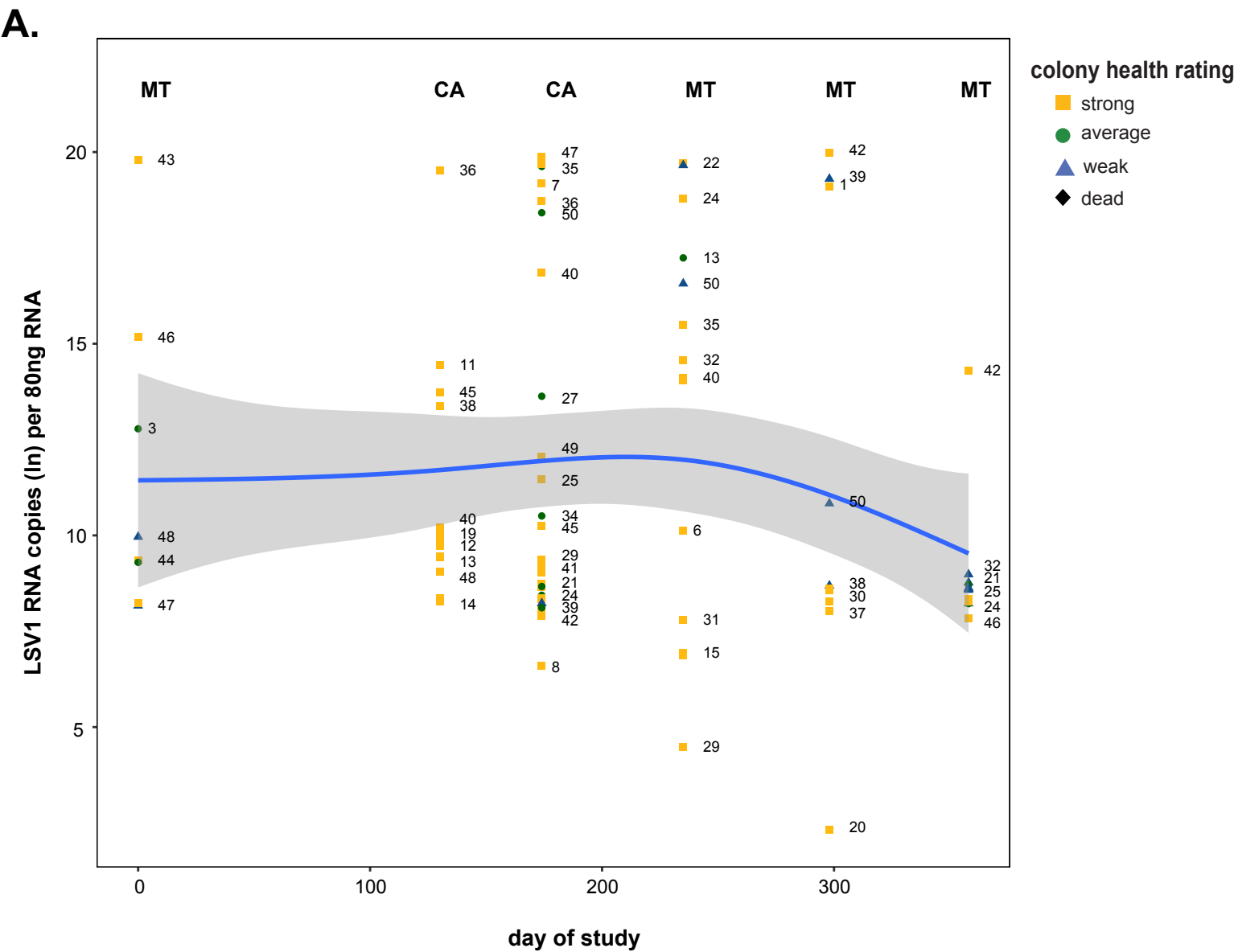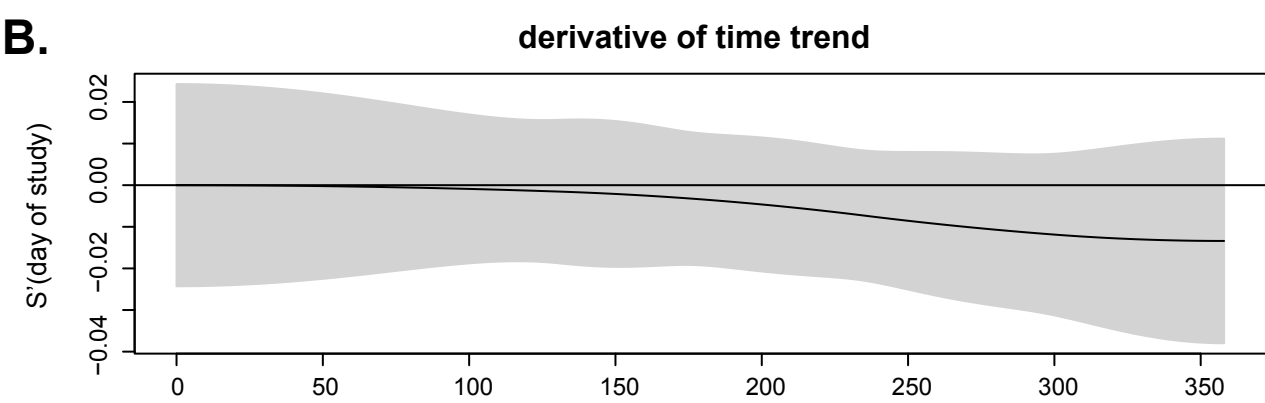

Supporting Figure S17. Changes in LSV1 abundance in honey bee colonies throughout the study.

Supplement: S17 Fig — A. LSV1 did not vary over the course of the study (day of studyedf = 1.428, p-value = 0.286). The natural log transformed LSV1 abundance data as determined by qPCR (y-axis) in honey bee samples were plotted by day of study (x-axis). The best fit line (blue) for LSV1 mixed model (GAMM) and is surrounded by upper and lower standard error estimates (gray). Colony level LSV1 data for each sample date is represented as colony strength indicating icons (i.e., strong = yellow square, average = green circle, weak = blue triangle, and dead = black diamond) with unique colony identifier numbers, which illustrate the changes in virus abundance of individual colonies throughout the study. B. The first derivative of the fitted spline in panel A was calculated to identify the rate of change of LSV1 abundance throughout the timeframe and 95% confidence intervals (gray) were built around the first derivative to distinguish periods of time when the change in virus abundance is significantly different from zero. There were no periods of significant change in the derivative. (PDF) [file pone.0237544.s017.pdf]

**A.**

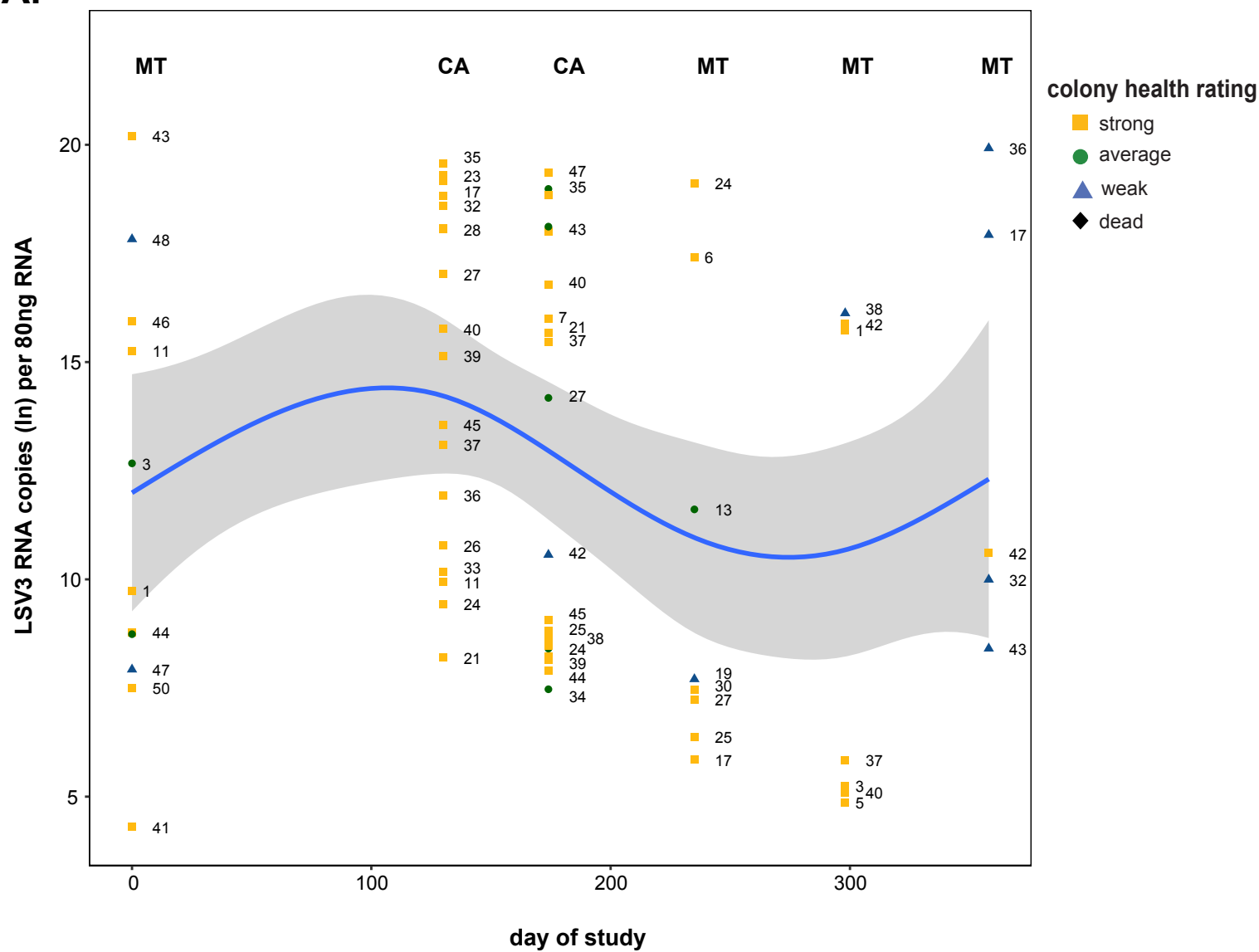

**B.**

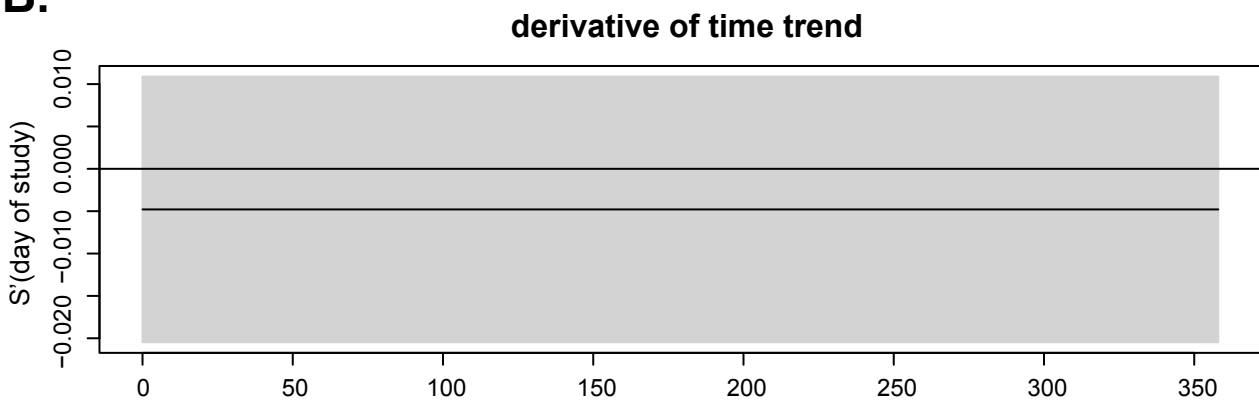

**Supporting Figure S18. Changes in LSV3 abundance in honey bee colonies throughout the study.**

Supplement: S18 Fig — A. LSV3 did not vary over the course of the study (day of studyedf = 1, p-value = 0.420). The natural log transformed LSV3 abundance data as determined by qPCR (y-axis) in honey bee samples were plotted by day of study (x-axis). The best fit line (blue) for LSV3 mixed model (GAMM) and is surrounded by upper and lower standard error estimates (gray). Colony level LSV3 data for each sample date is represented as colony strength indicating icons (i.e., strong = yellow square, average = green circle, weak = blue triangle, and dead = black diamond) with unique colony identifier numbers, which illustrate the changes in virus abundance of individual colonies throughout the study. B. The first derivative of the fitted spline in panel A was calculated to identify the rate of change of LSV1 abundance throughout the timeframe and 95% confidence intervals (gray) were built around the first derivative to distinguish periods of time when the change in virus abundance is significantly different from zero. There were no periods of significant change in the derivative. (PDF) [file pone.0237544.s018.pdf]

Supporting Figure S19. Relative virus composition of honey bee colonies by colony health.

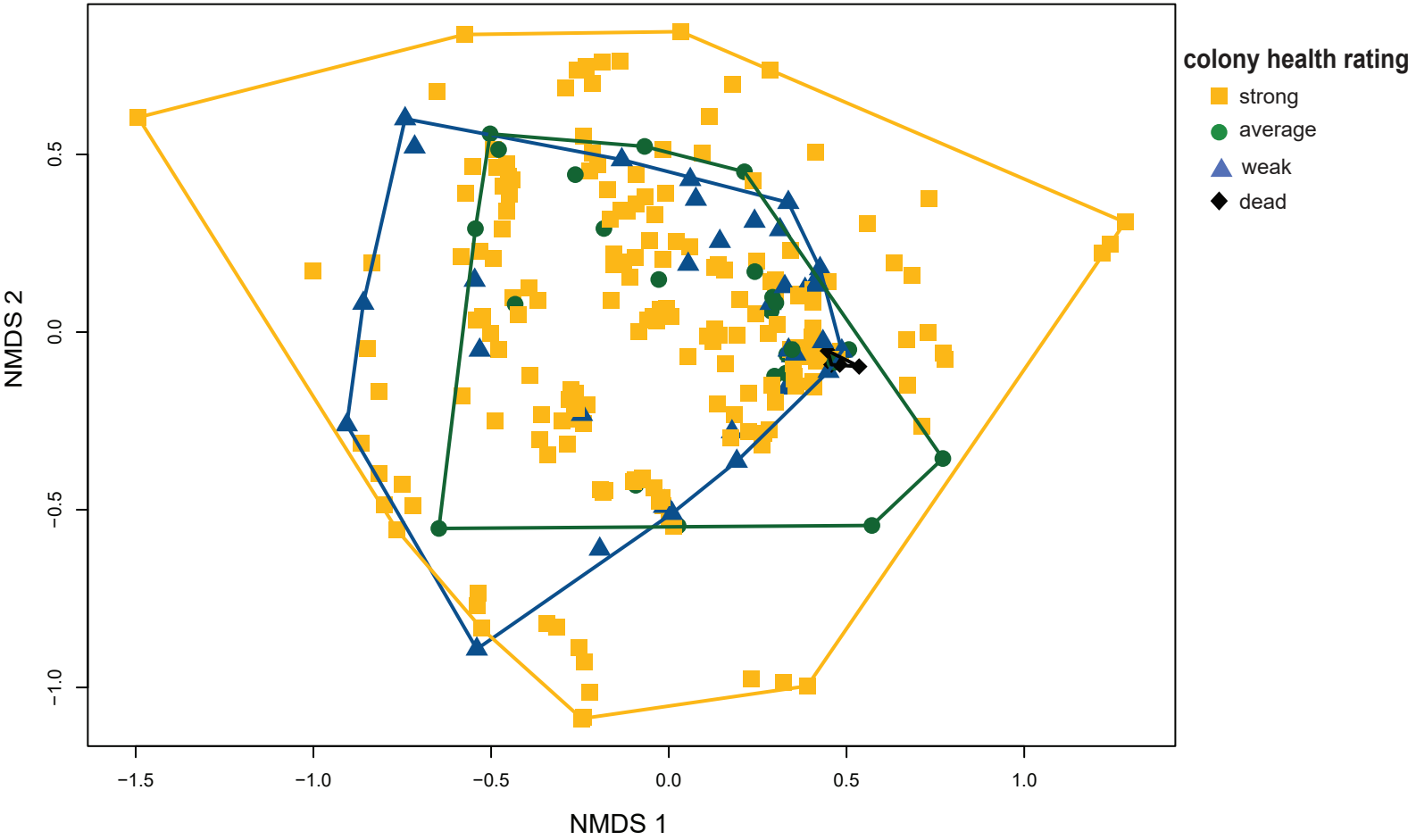

Supplement: S19 Fig — Pathogen community composition did not vary by colony health (PERMANOVA, F3,255 = 1.35, p-value = 0.191). The virus community composition for each sample (i.e., the log natural transformed abundance of BQCV, CBPV, DWV, SBV, LSV1, LSV2, LSV3, LSV4, and IAPV, as assessed by qPCR) was compared in relation to colony health rating (i.e., weak, average, and strong). The position of each point indicates the virus composition of each sample relative to all other samples (i.e., samples with more similar virus composition are closer), calculated using a Bray-Curtis dissimilarity index and plotted on a non-metric multidimensional scaling (NMDS) plot. (PDF) [file pone.0237544.s019.pdf]
