# Supplementary material for: Longitudinal monitoring of honey bee colonies reveals dynamic nature of virus abundance and indicates a negative impact of Lake Sinai virus 2 on colony health
Source: PLoS One. 2020 Sep 8;15(9):e0237544. doi: 10.1371/journal.pone.0237544 (PMC7478651; doi:10.1371/journal.pone.0237544)
Supplement: S2 Fig — The observed incidence of all the pathogens monitored in this study (i.e., BQCV, DWV, SBV, C.m./L.p., Nos., LSV1, LSV2, LSV3, LSV4, CBPV, IAPV, ABPV, and KBV) are represented as a percentage of all samples at each time point (dark gray = positive). (PDF) [file pone.0237544.s002.pdf]

Supporting Figure S2

Pathogen incidence in honey bee colonies throughout the study for all monitored pathogens.

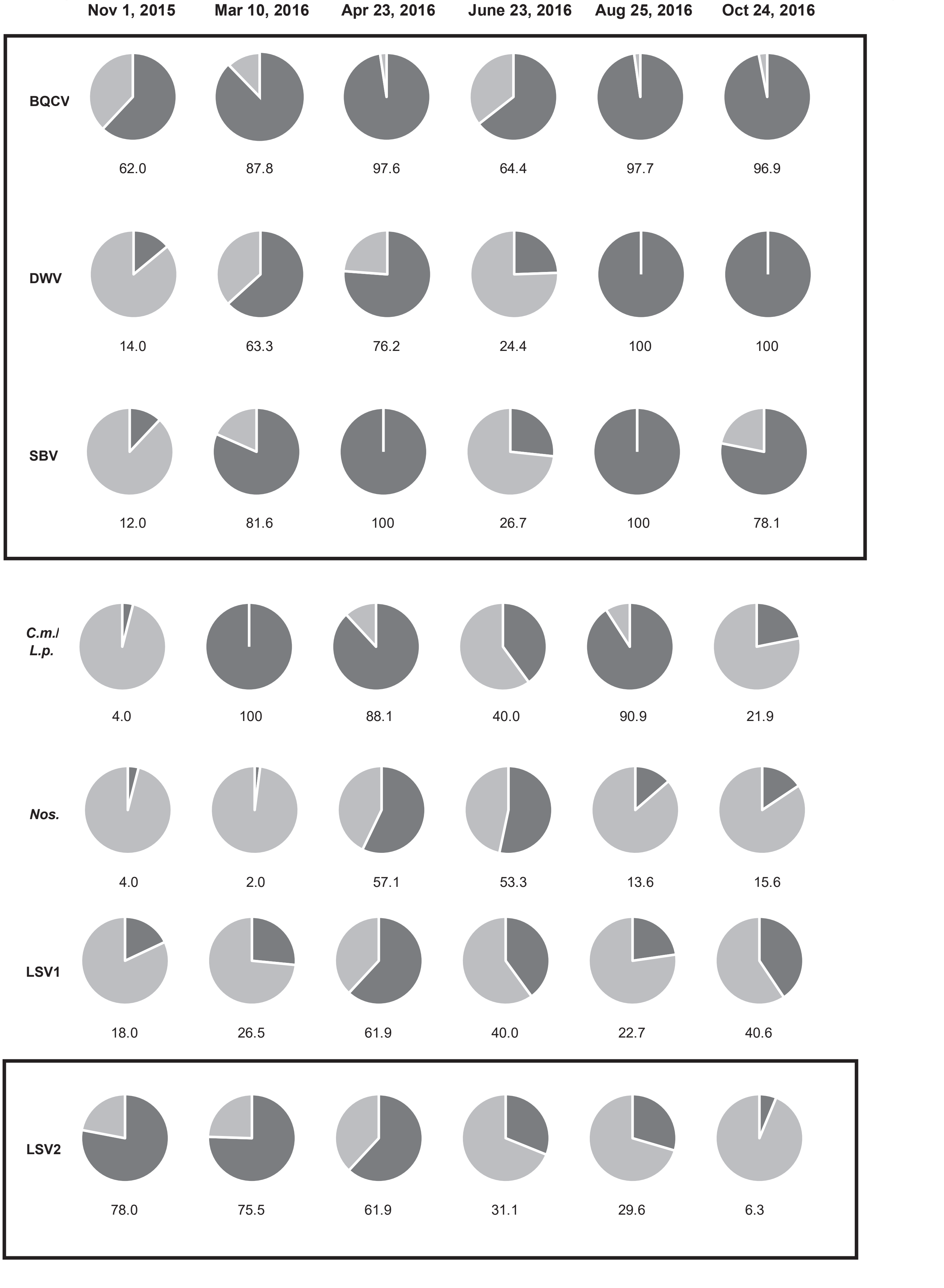

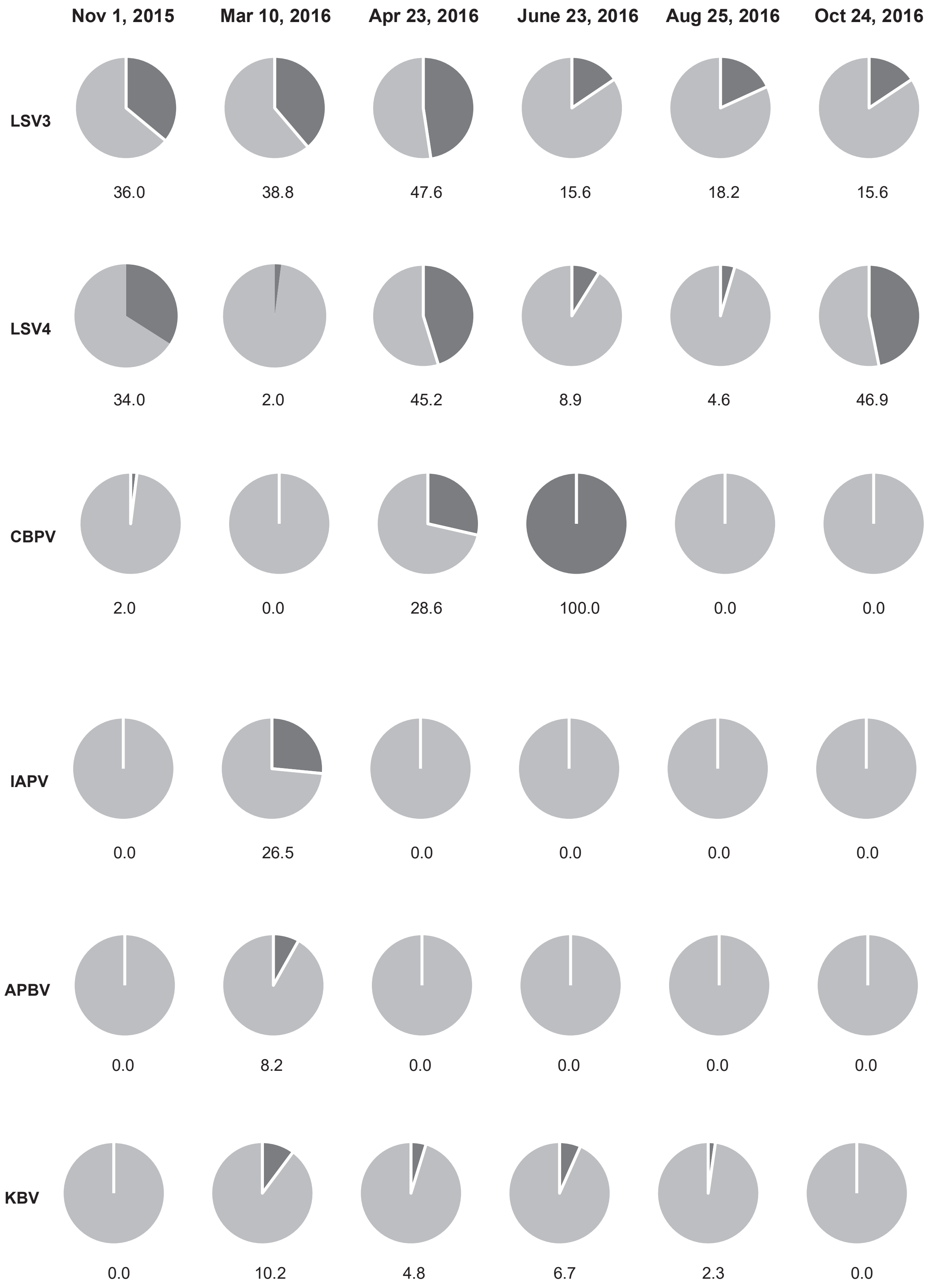
Nov 1, 2015
Mar 10, 2016
Apr 23, 2016
June 23, 2016
Aug 25, 2016
Oct 24, 2016
LSV3
36.0
38.8
47.6
15.6
18.2
15.6
LSV4
34.0
2.0
45.2
8.9
4.6
46.9
CBPV
2.0
0.0
28.6
100.0
0.0
0.0
IAPV
0.0
26.5
0.0
0.0
0.0
0.0
APBV
0.0
8.2
0.0
0.0
0.0
0.0
KBV
0.0
10.2
4.8
6.7
2.3
0.0
