# Supplementary material for: Longitudinal monitoring of honey bee colonies reveals dynamic nature of virus abundance and indicates a negative impact of Lake Sinai virus 2 on colony health
Source: PLoS One. 2020 Sep 8;15(9):e0237544. doi: 10.1371/journal.pone.0237544 (PMC7478651; doi:10.1371/journal.pone.0237544)
Supplement: S3 Fig — Pathogen prevalence did not vary by colony health rating in this sample cohort. Honey bee samples obtained from dead, weak, average, and strong colonies were tested for the presence of 13 pathogens (i.e., ABPV, BQCV, CBPV, DWV, IAPV, KBV, LSV1, LSV2, LSV3, LSV4, SBV, L. passim, and N. ceranae) using pathogen specific PCR. Total pathogen prevalence refers to the sum of the different pathogens detected in each sample. The mean of the total number of pathogens per colony strength rating, the standard error estimate of the mean, and the number of colonies per colony health rating within this cohort are presented in the table. (PDF) [file pone.0237544.s003.pdf]

Supporting Figure S3.
Analysis of mean pathogen prevalence by colony health rating.

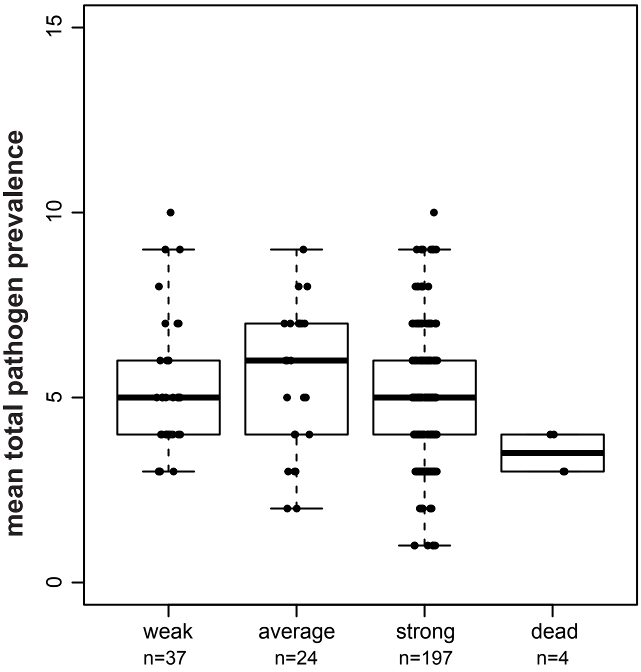

colony health rating	*mean pathogen prevalence*	*standard error (+/-)*	*sample size (n)*
weak	5.12	1.79	37
average	5.50	2.00	24
strong	4.91	1.92	197
dead	3.50	0.58	4
