# Supplementary material for: Longitudinal monitoring of honey bee colonies reveals dynamic nature of virus abundance and indicates a negative impact of Lake Sinai virus 2 on colony health
Source: PLoS One. 2020 Sep 8;15(9):e0237544. doi: 10.1371/journal.pone.0237544 (PMC7478651; doi:10.1371/journal.pone.0237544)
Supplement: S20 Fig — The abundance of viruses in co-infected colonies was analyzed by calculating the correlation coefficients for each pair-wise comparison, which were reported as r values in Fig 9, the corresponding p-values are listed in each cell, bold numbers indicate significant correlations (p-value < 0.05). The shaded red circles represent negative correlations and blue circles represent positive correlations; darker hues and larger circles indicate stronger correlations. (PDF) [file pone.0237544.s020.pdf]

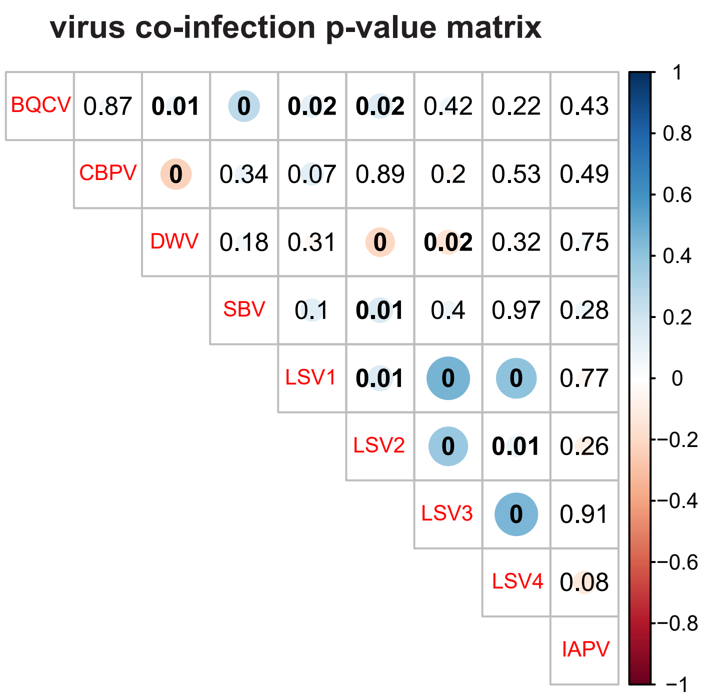

Supporting Figure S20. Virus co-infection correlation matrix p-values.
