# Supplementary material for: Longitudinal monitoring of honey bee colonies reveals dynamic nature of virus abundance and indicates a negative impact of Lake Sinai virus 2 on colony health
Source: PLoS One. 2020 Sep 8;15(9):e0237544. doi: 10.1371/journal.pone.0237544 (PMC7478651; doi:10.1371/journal.pone.0237544)

Supporting Figure S21.

August comparison of virus burden in honey bee colonies that died versus honey bee colonies that survived.

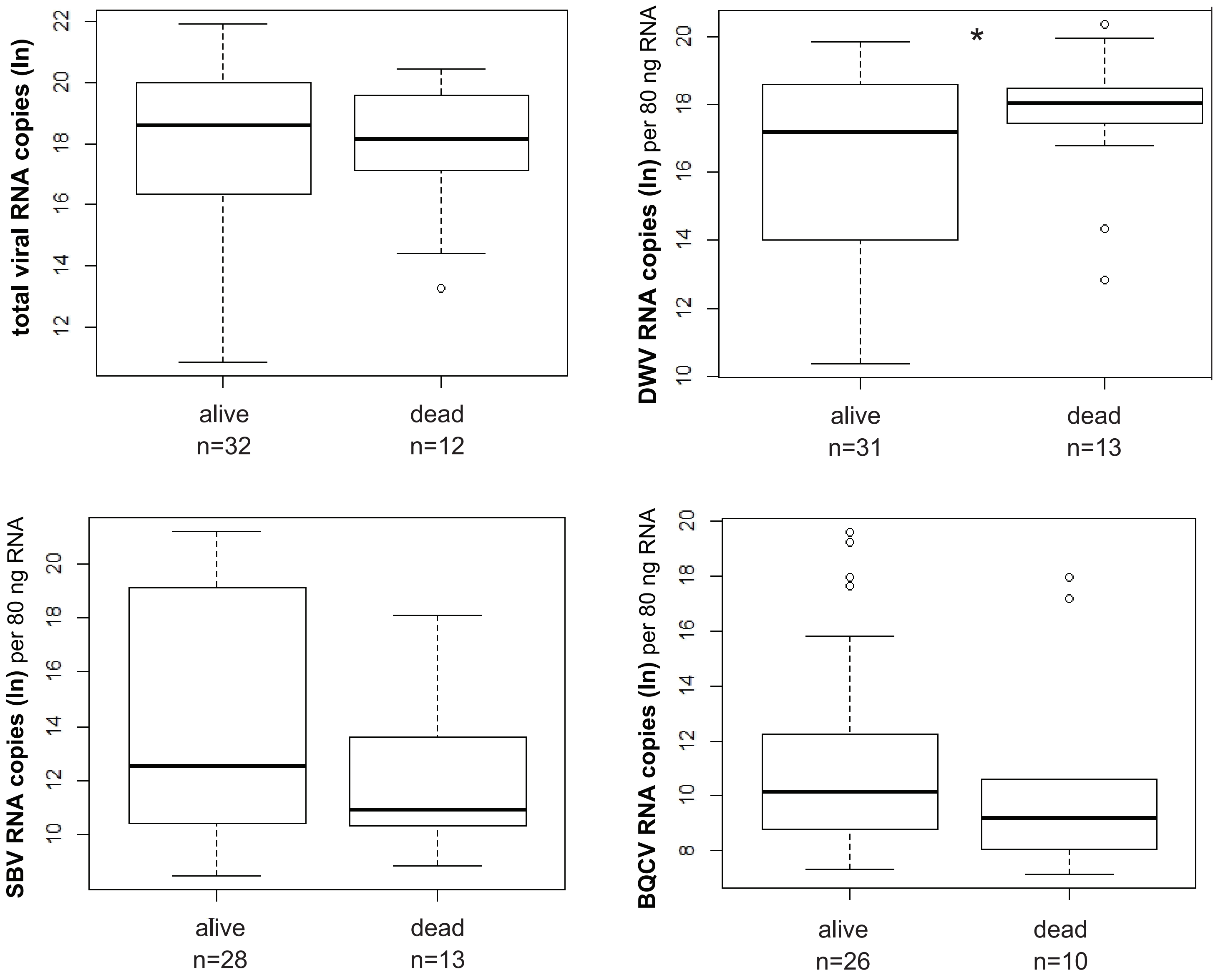

Supplement: S21 Fig — In total, 22 of the 50 monitored honey bee colonies died throughout the study, although samples were obtained for only 4 of those samples when they were dead. The majority of the colonies (i.e., 16 of 22) died between the August and October 2016 sampling dates. Therefore, individual and total viral abundance data from the August 2016 sample date were evaluated using a two-sided t-test to assess potential differences between colonies that were alive or dead in October 2016. These comparisons were carried out between colonies that tested positive for the viruses and therefore only comparisons with sufficient sample size (i.e., total abundance, DWV, SBV, and BQCV) are reported. This analysis indicated that there were no differences in virus abundance in the August sample date between colonies that were alive or dead by the October 2016 sampling date. (PDF) [file pone.0237544.s021.pdf]
