# Supplementary material for: Longitudinal monitoring of honey bee colonies reveals dynamic nature of virus abundance and indicates a negative impact of Lake Sinai virus 2 on colony health
Source: PLoS One. 2020 Sep 8;15(9):e0237544. doi: 10.1371/journal.pone.0237544 (PMC7478651; doi:10.1371/journal.pone.0237544)
Supplement: S4 Table — The percent difference attributed to each pathogen between consecutive sampling events was calculated using a similarity percentage (SIMPER) analysis of a Bray-Curtis dissimilarity matrix. The cumulative difference of the top three pathogens in comparisons between consecutive sampling events is reported as a percentage. (DOCX) [file pone.0237544.s025.docx]

|  | LSV2 | DWV | BQCV | DWV | DWV |  |
| --- | --- | --- | --- | --- | --- | --- |
| **Nov 2015** | BQCV | LSV2 | SBV | BQCV | BQCV |  |
|  | DWV | LSV1 | LSV2 | SBV | SBV |  |
|  | **54%** | **47%** | **44%** | **63%** | **62%** |  |
|  |  | LSV2 | LSV2 | LSV2 | DWV |  |
|  | **Mar 2016** | LSV3 | BQCV | DWV | LSV2 |  |
|  |  | LSV1 | DWV | BQCV | LSV3 |  |
|  |  | **49%** | **48%** | **60%** | **59%** |  |
|  |  |  | DWV | DWV | DWV |  |
|  |  | **Apr 2016** | LSV2 | LSV2 | LSV2 |  |
|  |  |  | BQCV | LSV1 | LSV1 |  |
|  |  |  | **43%** | **49%** | **51%** |  |
|  |  |  |  | DWV | DWV |  |
|  |  |  | **June 2016** | BQCV | BQCV |  |
|  |  |  |  | SBV | CBPV |  |
|  |  |  |  | **53%** | **52%** |  |
|  |  |  |  |  | BQCV |  |
|  |  |  |  | **Aug 2016** | SBV |  |
|  |  |  |  |  | LSV1 |  |
|  |  |  |  |  | **51%** |  |
|  |  |  |  |  |  |  |
|  |  |  |  |  | **Oct 2016** |  |
|  |  |  |  |  |  |  |
|  |  |  |  |  |  |  |
|  |  |  |  |  |  |  |

**Supporting Table S4. Similarity percentage analysis of the relative pathogen composition between sampling events.** The percent difference attributed to each pathogen between consecutive sampling events was calculated using a similarity percentage (SIMPER) analysis of a Bray-Curtis dissimilarity matrix. The cumulative difference of the top three pathogens in comparisons between consecutive sampling events is reported as a percentage.
