# Supplementary material for: Longitudinal monitoring of honey bee colonies reveals dynamic nature of virus abundance and indicates a negative impact of Lake Sinai virus 2 on colony health
Source: PLoS One. 2020 Sep 8;15(9):e0237544. doi: 10.1371/journal.pone.0237544 (PMC7478651; doi:10.1371/journal.pone.0237544)
Supplement: S5 Table — (DOCX) [file pone.0237544.s026.docx]

**S5 Table. Primers used in this study.**

| **Genome / Gene Name** | **NCBI #**  **GI #** | **Primer Name** | **Sequence (5’-3’)** | **Product Size (bp)** | **Reference** |
| --- | --- | --- | --- | --- | --- |
| **Ribosomal protein L8 (*Apis m.*)** | XM_393671.7 GI:571556074 | Rpl8Fw  Rpl8Rev | TGGATGTTCAACAGGGTTCATA  CTGGTGGTGGACGTATTGATAA | 121 | Evans et al. (2006) Insect Mol Bio |
| **Lake Sinai virus 1 (LSV1)** | HQ871931.2  GI:335057596 | qLSV1-F-2569  qLSV1-R-2743** | AGAGGTTGCACGGCAGCATG  GGGACGCAGCACGATGCTCA | 174 | Runckel, Flenniken (2011) PLoS One |
| **Lake Sinai virus 2 (LSV2)** | HQ888865.2  GI:335057589 | qLSV2-F-1722  qLSV2-R-1947** | CGTGCTGAGGCCACGGTTGT  GCGGTGTCGATCTCGCGGAC | 225 | Runckel, Flenniken (2011) PLoS One |
| **Lake Sinai Virus 3 (LSV3)** | \|  \| JQ480620.1 \| \| --- \| --- \|   GI:386289721 | LSV3-F-2186  LSV3-R-2429 | CGTGAGCACGATGAGTCAGT  TGGAGGTGCTTGTTGCATAA | 243 | Daughenbaugh et al. (2015) Viruses |
| **Lake Sinai virus 4 (LSV4)** | JX878492.1  GI:512134519 | LSV4-F-1896  LSV4-R-2278 | CCATCTCCTCATCCACGTTT  GATTCCCAAATCAGGCTCAA | 379 | Daughenbaugh et al. (2015) Viruses |
| **black queen cell virus (BQCV)** | AF183905.1  GI:8100530 | qBQCVorf2F_6664  qBQCVorf2R_6805 | TCCTCAAATCTGGAGCGAAC  GTATTCGCTGGCCGTAAAAC | 141 | Runckel, Flenniken (2011) PLoS One |
| **deformed wing virus (DWV)** | AY292384.1 GI:31540603 | DWV-F-1170  DWV-R-1364 | CTTACTCTGCCGTCGCCCA  CCGTTAGGAACTCATTATCGCG | 173 | Chen et al. (2005) J Invert Path |
| **sacbrood virus (SBV)** | AF092924.1 GI:4416206 | SBV_221-240_For  SBV_478-497_Rev | ACCAACCGATTCCTCAGTAG  TCTTCGTCCACTCTCATCAC | 257 | Grabensteiner et al. (2001) Clin and Diag Lab Immun |
| ***Lotmaria passim***  ***(****formerly Crithidia mellificae, sf)* | MK922078.1  Genome  PRJNA78249 | qCrFw1  qCrRev1 | TCCACTCTGCAAACGATGAC  GGGCCGAATGGAAAAGATAC | 153 | Runckel, Flenniken (2011) PLoS One |
| **acute bee paralysis virus (ABPV)** | AF150629.1 GI:10314009 | qABPV-F-5457  qABPV-R-5634 | GGATGAGAGAAGACCAATTG  CCAATCTTGGGAATAAACATTAGTTC | 177 | Highfield et al. (2009)  Appl Environ Micro |
| **chronic bee paralysis virus (CBPV)** | EU122229.1 GI:188543025 | CBPV-RdRp_F_307  CBPV-RdRp_R_454 | CCCAAAACCTGGAAGTCAT  CCCAAAACCTGGAAGTCAT | 148 | Primer3 Plus  (this work) |
| **Israeli acute paralysis virus (IAPV)** | EF219380.1 GI:126010924 | ORF2-F8092  ORF2-R8318 | CCAGCCGTGAAACATGTTCTTACC  ACATAGTTGCACGCCAATACGAGAAC | 226 | Palacios et al. (2008) JVI |
| **Kashmir bee virus (KBV)** | AY275710.1 GI:30793779 | KBV_F_5408  KBV_R_5534 | TGAACGTCGACCTATTGAAAAA  TCGATTTTCCATCAAATGAGC | 127 | Evans (2006) J Inv Path |
| ***N. ceranae*** | DQ673615.1  GI:110293152 | N ceranae F-4186  N ceranae R-4435 | CGGATAAAAGAGTCCGTTACC  TGAGCAGGGTTCTAGGGAT | 249 | Chen et al. (2008) J Inv Path |
